# Supplementary material for: Towards bioresource-based aggregation-induced emission luminogens from lignin β-O-4 motifs as renewable resources
Source: Nat Commun. 2023 Sep 28;14:6076. doi: 10.1038/s41467-023-41681-0 (PMC10539282; doi:10.1038/s41467-023-41681-0)
Supplement: Supplementary file 1 — Supplementary Information [file 41467_2023_41681_MOESM1_ESM.pdf]

Supplementary Information

Towards bioresource-based aggregation-induced emission luminogens from lignin β-O-4 motifs as renewable resources

Tenglong Guo,[1,#] Yuting Lin,[2,#] Deng Pan,[3,#] Xuedan Zhang,[2] Wenqing Zhu,[1] Xu-Min Cai,[2*] Genping Huang,[3*] Hua Wang,[1] Dezhu Xu,[1] Fritz E. Kühn,[4] Bo Zhang,[1*] and Tao Zhang[1*]

[1]CAS Key Laboratory of Science and Technology on Applied Catalysis, Dalian Institute of Chemical Physics, Chinese Academy of Sciences, Dalian 116023, China.

[2]Jiangsu Co-Innovation Center of Efficient Processing and Utilization of Forest Rescources, International Innovation Center for Forest Chemicals and Materials, College of Chemical Engineering, Nanjing Forestry University, Nanjing, 210037, China.

[3]Department of Chemistry, School of Science and Tianjin Key Laboratory of Molecular Optoelectronic Sciences, Tianjin University, Tianjin 300072, China.

[4]Molecular Catalysis, Catalysis Research Center and Department of Chemistry, Technical University of Munich, Lichtenbergstr. 4, D-85748, Garching bei München, Germany.

[#]These authors contributed equally: Tenglong Guo, Yuting Lin, Deng Pan.

E-mail: taozhang@dicp.ac.cn, bo.zhang@dicp.ac.cn, xumin.cai@njfu.edu.cn, gphuang@tju.edu.cn.

Table of Contents

1. Supplementary methods 3

   1.1 General 3

   1.2 Experimental methods 4

      1.2.1 Typical procedures of the reactions 4

      1.2.2 Optimizing the reaction parameters 7

2 Supplementary data 9

   2.1 Analytical data 9

   2.2 Copies of NMR spectra 15

   2.3 Computational details 47

   2.4 The photophysical property of carbazoles 48

3. Supplementary references 56

1. Supplementary methods

1.1 General

All lignin model compounds β-hydroxy ether substrates[1] and vanadium complexes[2] are prepared as described in the literature. The chemicals (>95% purity) are obtained commercially and used without further purification. ^{1}H and ^{13}C NMR spectra are recorded on a Bruker DRX-400 spectrometer and all chemical shift values refer to δ_{TMS} = 0.00 ppm or solvent peak is used as internal reference. Catalytic runs are monitored by Agilent Technologies 1260 Infinity with Eclipse XDB-C18 (4.6×150 mm) using an external standard calibration curve method. Analytical TLC plates, Sigma-Aldrich silica gel 60_{F200} are viewed by UV light (254 nm). Column chromatographic purifications are performed on SDZF silica gel 160. The carbazole derivatives are purified by column chromatography on silica gel (petroleum ether/ethyl acetate, 3:1) to afford pure products. The UV-visible absorption spectra of all the compounds are collected with a Shimadzu UV2450 spectrometer. Liquid products were quantified using a high-performance liquid chromatograph (HPLC, Agilent Technologies 1100 Series) equipped with a UV-vis Detector (λ=280 nm) and a C18 column (Agilent ZORBAX SB-C18, 5μm 4.6×150 mm). A mixed solution composed of acetonitrile and H_2O with a ration of 80:20 (v/v) was used as a mobile phase and the flow rate was 1.0 mL min^{-1}. Photoluminescence spectra are recorded on a FS5 spectrofluorometer (Edinburgh) for different water fraction (f_w) and on a Fluoromax-4 spectrofluorometer (Horiba) for solid. The absolute fluorescence quantum yields are measured by a Hamamatsu C9920-02G spectrometer by a Quanta-Φ integrating sphere. Single crystal data of **3d** is collected on a Xcalibur, Atlas, Gemini ultra diffractometer using Cu Kα radiation (1.54184 Å), which is kept at 293.00 (2) K. Single crystal data of **3p** and **3q** are collected on a XtaLAB Synergy, Dualflex, HyPix diffractometer using Cu Kα radiation (1.54184 Å), which are kept at 295.32 (10) and 295.24 (10) K, respectively. CCDC numbers: 2212869 (**3d**), 2212868 (**3p**), and 2212870 (**3q**).

1.2 Experimental methods

1.2.1 Typical procedures of the reactions

Typical procedures for the synthesis of β-hydroxy ether substrates (1a-1d).[1]

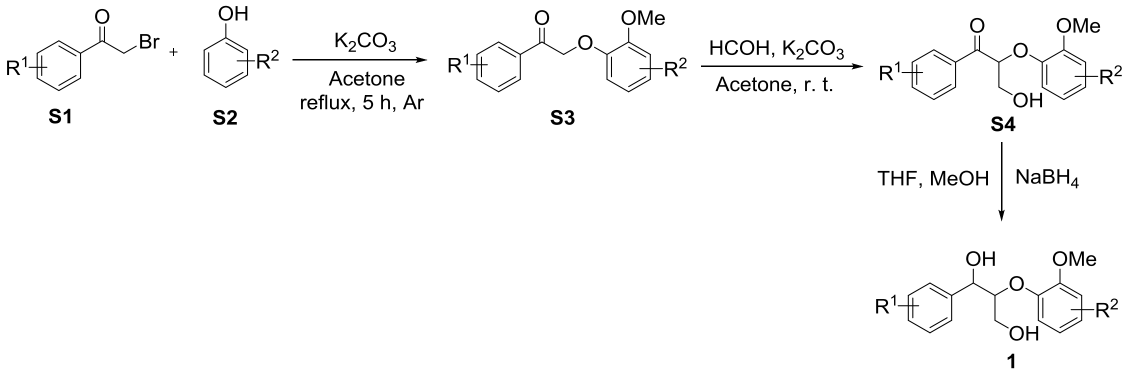

A mixture of phenol **S2** (0.5 mmol) and K_2CO_3 (0.6 mmol) in acetone (200 mL) was stirred at room temperature for 30 min and then a solution of 2-bromoacetophenone derivatives **S1** (0.5 mmol) in acetone (100 mL) was added dropwise. The resulting mixture was heated to reflux and stirred overnight. The resulting mixture was hearted to reflux and stirred overnight. The reaction mixture was cooled to room temperature and filtered; the filtrate was concentrated in vacuum. The residue was dissolved with ethyl acetate and washed with 5% NaOH. The organic layer was dried over anhydrous Na_2SO_4 and filtered, the filtrate was concentrated in vacuum. The crude product was recrystallization with ethanol to afford white solid **S3**.

A mixture of **S3** (0.5 mmol) and K_2CO_3 (0.6 mmol) in a EtOH and acetone (20 mL, v:v = 1:1) was stirred at room temperature and then aqueous formaldehyde solution (37%, 0.9 mmol) was added dropwise. The resulting mixture was stirred overnight. The reaction mixture was filtered, the filtrate was concentrated in vacuum. The residue was purified by column chromatography on silica gel (*n*-hexane : ethyl acetate, v:v = 2:1) to afford light yellow oil **S4**.

The compound **S4** (0.3 mmol) was dissolved with THF (20 mL) and MeOH (10 mL) and stirred at ice-water bath. $NaBH_4$ (0.36 mmol) was added portion wise, then the mixture was warmed to room temperature and stirred overnight. Saturated NH_4Cl aq. was added (pH = 5-6) and stirred for 1 hour. The resulting mixture was extracted with CH_2Cl_2 and then combined organic layers were washed with brine, dried over anhydrous Na_2SO_4 and filtered. The filtrate was concentrated in vacuum and the residue was purified by column chromatography on silica gel (CH_2Cl_2: MeOH, v:v = 40:1) to afford the products **1**. The NMR data and spectra of **1a**-**1d** are in the supplementary data and supplementary Figs. 2-5.

Typical procedures for the synthesis of V-complex catalyst.[2]

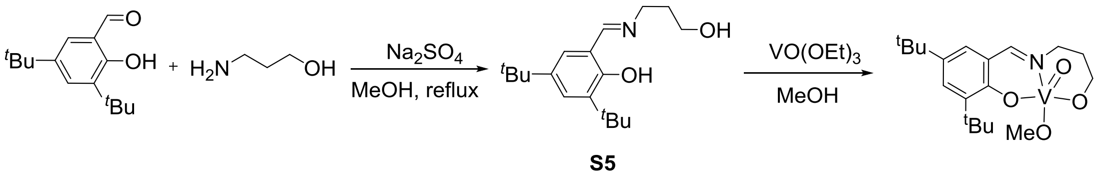

To a solution of the 3-aminopropan-1-ol (3.0 mmol) in MeOH (15 mL) were added the 3,5-di-tert-butyl-2-hydroxybenzaldehyde (3.0 mmol) and Na_2SO_4 (24 mmol), and the reaction mixture was refluxed overnight under nitrogen. The reaction mixture was cooled to room temperature, filtered, and concentrated to give yellow oil **S5**.

To a solution of the ligand **S5** in MeOH (10 mL) was added $VO(OEt)_3$ (0.50 mmol) dropwise. The reaction mixture was stirred for 10 min then concentrated under reduced pressure. The residue was dissolved in MeOH (2 mL) and recrystallized at -20 ^oC. The NMR data and spectra of V-complex catalyst are in the supplementary data and supplementary Fig. 6.

Typical procedures for the synthesis of 3-alkenylated indoles 2[3].

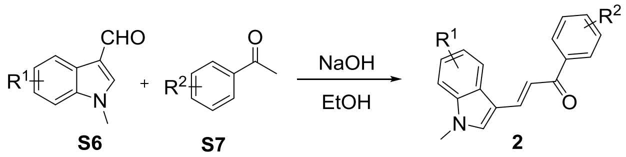

Indole-3-carbaldehyde derivatives **S6** (5 mmol) and acetophenone derivatives **S7** (5 mmol) were dissolved in ethanol (40 mL) and NaOH (600 mg, 15 mmol) was added. After the reaction was completed (24 h), the reaction mixture was filtered and washed with water and cool ethanol. The crude product was purified by recrystallization from ethanol and dichloromethane to give pure compounds **2**. The NMR data and spectra of V-complex catalyst are in the supplementary data and supplementary Figs. 7-12.

Typical produce in control experiment Equation 1: lignin model compound **1d** (0.4 mmol), V-based catalyst (10 mol%) and toluene (4 mL) are added into pressure tube (35 mL). The mixture is sealed and heated to 140 ^oC for 20 h. After reaction, the solution is cooled to room temperature. The crude products are purified by column chromatography using petroleum ether/ethyl acetate (9:1) to give 51% yield of **5a** and 60% yield of **4b**.

Typical produce in control experiment Equation 2: 1-phenylprop-2-en-1-one (0.4 mmol), alkenylated indole **2a** (0.2 mmol), V-based catalyst (10 mol%) and toluene (4 mL) are added into pressure tube (35 mL). The mixture is sealed and heated to 140 ^oC for 20 h. After reaction, the solution is cooled to room temperature. The **3d** product is purified by column chromatography using petroleum ether/ethyl acetate (3:1) to give 45% yield of **3d**.

Typical produce in control experiment Equation 3: 1-phenylprop-2-en-1-one (0.4 mmol), alkenylated indole **2a** (0.2 mmol), and toluene (4 mL) were added into pressure tube (35 mL). The mixture is sealed

and heated to 140 ^{o}C for 20 h. After reaction, the solution is cooled to room temperature. The **3d** product is detected by HPLC in 5% yield**.**

Typical produce in control experiment Equation 4: Under argon atmosphere, 1-phenylprop-2-en-1-one (0.4 mmol), alkenylated indole **2a** (0.2 mmol), and toluene (4 mL) were added into pressure tube (35 mL). The mixture is sealed and heated to 140 ^{o}C for 20 h. After reaction, the solution is cooled to room temperature. The **3d** product is purified by column chromatography using petroleum ether/ethyl acetate (3:1) to give 37% yield of **3d.**

Typical produce in control experiment Equation 5: Under argon atmosphere, 1-phenylprop-2-en-1-one (0.4 mmol), alkenylated indole **2a** (0.2 mmol), and toluene (4 mL) were added into pressure tube (35 mL). The mixture is sealed and heated to 140 ^{o}C for 20 h. After reaction, the solution is cooled to room temperature. The **3d** product is detected by HPLC in 3% yield**.**

Typical produce in control experiment of supplementary Fig. 1: lignin model compound **1d** (0.4 mmol), V-based catalyst (10 mol%), N-H free indole (((*E*)-3-(1H-indol-3-yl)-1-phenylprop-2-en-1-one)) and toluene (4 mL) are added into pressure tube (35 mL). The mixture is sealed and heated to 140 ^{o}C for 20 h. After reaction, the solution is cooled to room temperature. The crude products are purified by column chromatography using petroleum ether/ethyl acetate (9:1) to give 21% yield of ((*E*)-3-(1-(3-oxo-3-phenylpropyl)-1H-indol-3-yl)-1-phenylprop-2-en-1-one. NMR spectra are shown below in supplementary Fig.35.

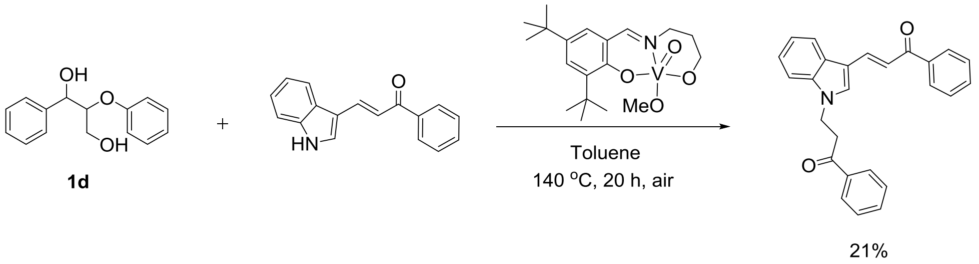

Supplementary Fig. 1. Control experiment.

1.2.2 **Optimizing the reaction parameters**

Supplementary Table 1. Synthesis of carbazole derivatives **3a** under different conditions.[a]

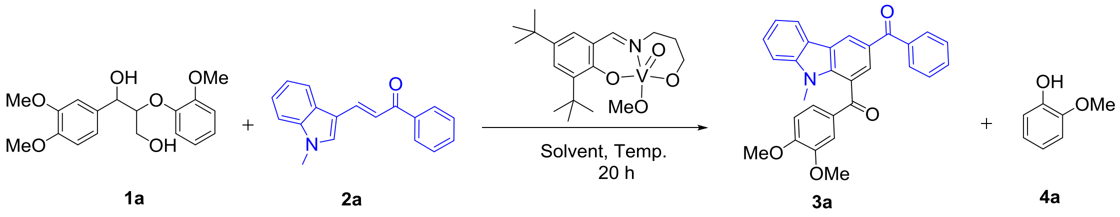

Entry	Solvent	Temp (°C)	**3a** Yield [%][b]	**4a** Yield [%][b]
1	**Toluene**	**140**	**89(91)** [c]	**99**
2	DMF	140	64	55
3	CH_3CN	140	15	73
4	CH_3OH	140	5	--
5	THF	140	--	71
6	H_2O	140	23	53
7	Toluene	130	80	95
8	Toluene	120	76	99
9[d]	Toluene	140	55	36
10[e]	Toluene	140	--	--

[a] Reaction conditions: **1a** (0.2 mmol), **2a** (0.1 mmol), V-based complex (10 mol%), solvent (2 mL) in air, reaction time (t) = 20 h, the yields of **3a** and **4a** are calculated based on the amounts of **2a** and **1a**, respectively; [b]Yields are determined by HPLC with an external standard method; [c]Isolated yield; [d]10 mol% $VO(OEt)_3$ as catalyst,15 mol% 2,2'-bipyridine as ligand; [e]Without catalyst.

Supplementary Table 2. Synthesis of carbazole derivatives **3a** under variation of catalyst loading.[a]

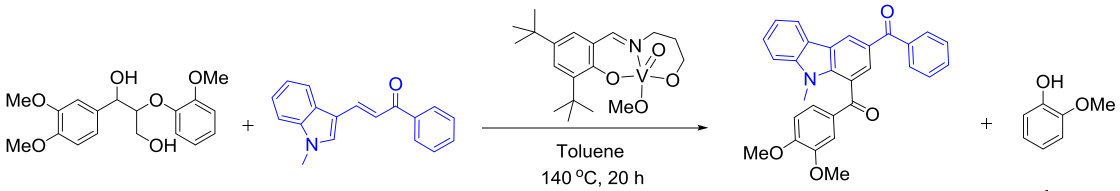

Entry	Cat. Loading (mol%)	**3a** Yield [%][b]	**4a** Yield [%][b]
1	1	16	28
2	5	28	30
3	10	89	99
4	15	70	64

[a]Reaction Conditions: **1a** (0.2 mmol), **2a** (0.1 mmol), V-based complex (x mol%), toluene (2 mL) in air, reaction time (t) = 20 h, the yields of **3a** and **4a** are calculated based on the amounts of **2a** and **1a**, respectively; [b]Yields are determined by HPLC with an external standard method.

Supplementary Table 3. Synthesis of carbazole derivatives **3a** under variation of substrates stoichiometry.[a]

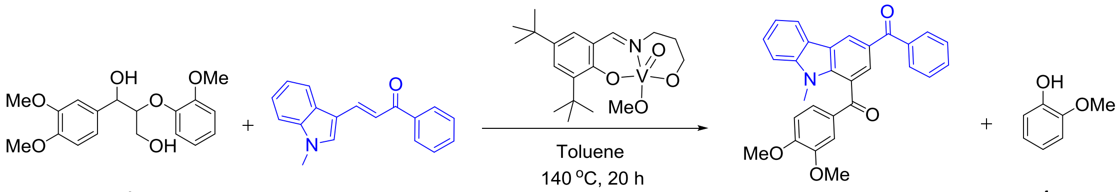

Entry	**1a (mmol)**	**2a (mmol)**	**1a:2a**	**3a** Yield [%][b]	**4a** Yield [%][b]
1	0.1	0.1	1:1	48	79
2	0.2	0.1	2:1	89	99
3	0.1	0.2	1:2	42	83

[a]Reaction Conditions: V-based complex (10 mol%), toluene (2 mL) in air, reaction time (t) = 20 h, the yields of **3a** and **4a** are calculated based on the amounts of **2a** and **1a**, respectively; [b]Yields are determined by HPLC with an external standard method.

2 Supplementary data

2.1 Analytical data

1-(3,4-Dimethoxyphenyl)-2-(2-methoxyphenoxy)propane-1,3-diol (1a): ^{1}H NMR (400 MHz, $CDCl_3$) δ 7.04 (t, *J* = 7.5 Hz, 1 H, aromatic CH), 6.93 (m, 5 H, aromatic CH), 6.82 (d, *J* = 8.2 Hz, 1 H, aromatic CH), 4.97 (d, *J* = 4.5 Hz, 1 H, CH), 4.15 (dd, *J* = 8.4, 5.0 Hz, 1 H, CH), 3.95-3.89 (m, 1 H, CH), 3.89-3.80 (m, 9 H, $3XOCH_3$), 3.66 (d, *J* = 9.5 Hz, 1 H, CH). HRMS Calcd for $C_{18}H_{22}NaO_6$ $[M+Na]^+$: 357.1314; Found: 357.1359.

2-(2-Methoxyphenoxy)-1-(4-methoxyphenyl)propane-1,3-diol (1b): ^{1}H NMR (400 MHz, $CDCl_3$) δ 7.36 (m, 1.24 H, major diastereomers), 7.30 (m, 0.78 H, minor diastereomers), 7.12 (m, 0.66 H, minor diastereomers), 7.04 (m, 1.08 H, both diastereomers), 6.97-6.86 (m, 4.47 H, both diastereomers), 4.98 (m, 1 H, both diastereomers), 4.15 (m, 0.49 H, minor diastereomers), 4.03 (m, 0.72 H, major diastereomers), 3.91 (m, 2.22 H, major diastereomers), 3.87 (m, 1.32 H, minor diastereomers), 3.80 (d, *J* = 1.1 Hz, 3 H, both diastereomers), 3.63 (m, 1.04 H, both diastereomers), 3.45 (m, 0.63 H, minor diastereomers), 2.75 (br s, 2.03 H, both diastereomers). HRMS Calcd for $C_{17}H_{20}NaO_5$ $[M+Na]^+$: 327.1208; Found: 327.1235.

1-(4-Hydroxy-3-methoxyphenyl)-2-(2-methoxyphenoxy)propane-1,3-diol (1c): ^{1}H NMR (400 MHz, $CDCl_3$) δ 7.05 (m, 1 H, aromatic CH), 6.96 (m, 2 H, aromatic CH), 6.92 (m, 2 H, aromatic CH), 6.87 (m, 1 H, aromatic CH), 6.82 (m, 1 H, aromatic CH), 5.71 (s, 1 H, ArO*H*), 4.96 (d, *J* = 4.7 Hz, 1 H, CH), 4.15 (m, 1 H, CH), 3.92 (m, 1 H, CH), 3.87 (m, 6 H, $2XOCH_3$), 3.67 (m, 1 H, CH), 2.90 (s, 2 H, 2XOH). HRMS Calcd for $C_{17}H_{20}NaO_6$ $[M+Na]^+$: 343.1158; Found: 343.1218.

2-Phenoxy-1-phenylpropane-1,3-diol (1d): ^{1}H NMR (400 MHz, $CDCl_3$) δ 7.38-7.11 (m, 7 H, both diastereomers), 6.93-6.78 (m, 3 H, both diastereomers), 4.98 (d, *J* = 5.2 Hz, 0.29 H, minor diastereomers), 4.94 (d, *J* = 6.4 Hz, 0.69 H, major diastereomers), 4.32 (m, 1 H, both diastereomers), 3.84 (dd, *J* = 12.1, 4.6 Hz, 0.32 H, minor diastereomers), 3.77-3.66 (m, 1 H, both diastereomers), 3.46 (dd, *J* = 12.1, 4.1 Hz, 1 H, major diastereomers), 2.55 (s, 2 H, both diastereomers). HRMS Calcd for $C_{15}H_{16}NaO_3$ $[M+Na]^+$: 267.0997; Found: 267.1012.

V-complex catalyst : A solution of V-complex catalyst in MeOD showed a mixture of two compounds by both ^{1}H NMR (ca. 11:1 ratio). ^{1}H NMR of the major component (MeOD, 400 MHz) δ 8.52 (s, 1 H, -N=C-*H*), 7.57 (d, *J* = 2.5 Hz, 1 H, aromatic CH), 7.33 (d, *J* = 2.5 Hz, 1 H, aromatic CH), 5.53 (td, *J* = 10.9, 2.6 Hz, 1 H), 4.24 (t, *J* = 12.3 Hz, 1 H), 4.05 (d, *J* = 12.4 Hz, 1 H), 3.68 (t, *J* = 6.4 Hz, 1H), 3.35 (s, 3 H, OMe), 2.34-2.24 (m, 1 H), 1.98 (m, 1 H), 1.52-1.42 (m, 9 H, $C(CH_3)_3$), 1.33 (s, 9 H, $C(CH_3)_3$).
Distinguishable peaks of the minor component in ^{1}H NMR (MeOD, 400 MHz) δ 8.44 (s, 0.10 H), 7.37 (d, J = 2.5 Hz, 0.1 H), 7.17 (d, J = 2.4 Hz, 0.1 H), 3.68 (m, 0.45 H), 1.92 (m, 0.26 H), 1.42 (s, 1 H), 1.30 (s, 1 H);

(E)-3-(1,5-Dimethyl-1H-indol-3-yl)-1-phenylprop-2-en-1-one (2c): Yellow solid, m.p.: 119-121 °C. ^{1}H NMR (400 MHz, $CDCl_3$) ^{1}H NMR (400 MHz, $CDCl_3$) δ 8.11-8.03 (m, 3 H, m, CH=CHCOPh and aromatic CH), 7.78 (s, 1 H, aromatic CH), 7.60-7.48 (m, 4 H, aromatic CH and CH=CHCOPh), 7.33 (s, 1 H, aromatic CH), 7.21 (d, J = 8.3 Hz, 1 H, aromatic CH), 7.15 (d, J = 8.4 Hz, 1 H, aromatic CH), 3.71 (s, 3 H, NCH_3), 2.55 (s, 3 H, CH3).^{13}C{^{1}H} (100 MHz, $CDCl_3$) δ 190.8 (Cq, C=O), 139.2, 136.6, 131.0, 126.3 and 112.4 (Cq each), 139.0, 134.8, 132.0, 128.5, 128.2, 124.7, 120.5, 116.6 and 109.8 (CH), 33.2 (NCH_3), 21.7 (CH_3). HRMS Calcd for $C_{19}H_{18}NO$ $[M+H]^+$: 276.1388; Found: 276.1383.

(E)-3-(1,6-Dimethyl-1H-indol-3-yl)-1-phenylprop-2-en-1-one (2d): Yellow solid, m.p.: 134-136 °C. ^{1}H NMR (400 MHz, $CDCl_3$) ^{1}H NMR (400 MHz, $CDCl_3$) δ 8.10-8.02 (m, 3 H, CH=CHCOPh and aromatic CH), 7.89 (d, J = 8.1 Hz, 1 H, aromatic CH), 7.59-7.47 (m, 4 H, aromatic CH and CH=CHCOPh), 7.40 (s, 1 H, aromatic CH), 7.19-7.11 (m, 2 H, aromatic CH), 3.80 (s, 3 H, NCH_3), 2.53 (s, 3 H, CH_3). ^{13}C{^{1}H} (100 MHz, $CDCl_3$) δ 191.0 (Cq, C=O), 139.4, 139.0, 124.0 and 113.1 (Cq each), 139.1, 134.6, 133.4, 132.2, 128.6, 128.4, 123.4, 120.7, 117.0 and 110.3 (CH), 33.4 (NCH_3), 22.0 (CH_3). HRMS Calcd for $C_{19}H_{18}NO$ $[M+H]^+$: 276.1388; Found: 276.1383.

(E)-3-(1-Methyl-1H-indol-3-yl)-1-(m-tolyl)prop-2-en-1-one (2h): Yellow solid, m.p.: 127-129 °C. ^{1}H NMR (400 MHz, $CDCl_3$) δ 8.10 (d, J = 15.5 Hz, 1 H, CH=CHCOPh), 8.04 (m, 1 H, aromatic CH), 7.87 (d, J = 8.3 Hz, 2 H, aromatic CH), 7.56 (d, J = 15.5 Hz, 1 H, CH=CHCOPh), 7.49 (s, 1 H, aromatic CH), 7.45-7.32 (m, 5 H, aromatic

CH), 3.86 (s, 3 H, NCH_3), 2.48 (s, 3 H, CH_3). $^{13}C\{^1H\}$ (100 MHz, $CDCl_3$) δ 191.1 (Cq, C=O), 139.3, 138.4, 126.3 and 113.2 (Cq each), 138.6, 134.6, 133.0, 129.0, 128.5, 125.6, 123.3, 121.7, 120.9, 117.4 and 110.3 (CH), 33.4 (NCH_3), 21.6 (CH_3). HRMS Calcd for $C_{19}H_{18}NO$ $[M+H]^+$: 276.1388; Found: 276.1388;.

(E)-3-(1-Methyl-1H-indol-3-yl)-1-(naphthalen-2-yl)prop-2-en-1-one (2o): Yellow solid, m.p.: 166-168 °C. ^1H NMR (400 MHz, $CDCl_3$) δ 8.57 (s, 1 H, aromatic CH), 8.15 (m, 2 H, CH=CHCOPh and aromatic CH), 8.07 (m, 1 H, aromatic CH), 8.03 (d, J = 7.6 Hz, 1 H, aromatic CH), 7.95 (d, J = 8.6 Hz, 1 H, aromatic CH), 7.91 (d, J = 7.7 Hz, 1 H, aromatic CH), 7.70 (d, J = 15.5 Hz, 1 H, CH=CHCOPh), 7.59 (m, 2 H, aromatic CH), 7.49 (s, 1 H, aromatic CH), 7.42-7.32 (m, 3 H, aromatic CH), 3.84 (s, 3 H, NCH_3). $^{13}C\{^1H\}$ (100 MHz, $CDCl_3$) δ 190.7 (Cq, C=O), 138.4, 136.6, 135.4, 132.8, 126.3, and 113.2 (Cq each), 138.8, 134.7, 129.6, 129.4, 128.5, 128.1, 127.9, 126.7, 124.8, 123.3, 121.7, 121.0, 117.2 and 110.3 (CH), 33.4 (NCH_3). HRMS Calcd for $C_{22}H_{18}NO$ $[M+H]^+$: 312.1388; Found: 312.1379;.

(E)-3-(1-Methyl-1H-indol-3-yl)-1-(4-(1,2,2-triphenylvinyl)phenyl)prop-2-en-1-one (2p): Yellow solid, m.p.: 113-115 °C. 1H NMR (400 MHz, $CDCl_3$) δ 8.07 (d, J = 15.5 Hz, 1 H, CH=CHCOPh), 7.99 (d, J = 7.1 Hz, 1 H, aromatic CH), 7.85 (d, J = 8.3 Hz, 2 H, aromatic CH), 7.52 (d, J = 15.5 Hz, 1 H, CH=CHCOPh), 7.43 (s, 1 H, aromatic CH), 7.32 (m, 3 H, aromatic CH), 7.20 (d, J = 8.3 Hz, 2 H, aromatic CH), 7.14 (m, 9 H, aromatic CH), 7.09 (m, 6 H, aromatic CH), 3.80 (s, 3 H, NCH_3). $^{13}C\{^1H\}$ (100 MHz, $CDCl_3$) δ 190.0 (Cq, C=O), 148.2, 143.5, 143.4, 143.3, 142.4, 140.2, 138.3, 136.9, 126.2 and 113.1 (Cq each), 138.3, 134.6, 131.6, 131.44, 131.42, 131.39, 128.0, 127.9, 127.9, 127.8, 126.9, 126.8, 123.2, 121.6, 120.9, 116.9 and 110.2 (CH), 33.3 (NCH_3). HRMS Calcd for $C_{38}H_{30}NO$ $[M+H]^+$: 516.2327; Found: 516.2327;.

(E)-1-Cyclopropyl-3-(1-methyl-1H-indol-3-yl)prop-2-en-1-one (2s): Yellow solid, m.p.: 133-135 °C. ^1H NMR (400 MHz, $CDCl_3$) δ 7.96 (d, J = 7.7 Hz, 1 H, aromatic CH), 7.86 (d, J = 15.9 Hz, 1 H, CH=CHCOPh), 7.34 (m, 3 H, aromatic CH), 7.28 (m, 1 H, aromatic CH), 6.93 (d, J = 15.9 Hz, 1 H, CH=CHCOPh), 3.79 (s, 3 H, NCH_3), 2.23 (td, J = 7.9, 4.0 Hz, 1 H, cyclopropyl CH), 1.21-1.13 (m, 2 H, cyclopropyl CH), 0.94 (td, J = 7.0, 3.6 Hz, 2 H, cyclopropyl CH). $^{13}C\{^1H\}$ (100 MHz, $CDCl_3$) δ 200.2 (Cq, C=O), 138.3, 126.2 and 112.4 (Cq each), 135.7, 133.9, 123.1, 121.7, 121.4, 120.7 and 110.1 (CH), 33.3 (NCH_3), 19.7 (cyclopropyl CH), 10.9 (cyclopropyl CH). HRMS Calcd for $C_{15}H_{16}NO$ $[M+H]^+$: 226.1232; Found: 226.1226;.

(3-Benzoyl-9-methyl-9H-carbazol-1-yl)(3,4-dimethoxyphenyl)methanone (3a) : White solid, m.p.: 153-155 °C. ^1H NMR (400 MHz, $CDCl_3$) δ 8.74 (d, J = 1.6 Hz, 1 H, aromatic CH), 8.15 (d, J = 7.8 Hz, 1 H, aromatic CH), 8.02 (d, J = 1.5 Hz, 1 H, aromatic CH), 7.84 (m, 2 H, aromatic CH), 7.70 (d, J = 1.9 Hz, 1 H, aromatic CH), 7.59 (m, 2 H, aromatic CH), 7.49 (m, 3 H, aromatic CH), 7.39 (m, 1 H, aromatic CH), 7.34 (t, J = 7.5 Hz, 1 H, aromatic CH), 6.86 (d, J = 8.4 Hz, 1 H, aromatic CH), 3.97 and 3.95 (s each, 3:3 H, 2×OCH_3), 3.69 (s, 3 H, NCH_3). $^{13}C\{^1H\}$ (100 MHz, $CDCl_3$) δ 196.0 and 194.8 (Cq each, C=O), 154.2, 149.6, 142.6, 141.1, 138.6, 130.9, 127.6, 124.7, 122.9 and 122.7 (Cq each), 132.2, 130.0, 129.4, 128.5, 127.3, 127.2, 125.3, 120.8, 120.7 111.3, 110.2 and 109.6 (CH), 56.3 and 56.2 (2×OCH_3), 33.0 (NCH_3). HRMS Calcd for $C_{29}H_{24}NO_4$ $[M+H]^+$: 450.1705; Found: 450.1708.

(3-Benzoyl-9-methyl-9H-carbazol-1-yl)(4-methoxyphenyl)methanone (3b): White solid. ^1H NMR (400 MHz, $CDCl_3$) δ 8.74 (d, J = 1.5 Hz, 1 H, aromatic CH), 8.14 (d, J = 7.7 Hz, 1 H, aromatic CH), 8.03 (d, J = 1.5 Hz, 1 H, aromatic CH), 7.95 (d, J = 8.9 Hz, 2 H, aromatic CH), 7.85 (d, J = 7.1 Hz, 2 H, aromatic CH), 7.61-7.52 (m, 2 H, aromatic CH), 7.50 (t, J = 7.5 Hz, 2 H, aromatic CH), 7.44 (d, J = 8.2 Hz, 1 H, aromatic CH), 7.33 (t, J = 7.4 Hz, 1 H, aromatic CH), 6.97 (d, J = 8.9 Hz, 2 H, aromatic CH), 3.88 (s, 3 H, OCH_3), 3.68 (s, 3 H, NCH_3). $^{13}C\{^1H\}$ (100 MHz, $CDCl_3$) δ 195.9 and 194.6 (Cq each, C=O), 164.2, 142.6, 140.9, 138.5, 130.7, 127.5, 124.6, 122.80 and 122.77 (Cq each), 133.1, 132.1, 130.0, 129.3, 128.4, 127.2, 125.2, 120.7, 120.6, 114.1 and 109.6 (CH), 55.7 (OCH_3), 32.9 (NCH_3).

(3-Benzoyl-9-methyl-9H-carbazol-1-yl)(3-hydroxy-4-methoxyphenyl)methanone (3c): White solid, m.p.: 122-124 °C. ^1H NMR (400 MHz, $CDCl_3$) δ 8.74 (d, J = 1.5 Hz, 1 H, aromatic CH), 8.15 (d, J = 7.7 Hz, 1 H, aromatic CH), 8.02 (d, J = 1.5 Hz, 1 H, aromatic CH), 7.88-7.81 (m, 2 H, aromatic CH), 7.69 (d, J = 1.7 Hz, 1 H, aromatic CH), 7.57 (m, 2 H, aromatic CH), 7.52-7.43 (m, 3 H, aromatic CH), 7.40-7.30 (m, 2 H, aromatic CH), 6.92 (d, J = 8.3 Hz, 1 H, aromatic CH), 6.43 (s, 1 H, OH), 3.96 (s, 3 H, OCH3), 3.69 (s, 3 H, NCH_3). $^{13}C\{^1H\}$ (100 MHz, $CDCl_3$) δ 196.1 and 194.8 (Cq each, C=O), 151.4, 147.1, 142.6, 141.0, 138.5, 130.6, 127.5, 124.7, 122.8 and 122.6 (Cq each), 132.1, 130.0, 129.4, 128.4, 127.7, 127.3, 125.3, 120.8, 120.7, 114.1, 111.2 and 109.6 (CH), 56.3 (OCH_3), 33.0 (NCH_3). HRMS Calcd for $C_{28}H_{22}NO_4$ $[M+H]^+$: 436.1549; Found: 436.1547.

(9-Methyl-9H-carbazole-1,3-diyl)bis(phenylmethanone) (3d): Yield 95%. ^1H NMR (400 MHz, $CDCl_3$), Pale yellow solid. δ 8.76 (d, J = 1.6 Hz, 1 H, aromatic CH), 8.16 (d, J = 7.8 Hz, 1 H, aromatic CH), 8.04 (d, J = 1.6 Hz, 1 H, aromatic CH), 7.98 (m, 2 H, aromatic CH), 7.84 (m, 2 H, aromatic CH), 7.64 (t, J = 7.4 Hz, 1 H, aromatic CH), 7.58 (m, 2 H, aromatic CH), 7.50 (m, 5 H, aromatic CH), 7.35 (t, J = 7.4 Hz, 1 H, aromatic CH), 3.69 (s, 3

H, NCH_3). ^{13}C NMR (100 MHz, $CDCl_3$) δ 195.83 and 195.79 (Cq each, C=O), 142.6, 141.1, 138.4, 137.7, 127.5, 124.8, 122.7 and 122.4 (Cq each), 133.8, 132.1, 130.7, 129.9, 129.8, 128.8, 128.4, 127.3, 125.6, 120.8, 120.6 and 109.6 (aromatic CH), 33.2 (NCH_3). Single crystal of 3d were obtained by the slow evaporation of its ACN solution.

(3-Benzoyl-9-benzyl-9H-carbazol-1-yl)(3,4-dimethoxyphenyl)methanone (3e): White solid, m.p.: 177-179 ^oC. ^1H NMR (400 MHz, $CDCl_3$) δ 8.78 (d, J = 1.6 Hz, 1 H, aromatic CH), 8.20 (d, J = 7.7 Hz, 1 H, aromatic CH), 7.88 (d, J = 1.6 Hz, 1 H, aromatic CH), 7.83 (d, J = 7.1 Hz, 2 H, aromatic CH), 7.55 (m, 2 H, aromatic CH), 7.51 (m, 1 H, aromatic CH), 7.46 (t, J = 7.6 Hz, 2 H, aromatic CH), 7.36 (t, J = 7.1 Hz, 1 H, aromatic CH), 7.28 (d, J = 1.8 Hz, 1 H, aromatic CH), 6.99 (m, 1 H, aromatic CH), 6.96-6.87 (m, 3 H, aromatic CH), 6.68 (d, J = 7.1 Hz, 2 H, aromatic CH), 6.63 (d, J = 8.4 Hz, 1 H, aromatic CH), 5.57 (s, 2 H, NCH_2Ph), 3.89 and 3.85 (s each, 3:3 H, 2×OCH_3). 13C{^{1}H} (100 MHz, $CDCl_3$) δ 195.8 and 194.4 (Cq each, C=O), 153.4, 148.7, 142.8, 139.8, 138.3, 136.0, 130.3, 125.3, 125.2, 123.4 and 122.8 (Cq each), 132.1, 129.9, 129.4, 128.3, 127.6, 127.4, 127.3, 126.7, 126.5, 121.0, 120.6, 111.5, 110.0 and 109.8 (CH), 56.1 and 56.0 (2×OCH_3), 48.2 (NCH_2Ph). HRMS Calcd for $C_{35}H_{28}NO_4$ $[M+H]^+$: 526.2018; Found: 526.2015.

(3-Benzoyl-6,9-dimethyl-9H-carbazol-1-yl)(3,4-dimethoxyphenyl)methanone (3f): White solid, m.p.: 185-187 ^oC. ^1H NMR (400 MHz, $CDCl_3$) δ 8.69 (d, J = 1.2 Hz, 1 H, aromatic CH), 8.01 (d, J = 1.2 Hz, 1 H, aromatic CH), 7.93 (s, 1 H, aromatic CH), 7.83 (d, J = 7.2 Hz, 2 H, aromatic CH), 7.69 (d, J = 1.5 Hz, 1 H, aromatic CH), 7.58 (t, J = 7.4 Hz, 1 H, aromatic CH), 7.49 (m, 2 H, aromatic CH), 7.37 (m, 3 H, aromatic CH), 3.96 and 3.94 (s, 3 H, 2×OCH_3), 3.65 (s, 3 H, NCH_3), 2.54 (s, 3 H, CH_3). $^{13}C\{^1H\}$ (100 MHz, $CDCl_3$) δ 195.9 and 194.8 (Cq each, C=O), 154.1, 149.5, 141.1, 140.9, 138.6, 130.9, 130.3, 129.3, 124.4, 122.9 and 122.6 (Cq each), 132.0, 130.0, 128.6, 128.4, 127.2, 127.1, 125.3, 120.5, 111.3, 110.1 and 109.3 (CH), 56.3 and 56.2 (2×OCH_3), 33.0 (NCH_3), 21.4 (CH_3). HRMS Calcd for $C_{30}H_{26}NO_4$ $[M+H]^+$: 464.1862; Found: 464.1861.

(3-Benzoyl-7,9-dimethyl-9H-carbazol-1-yl)(3,4-dimethoxyphenyl)methanone (3g): White solid, m.p.: 170-172 ^oC. ^1H NMR (400 MHz, $CDCl_3$) δ 8.71 (d, J = 1.3 Hz, 1 H, aromatic CH), 8.02 (d, J = 8.1 Hz, 2 H, aromatic CH), 7.86 (d, J = 7.2 Hz, 2 H, aromatic CH), 7.72 (d, J = 1.7 Hz, 1 H, aromatic CH), 7.59 (t, J = 7.4 Hz, 1 H, aromatic CH), 7.50 (m, 2 H, aromatic CH), 7.42 (m, 1 H, aromatic CH), 7.27 (m, 1 H, aromatic CH), 7.17 (d, J = 8.0 Hz, 1 H, aromatic CH), 6.87 (d, J = 8.4 Hz, 1 H, aromatic CH), 3.98 and 3.96 (s each, 3:3 H, 2×OCH3), 3.67 (s, 3 H, CH_3), 2.58 (s, 3 H, NCH_3). $^{13}C\{^1H\}$ (100 MHz, $CDCl_3$) δ 195.9 and 194.7 (Cq each, C=O), 154.0, 149.4, 143.0, 141.0, 138.5, 137.6, 130.8, 127.3, 124.7, 122.4 and 120.4 (Cq each), 132.0, 129.9, 128.9, 128.3, 127.0, 124.8, 122.2, 120.2, 111.3, 110.0 and 109.7 (CH), 56.2 and 56.1 (2×OCH_3), 32.8 (NCH_3), 22.3 (CH_3). HRMS Calcd for $C_{30}H_{26}NO_4$ $[M+H]^+$: 464.1862; Found: 464.1861.

(3-Benzoyl-6-methoxy-9-methyl-9H-carbazol-1-yl)(3,4-dimethoxyphenyl)methanone (3h): White solid, m.p.: 185-187 ^oC. ^1H NMR (400 MHz, $CDCl_3$) δ 8.68 (d, J = 1.2 Hz, 1 H, aromatic CH), 7.99 (d, J = 1.2 Hz, 1 H, aromatic CH), 7.81 (d, J = 7.3 Hz, 2 H, aromatic CH), 7.68 (d, J = 1.6 Hz, 1 H, aromatic CH), 7.57 (d, J = 2.2 Hz, 1 H, aromatic CH), 7.54 (t, J = 7.4 Hz, 1 H, aromatic CH), 7.46 (t, J = 7.5 Hz, 2 H, aromatic CH), 7.38 (m, 1 H, aromatic CH), 7.29 (d, J = 8.9 Hz, 1 H, aromatic CH), 7.12 (m, 1 H, aromatic CH), 6.83 (d, J = 8.4 Hz, 1 H, aromatic CH), 3.94, 3.91 and 3.88 (s each, 3:3:3 H, 3×OCH_3), 3.61 (s, 3 H, NCH_3). $^{13}C\{^1H\}$ (100 MHz, $CDCl_3$) δ 195.7 and 194.6 (Cq each, C=O), 154.8, 154.0, 149.4, 141.2, 138.5, 137.3, 130.8, 126.9, 124.3, 123.1 and 122.5 (Cq each), 132.0, 129.9, 129.3, 128.3, 127.0, 125.3, 116.4, 111.3, 110.3, 110.1 and 103.0 (CH), 56.2, 56.1 and 56.0 (3×OCH_3), 33.0 (NCH_3). HRMS Calcd for $C_{30}H_{26}NO_5$ $[M+H]^+$: 480.1811; Found: 480.1802.

(3-Benzoyl-6-chloro-9-methyl-9H-carbazol-1-yl)(3,4-dimethoxyphenyl)methanone (3i): White solid, m.p.: 219-221 ^oC. ^1H NMR (400 MHz, $CDCl_3$) δ 8.61 (d, J = 1.5 Hz, 1 H, aromatic CH), 8.03 (m, 2 H, aromatic CH), 7.85-7.77 (m, 2 H, aromatic CH), 7.68 (d, J = 1.8 Hz, 1 H, aromatic CH), 7.56 (t, J = 7.4 Hz, 1 H, aromatic CH), 7.47 (t, J = 7.5 Hz, 2 H, aromatic CH), 7.40 (m, 2 H, aromatic CH), 7.30 (d, J = 8.7 Hz, 1 H, aromatic CH), 6.85 (d, J = 8.4 Hz, 1 H, aromatic CH), 3.95 and 3.92 (s each, 3:3 H, 2×OCH_3), 3.63 (s, 3 H, NCH_3). $^{13}C\{^1H\}$ (100 MHz, $CDCl_3$) δ 195.5 and 194.2 (Cq each, C=O), 154.1, 149.4, 141.2, 140.7, 138.2, 130.5, 127.7, 126.2, 123.7, 123.4 and 122.9 (Cq each), 132.1, 129.8, 129.7, 128.4, 127.2, 127.1, 125.4, 120.2, 111.2, 110.6 and 110.1 (CH), 56.2 and 56.1 (2×OCH_3), 33.0 (NCH_3). HRMS Calcd for $C_{29}H_{23}ClNO_4$ $[M+H]^+$: 484.1316; Found: 484.1314.

(1-(3,4-Dimethoxybenzoyl)-9-methyl-9H-carbazol-3-yl)(o-tolyl)methanone (3j): White solid, m.p.: 109-111 ^oC. ^1H NMR (400 MHz, $CDCl_3$) δ 8.65 (d, J = 1.6 Hz, 1 H, aromatic CH), 8.10 (d, J = 7.8 Hz, 1 H, aromatic CH), 8.03 (d, J = 1.6 Hz, 1 H, aromatic CH), 7.69 (d, J = 1.9 Hz, 1 H, aromatic CH), 7.54 (m, 1 H, aromatic CH), 7.43 (d, J = 8.2 Hz, 1 H, aromatic CH), 7.39 (m, 3 H, aromatic CH), 7.30 (m, 3 H, aromatic CH), 6.86 (d, J = 8.4 Hz, 1 H, aromatic CH), 3.96 and 3.95 (s each, 3:3 H, 2×OCH_3), 3.66 (s, 3 H, NCH_3), 2.36 (s, 3 H, CH_3). $^{13}C\{^1H\}$ (100 MHz, $CDCl_3$) δ 197.7 and 194.7 (Cq each, C=O), 154.1, 149.5, 142.5, 141.3, 139.2, 136.4, 130.8, 128.0, 124.7, 122.81 and 122.79 (Cq each), 131.0, 130.1, 129.0, 128.2, 127.2, 127.0, 125.3, 125.2, 120.8, 120.6,

111.2, 110.1 and 109.6 (CH), 56.2 and 56.1 (2×OCH_3), 32.9 (NCH_3), 20.0 (CH_3). HRMS Calcd for $C_{30}H_{26}NO_4$ $[M+H]^+$: 464.1862; Found: 464.1858.

(1-(3,4-Dimethoxybenzoyl)-9-methyl-9H-carbazol-3-yl)(m-tolyl)methanone (3k): White solid, m.p.: 202-154 °C. ^{1}H NMR (400 MHz, $CDCl_3$) δ 8.74 (d, J = 1.6 Hz, 1 H, aromatic CH), 8.15 (d, J = 7.7 Hz, 1 H, aromatic CH), 8.01 (d, J = 1.6 Hz, 1 H, aromatic CH), 7.70 (d, J = 1.8 Hz, 1 H, aromatic CH), 7.66 (s, 1 H, aromatic CH), 7.61 (d, J = 6.7 Hz, 1 H, aromatic CH), 7.54 (d, J = 7.2 Hz, 1 H, aromatic CH), 7.45 (d, J = 8.2 Hz, 1 H, aromatic CH), 7.39 (m, 3 H, aromatic CH), 7.33 (t, J = 7.4 Hz, 1 H, aromatic CH), 6.86 (d, J = 8.4 Hz, 1 H, aromatic CH), 3.97 and 3.95 (s each, 3:3 H, 2×OCH_3), 3.68 (s, 3 H, NCH3), 2.42 (s, 3 H, CH3). $^{13}C\{^1H\}$ (100 MHz, $CDCl_3$) δ 196.2 and 194.7 (Cq each, C=O), 154.1, 149.5, 142.6, 141.0, 138.5, 138.3, 130.9, 127.6, 124.6, 122.8 and 122.6 (Cq each), 132.9, 130.4, 129.4, 128.2, 127.2, 127.2, 127.1, 125.2, 120.7, 120.6, 111.3, 110.1 and 109.6 (CH), 56.3 and 56.2 (2×OCH_3), 33.0 (NCH_3), 21.5 (CH_3). HRMS Calcd for $C_{30}H_{26}NO_4$ $[M+H]^+$: 464.1862; Found: 464.1858.

(1-(3,4-Dimethoxybenzoyl)-9-methyl-9H-carbazol-3-yl)(p-tolyl)methanone (3l): White solid, m.p.: 202-204 °C. ^{1}H NMR (400 MHz, $CDCl_3$) δ 8.72 (d, J = 1.3 Hz, 1 H, aromatic CH), 8.12 (d, J = 7.7 Hz, 1 H, aromatic CH), 8.01 (d, J = 1.3 Hz, 1 H, aromatic CH), 7.75 (d, J = 8.0 Hz, 2 H, aromatic CH), 7.70 (d, J = 1.6 Hz, 1 H, aromatic CH), 7.52 (t, J = 7.6 Hz, 1 H, aromatic CH), 7.41 (m, 2 H, aromatic CH), 7.29 (m, 3 H, aromatic CH), 6.85 (d, J = 8.4 Hz, 1 H, aromatic CH), 3.96 and 3.93 (s each, 3:3 H, 2×OCH3), 3.67 (s, 3 H, NCH_3), 2.42 (s, 3 H, CH3). $^{13}C\{^1H\}$ (100 MHz, $CDCl_3$) δ 195.6 and 194.6 (Cq each, C=O), 154.0, 149.4, 142.7, 142.5, 140.8, 135.6, 130.8, 127.7, 124.5, 122.7 and 122.5 (Cq each), 130.1, 129.2, 129.0, 127.1, 127.0, 125.0, 120.6, 120.5, 111.2, 110.0 and 109.5 (CH), 56.2 and 56.1 (2×OCH_3), 32.9 (NCH_3), 21.6 (CH_3). HRMS Calcd for $C_{30}H_{26}NO_4$ $[M+H]^+$: 464.1862; Found: 464.1859.

(1-(3,4-Dimethoxybenzoyl)-9-methyl-9H-carbazol-3-yl)(4-methoxyphenyl)methanone (3m): White solid, m.p.: 212-214 °C. ^{1}H NMR (400 MHz, $CDCl_3$) δ 8.70 (s, 1 H, aromatic CH), 8.14 (d, J = 7.8 Hz, 1 H, aromatic CH), 7.97 (s, 1 H, aromatic CH), 7.86 (d, J = 8.5 Hz, 2 H, aromatic CH), 7.69 (d, J = 1.5 Hz, 1 H, aromatic CH), 7.54 (t, J = 7.3 Hz, 1 H, aromatic CH), 7.44 (d, J = 8.2 Hz, 1 H, aromatic CH), 7.39 (m, 1 H, aromatic CH), 7.32 (t, J = 7.3 Hz, 1 H, aromatic CH), 6.97 (d, J = 8.5 Hz, 2 H, aromatic CH), 6.85 (d, J = 8.4 Hz, 1 H, aromatic CH), 3.96, 3.94 and 3.87 (s each, 3:3:3 H, 3×OCH_3), 3.68 (s, 3 H, NCH_3). $^{13}C\{^1H\}$ (100 MHz, $CDCl_3$) δ 194.8 (Cq, 2×C=O), 163.0, 154.1, 149.5, 142.6, 140.7, 130.9, 130.9, 128.1, 124.5, 122.8 and 122.5 (Cq each), 132.4, 129.2, 127.2, 127.1, 124.9, 120.6, 120.6, 113.7, 111.3, 110.1 and 109.5 (CH), 56.3, 56.2 and 55.6 (3×OCH_3), 32.9 (NCH_3). HRMS Calcd for $C_{30}H_{26}NO_5$ $[M+H]^+$: 480.1811; Found: 480.1806.

(9-Methyl-9H-carbazole-1,3-diyl)bis((3,4-dimethoxyphenyl)methanone) (3n): White solid, m.p.: 196-198 °C. ^{1}H NMR (400 MHz, $CDCl_3$) δ 8.69 (s, 1 H, aromatic CH), 8.11 (d, J = 7.7 Hz, 1 H, aromatic CH), 7.96 (s, 1 H, aromatic CH), 7.67 (d, J = 1.3 Hz, 1 H, aromatic CH), 7.52-7.45 (m, 2 H, aromatic CH), 7.43-7.34 (m, 3 H, aromatic CH), 7.29 (d, J = 7.5 Hz, 1 H, aromatic CH), 6.87 (d, J = 8.3 Hz, 1 H, aromatic CH), 6.82 (d, J = 8.4 Hz, 1 H, aromatic CH), 3.93, 3.91 and 3.90 (s each, 3:3:6 H, 4×OCH_3), 3.64 (s, 3 H, NCH_3). $^{13}C\{^1H\}$ (100 MHz, $CDCl_3$) δ 194.7 and 194.6 (Cq each, C=O), 154.0, 152.6, 149.3, 148.9, 130.9, 130.7, 142.4, 140.6, 127.9, 124.4, 122.6 and 122.4 (Cq each), 129.0, 127.0, 126.9, 124.9, 124.8, 120.5, 120.4, 112.3, 111.2, 110.0, 109.8 and 109.4 (CH), 56.1, 56.03, 56.01 and 56.00 (4×OCH_3), 32.8 (NCH_3). HRMS Calcd for $C_{31}H_{28}NO_6$ $[M+H]^+$: 510.1911; Found: 510.1909.

(1-(3,4-Dimethoxybenzoyl)-9-methyl-9H-carbazol-3-yl)(4-fluorophenyl)methanone (3o): White solid, m.p.: 205-207 °C. ^{1}H NMR (400 MHz, $CDCl_3$) δ 8.69 (d, J = 1.4 Hz, 1 H, aromatic CH), 8.14 (d, J = 7.8 Hz, 1 H, aromatic CH), 7.97 (d, J = 1.4 Hz, 1 H, aromatic CH), 7.86 (m, 2 H, aromatic CH), 7.70 (d, J = 1.7 Hz, 1 H, aromatic CH), 7.54 (t, J = 7.7 Hz, 1 H, aromatic CH), 7.44 (d, J = 8.2 Hz, 1 H, aromatic CH), 7.37 (m, 1 H, aromatic CH), 7.32 (t, J = 7.4 Hz, 1 H, aromatic CH), 7.16 (t, J = 8.6 Hz, 2 H, aromatic CH), 6.85 (d, J = 8.4 Hz, 1 H, aromatic CH), 3.96 and 3.94 (s each, 3:3 H, 2×OCH_3), 3.67 (s, 3 H, NCH_3). ^{13}C NMR (101 MHz, $CDCl_3$) δ 194.6 and 194.4 (Cq each, C=O), 165.2 (Cq, d, J = 251.8 Hz, i-C of C6H4F), 154.2, 149.5, 142.5, 140.9, 134.6 (Cq, d, J = 3.0 Hz, p-C of C6H4F), 130.8, 127.3, 124.6, 122.70 and 122.67, (Cq each), 132.4 (CH, d, J = 9.0 Hz, m-C of C_6H_4F), 129.1, 127.3, 127.1, 125.0, 120.8, 120.6, 115.5 (CH, d, J = 22.0 Hz, o-C of C_6H_4F), 111.2, 110.1 and 109.6 (CH), 56.24 and 56.16 (2×OCH_3), 32.9 (NCH_3). HRMS Calcd for $C_{29}H_{23}FNO_4$ $[M+H]^+$: 468.1611; Found: 468.1610.

(3-(4-Chlorobenzoyl)-9-methyl-9H-carbazol-1-yl)(3,4-dimethoxyphenyl)methanone (3p): White solid, m.p.: 202-204 °C. ^{1}H NMR (400 MHz, $CDCl_3$) δ 8.69 (d, J = 1.5 Hz, 1 H, aromatic CH), 8.15 (d, J = 7.8 Hz, 1 H, aromatic CH), 7.97 (d, J = 1.6 Hz, 1 H, aromatic CH), 7.78 (d, J = 8.4 Hz, 2 H, aromatic CH), 7.70 (d, J = 1.8 Hz, 1 H, aromatic CH), 7.56 (t, J = 7.6 Hz, 1 H, aromatic CH), 7.46 (m, 3 H, aromatic CH), 7.39-7.32 (m, 2 H, aromatic CH), 6.85 (d, J = 8.4 Hz, 1 H, aromatic CH), 3.97 and 3.95 (s each, 3:3 H, 2×OCH_3), 3.68 (s, 3 H, NCH_3). $^{13}C\{^1H\}$ (100 MHz, $CDCl_3$) δ 194.70 and 194.66 (Cq each, C=O), 154.2, 149.6, 142.6, 141.1, 138.5,

136.8, 130.8, 127.2, 124.7, 122.8 and 122.7 (Cq each), 131.4, 129.1, 128.7, 127.4, 127.1, 125.1, 120.9, 120.6, 111.3, 110.1 and 109.6 (CH), 56.3 and 56.2 (2×OCH_3), 33.0 (NCH_3). HRMS Calcd for $C_{29}H_{23}ClNO_4$ $[M+H]^+$: 484.1310; Found: 484.1315. Single crystal of 3p were obtained by the slow evaporation of its EtOH/DCM (1:1 v) solution.

4-(1-(3,4-Dimethoxybenzoyl)-9-methyl-9H-carbazole-3-carbonyl)benzonitrile(3q): White solid, m.p.: 217-219 ºC. ^{1}H NMR (400 MHz, $CDCl_3$) δ 8.64 (d, J = 1.5 Hz, 1 H, aromatic CH), 8.11 (d, J = 7.8 Hz, 1 H, aromatic CH), 7.95 (d, J = 1.5 Hz, 1 H, aromatic CH), 7.86 (d, J = 8.3 Hz, 2 H, aromatic CH), 7.75 (d, J = 8.3 Hz, 2 H, aromatic CH), 7.68 (d, J = 1.8 Hz, 1 H, aromatic CH), 7.54 (t, J = 7.7 Hz, 1 H, aromatic CH), 7.43 (d, J = 8.3 Hz, 1 H, aromatic CH), 7.32 (m, 2 H, aromatic CH), 6.84 (d, J = 8.4 Hz, 1 H, aromatic CH), 3.95 and 3.93 (s each, 3:3 H, 2×OCH_3), 3.65 (s, 3 H, NCH_3). $^{13}C\{^1H\}$ (100 MHz, $CDCl_3$) δ 194.3 and 194.0 (Cq each, C=O), 154.2, 149.5, 142.5, 142.2, 141.2, 130.6, 124.7, 122.9, 122.5, 118.1 and 115.2 (Cq each), 132.2, 130.0, 128.9, 127.4, 127.0, 126.2, 125.0, 120.9, 120.5, 111.2, 110.0 and 109.6 (CH), 56.2 and 56.1 (2×OCH_3), 32.9 (NCH_3). HRMS Calcd for $C_{30}H_{23}N_2O_4$ $[M+H]^+$: 475.1658; Found: 475.1658. Single crystal of 3q were obtained by the slow evaporation of its EtOH/DCM (1:1 v) solution.

(3-(2-Naphthoyl)-9-methyl-9H-carbazol-1-yl)(3,4-dimethoxyphenyl)methanone (3r): White solid, m.p.: 177-179 ºC. ^{1}H NMR (400 MHz, $CDCl_3$) δ 8.81 (d, J = 1.5 Hz, 1 H, aromatic CH), 8.31 (s, 1 H, aromatic CH), 8.10 (m, 2 H, aromatic CH), 7.93 (m, 4 H, aromatic CH), 7.71 (d, J = 1.8 Hz, 1 H, aromatic CH), 7.55 (m, 3 H, aromatic CH), 7.43 (d, J = 7.9 Hz, 2 H, aromatic CH), 7.30 (t, J = 7.4 Hz, 1 H, aromatic CH), 6.84 (d, J = 8.4 Hz, 1 H, aromatic CH), 3.95 and 3.91 (s each, 3:3 H, 2×OCH_3), 3.69 (s, 3 H, NCH_3). $^{13}C\{^1H\}$ (100 MHz, $CDCl_3$) δ 195.8 and 194.5 (Cq each, C=O), 154.0, 149.4, 142.5, 140.9, 135.6, 135.0, 132.2, 127.6, 124.6, 122.7 and 122.5 (Cq each), 131.3, 130.7, 129.5, 129.3, 128.2, 128.1, 127.8, 127.1, 127.0, 126.7, 126.0, 125.2, 120.6, 120.5, 111.2, 110.0 and 109.5 (CH), 56.1 and 56.0 (2×OCH3), 32.9 (NCH_3). HRMS Calcd for $C_{33}H_{26}NO_4$ $[M+H]^+$: 500.1862; Found: 500.1861.

(1-(3,4-Dimethoxybenzoyl)-9-methyl-9H-carbazol-3-yl)(4-(1,2,2-triphenylvinyl)phenyl)methanone (3s): White solid, m.p.: 131-133 ºC. ^{1}H NMR (400 MHz, $CDCl_3$) δ 8.70 (d, J = 1.5 Hz, 1 H, aromatic CH), 8.15 (d, J = 7.7 Hz, 1 H, aromatic CH), 8.01 (d, J = 1.5 Hz, 1 H, aromatic CH), 7.75 (d, J = 1.7 Hz, 1 H, aromatic CH), 7.63 (d, J = 8.2 Hz, 2 H, aromatic CH), 7.55 (t, J = 7.5 Hz, 1 H, aromatic CH), 7.45 (d, J = 8.3 Hz, 1 H, aromatic CH), 7.41 (m, 1 H, aromatic CH), 7.36 (t, J = 7.4 Hz, 1 H, aromatic CH), 7.20 (d, J = 8.2 Hz, 2 H, aromatic CH), 7.17-7.07 (m, 16 H, aromatic CH), 6.87 (d, J = 8.5 Hz, 1 H, aromatic CH), 3.99 and 3.95 (s each, 3:3 H, 2×OCH_3), 3.68 (s, 3 H, NCH_3). $^{13}C\{^1H\}$ (100 MHz, $CDCl_3$) δ 195.4 and 194.6 (Cq each, C=O), 154.0, 149.4, 147.9, 143.0, 142.5, 142.5, 140.8, 140.0, 136.0, 130.8, 127.6, 125.1, 124.4, 122.7 and 122.5 (Cq each), 143.2, 131.4, 131.3, 131.3, 129.5, 129.2, 127.9, 127.8, 127.7, 127.1, 127.0, 126.9, 126.8, 120.6, 120.5, 111.2, 110.0 and 109.5 (CH), 56.2 and 56.1 (2×OCH_3), 32.9 (NCH_3). HRMS Calcd for $C_{49}H_{38}NO_4$ $[M+H]^+$: 704.2801; Found: 704.2800.

(1-(3,4-Dimethoxybenzoyl)-9-methyl-9H-carbazol-3-yl)(thiophen-2-yl)methanone (3t): White solid, m.p.: 151-153 ºC. ^{1}H NMR (400 MHz, $CDCl_3$) δ 8.80 (s, 1 H, aromatic CH), 8.15 (d, J = 7.7 Hz, 1 H, aromatic CH), 8.07 (d, J = 1.5 Hz, 1 H, aromatic CH), 7.70 (m, 2 H, aromatic CH and thienyl CH), 7.67 (d, J = 4.6 Hz, 1 H, thienyl CH), 7.51 (d, J = 7.4 Hz, 1 H, aromatic CH), 7.40 (m, 2 H, aromatic CH), 7.31 (t, J = 7.4 Hz, 1 H, aromatic CH), 7.13 (m, 1 H, thienyl CH), 6.85 (d, J = 8.4 Hz, 1 H, aromatic CH), 3.95 and 3.92 (s each, 3:3 H, 2×OCH3), 3.65 (s, 3 H, NCH_3). $^{13}C\{^1H\}$ (100 MHz, $CDCl_3$) δ 194.5 and 187.0 (Cq each, C=O), 154.0, 149.4, 143.8, 142.4, 140.8, 134.2, 130.7, 127.9 and 124.6 (Cq each), 133.5, 128.5, 127.9, 127.2, 127.0, 124.1, 122.6, 122.6, 120.6, 120.5, 111.2, 110.0 and 109.5 (CH), 56.2 and 56.1 (2×OCH_3) , 32.9 (NCH_3). HRMS Calcd for $C_{27}H_{22}NO_4S$ $[M+H]^+$: 456.1270; Found: 456.1270.

(1-(3,4-Dimethoxybenzoyl)-9-methyl-9H-carbazol-3-yl)(furan-2-yl)methanone (3u): White solid, m.p.: 161-163 ºC. ^{1}H NMR (400 MHz, $CDCl_3$) δ 8.94 (d, J = 1.5 Hz, 1 H, aromatic CH), 8.20 (d, J = 1.5 Hz, 1 H, aromatic CH), 8.16 (d, J = 7.7 Hz, 1 H, aromatic CH), 7.69 (d, J = 1.8 Hz, 1 H, aromatic CH), 7.67 (d, J = 0.8 Hz, 1 H, aromatic CH), 7.50 (t, J = 7.7 Hz, 1 H, aromatic CH), 7.40 (m, 2 H, aromatic CH), 7.29 (m, 2 H, aromatic CH and furyl CH), 6.84 (m, 1 H, furyl CH), 6.57 (dd, J = 3.5, 1.6 Hz, 1 H, furyl CH), 3.94 and 3.91 (s each, 3:3 H, 2×OCH_3), 3.64 (s, 3 H, NCH_3). $^{13}C\{^1H\}$ (100 MHz, $CDCl_3$) δ 194.5 and 181.2 (Cq each, C=O), 154.0, 152.8, 149.4, 146.6, 142.4, 140.9, 130.8, 127.0 and 124.7 (Cq each), 128.7, 127.1, 126.98, 124.3, 122.7, 122.6, 120.7, 120.5, 119.9, 112.2, 111.3, 110.1 and 109.5 (CH), 56.2 and 56.1 (2×OCH_3), 32.9 (NCH_3). HRMS Calcd for $C_{27}H_{22}NO_5$ $[M+H]^+$: 440.1498; Found: 440.1494.

(3-(Cyclopropanecarbonyl)-9-methyl-9H-carbazol-1-yl)(3,4-dimethoxyphenyl)methanone (3v): White solid, m.p.: 171-173 ºC. ^{1}H NMR (400 MHz, $CDCl_3$) δ 8.94 (d, J = 1.0 Hz, 1 H, aromatic CH), 8.20 (d, J = 6.2 Hz, 2 H, aromatic CH), 7.70 (d, J = 1.2 Hz, 1 H, aromatic CH), 7.54 (t, J = 7.7 Hz, 1 H, aromatic CH), 7.43 (d, J = 8.2 Hz, 1 H, aromatic CH), 7.36 (m, 2 H, aromatic CH), 6.86 (d, J = 8.4 Hz, 1 H, aromatic CH), 3.97 and 3.95 (s each, 3:3 H, 2×OCH_3), 3.65 (s, 3 H, NCH_3), 2.84-2.75 (m, 1 H, cyclopropyl CH), 1.31-1.24 (m, 2 H,

cyclopropyl CH), 1.06 (m, 2 H, cyclopropyl CH). $^{13}C\{^{1}H\}$ (100 MHz, $CDCl_3$) δ 199.4 and 194.9 (Cq each, C=O), 154.1, 149.5, 142.6, 141.1, 131.0, 128.4, 124.8, 122.9 and 122.6 (Cq each), 127.4, 127.2, 127.0, 122.9, 120.7, 120.5, 111.3, 110.2 and 109.6 (CH), 56.3 and 56.2 (2×OCH_3), 33.0 (NCH_3), 17.1 (cyclopropyl CH), 11.6 (cyclopropyl CH_2). HRMS Calcd for $C_{26}H_{24}NO_4$ $[M+H]^+$: 414.1705; Found: 414.1698.

(*E*)-3-(1-(3-oxo-3-phenylpropyl)-1*H*-indol-3-yl)-1-phenylprop-2-en-1-one: Yield 21%. ^{1}H NMR (400 MHz, $CDCl_3$) δ 8.07 (d, *J* = 15.5 Hz, 2 H, C*H*=C*H*COPh), 8.02 (m, 2 H, aromatic CH), 7.90 (m, 2 H, aromatic CH), 7.65 (s, 1 H, 2-H of indolyl), 7.52 (m, 5 H, aromatic CH), 7.44 (t, 3 H, aromatic CH), 7.33 (m, 2 H, aromatic CH), 4.66 (t, 2 H, NCH_2CH_2COPh), 3.52 (t, 2 H, NCH_2CH_2COPh). $^{13}C\{^{1}H\}$NMR (100 MHz, $CDCl_3$) δ 197.1 and 190.8 (Cq, C=O), 139.2, 137.3, 136.2, 126.6, and 113.4 (Cq), 138.7, 134.2, 133.8, 132.2, 128.9, 128.6, 128.4, 128.1, 123.4, 121.8, 121.1, 117.5, and 110.2 (CH), 41.4 (NCH_2CH_2COPh), 38.4 (NCH_2CH_2COPh). HRMS Calcd for $C_{26}H_{21}NO_2$ $[M]^+$: 379.1572; Found: 379.1564.

2.2 Copies of NMR spectra

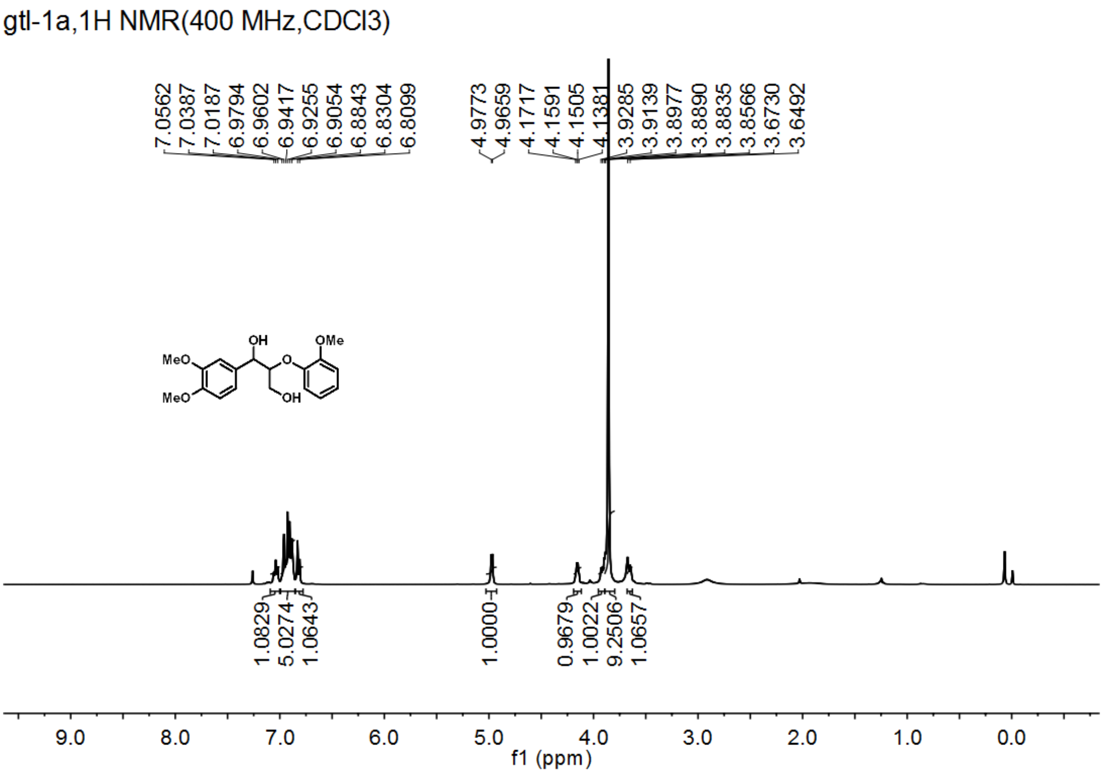

Supplementary Fig. 2. ^{1}H NMR spectra for **1a**.

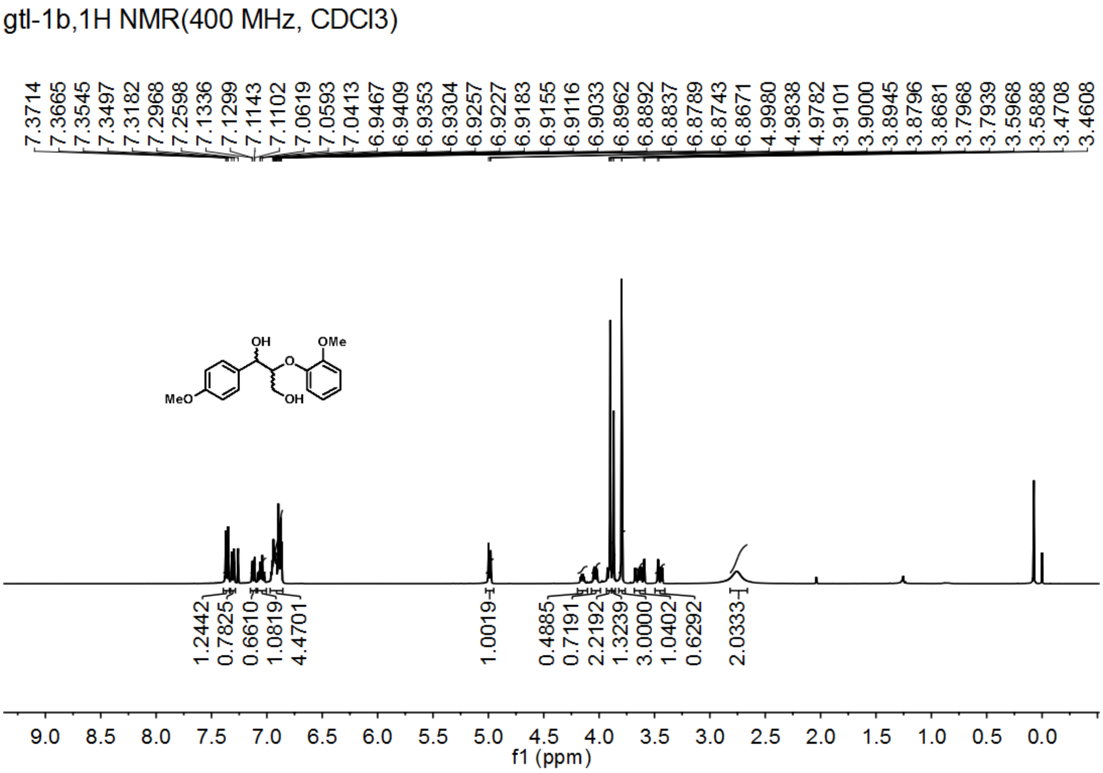

Supplementary Fig. 3. ^{1}H NMR spectra for **1b**.

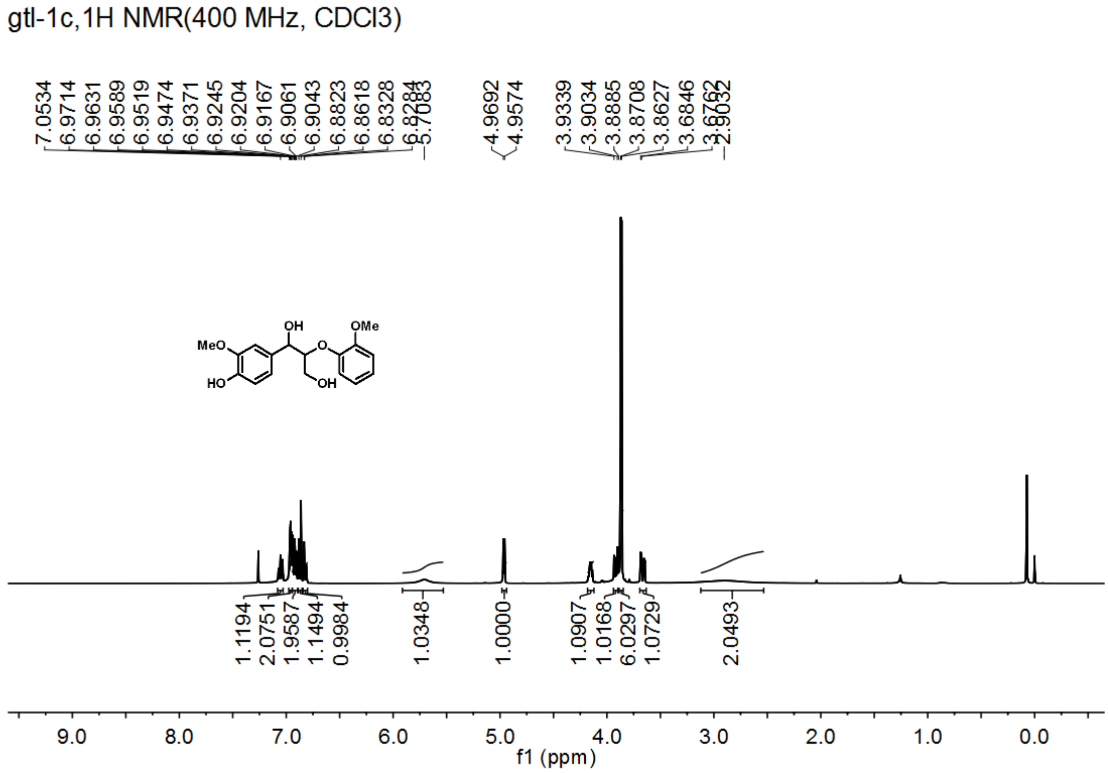

Supplementary Fig. 4. ^{1}H NMR spectra for **1c**.

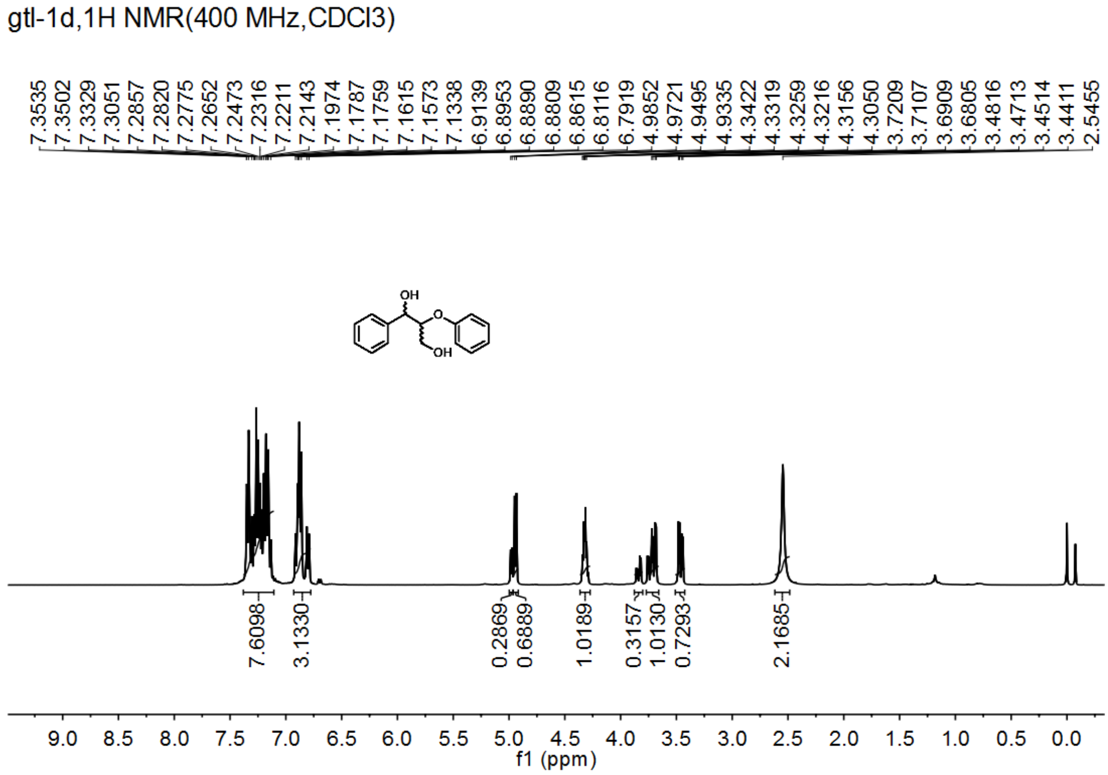

Supplementary Fig. 5. ^{1}H NMR spectra for **1d**.

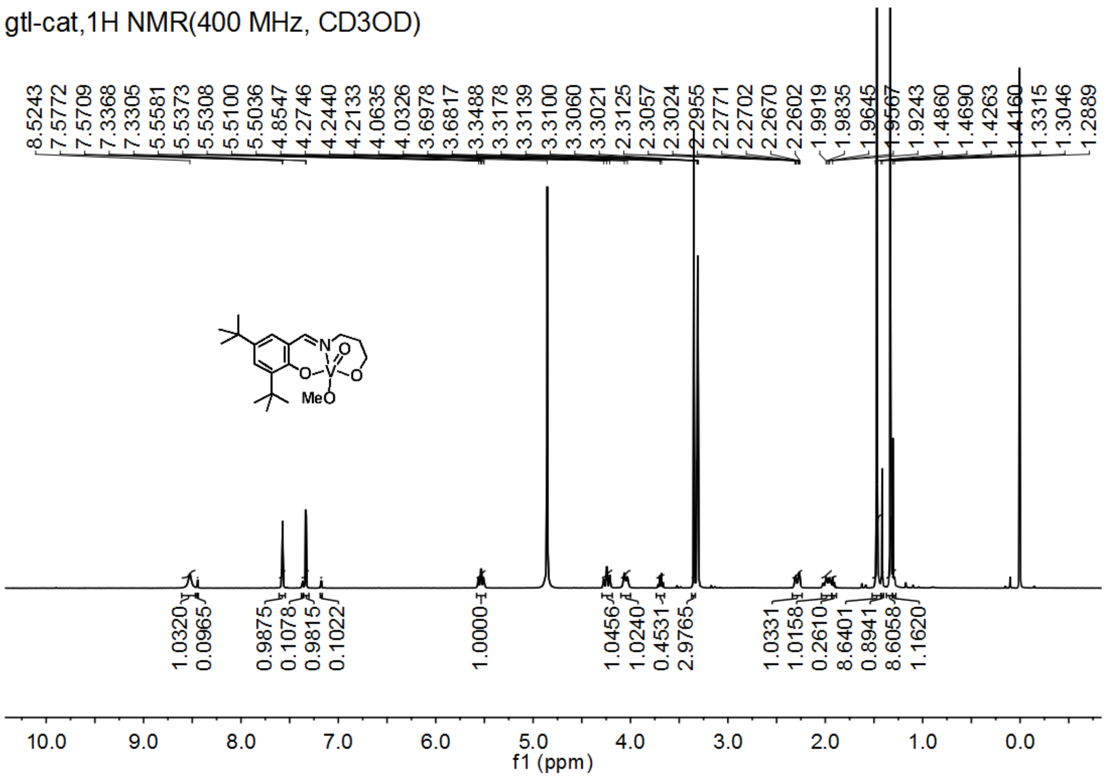

Supplementary Fig. 6. ^{1}H NMR spectra for V-complex catalyst.

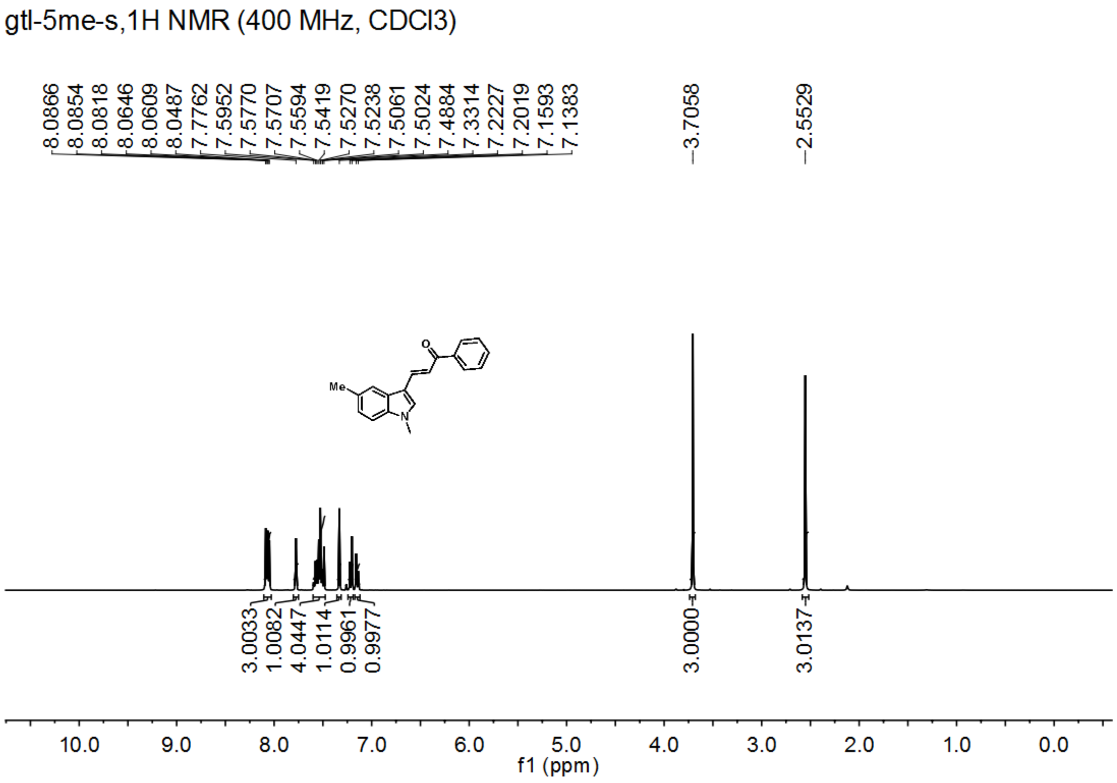

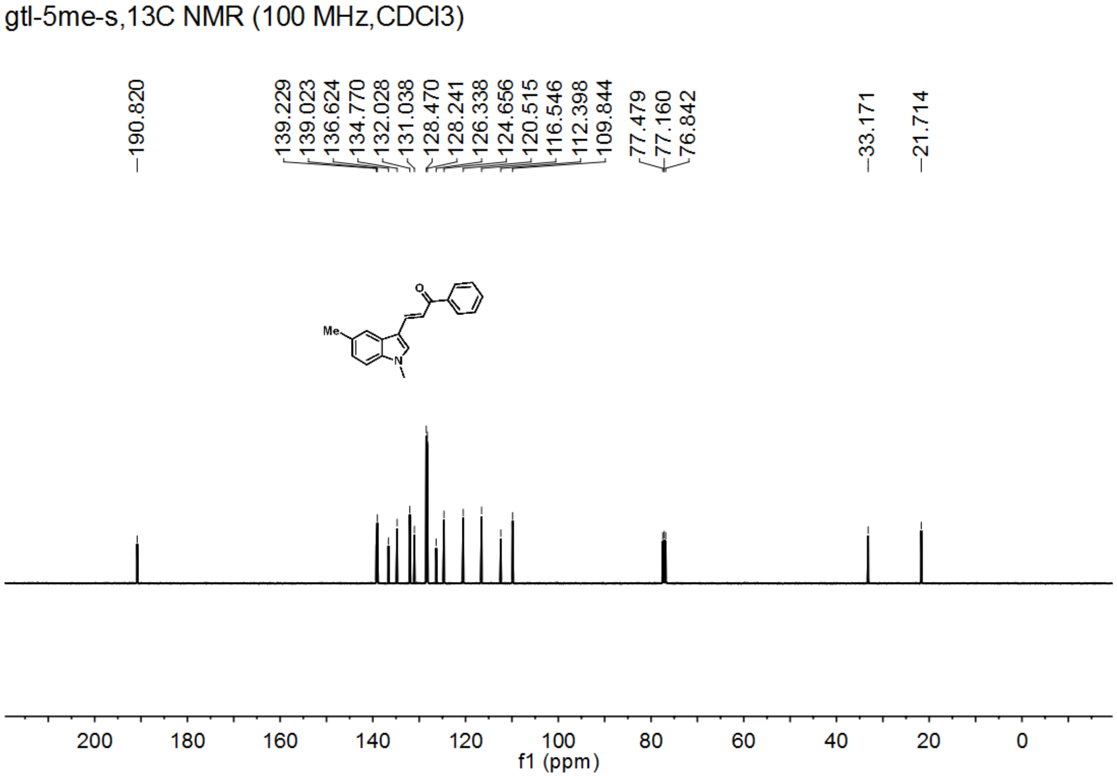

Supplementary Fig. 7. ^{1}H NMR and ^{13}C NMR spectra for **2c**.

gtl-6Me-s,1H NMR (400 MHz, CDCl3)

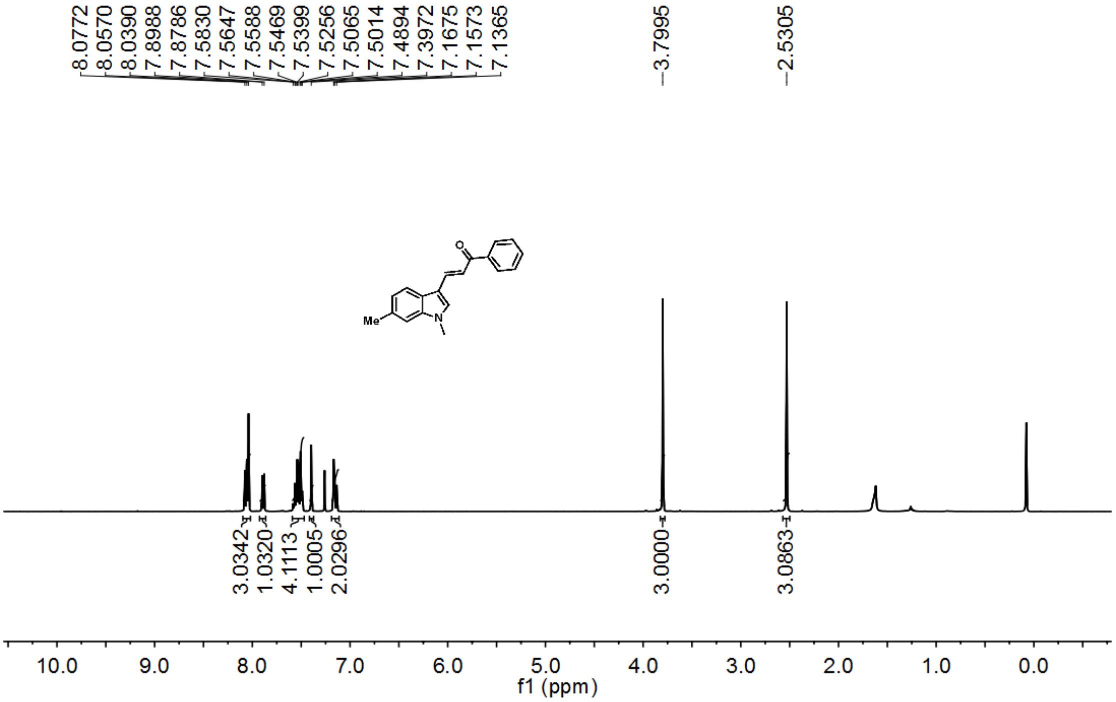

gtl-6Me-s,13C NMR (100 MHz, CDCl3)

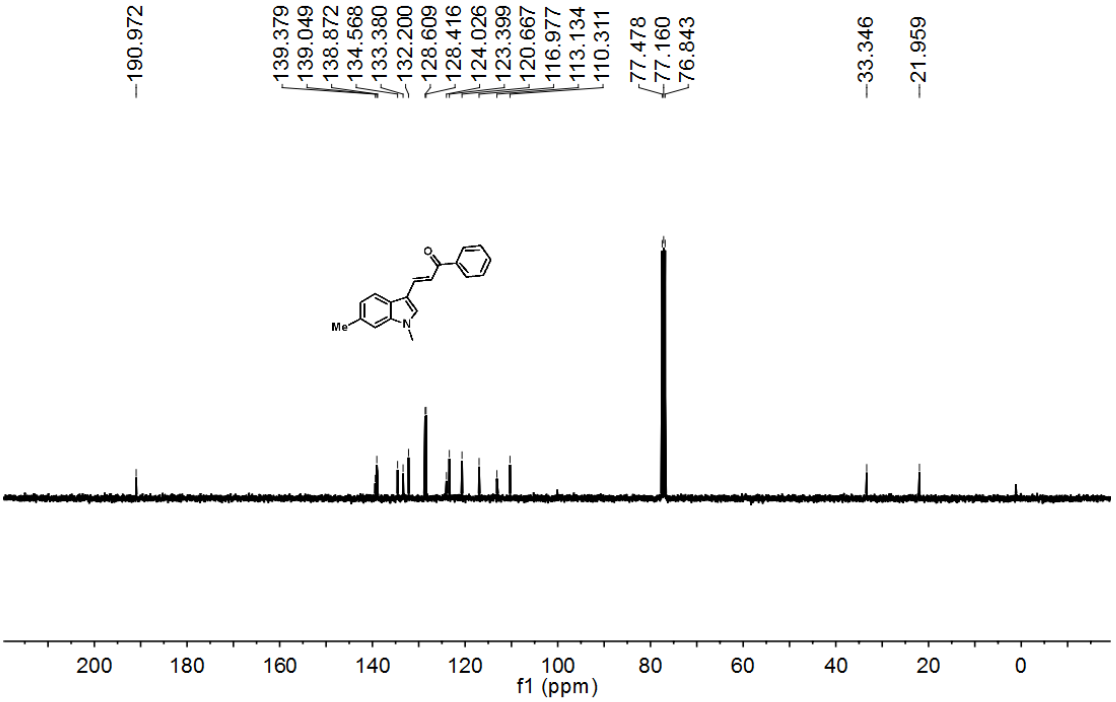

Supplementary Fig. 8. ^{1}H NMR and ^{13}C NMR spectra for **2d**.

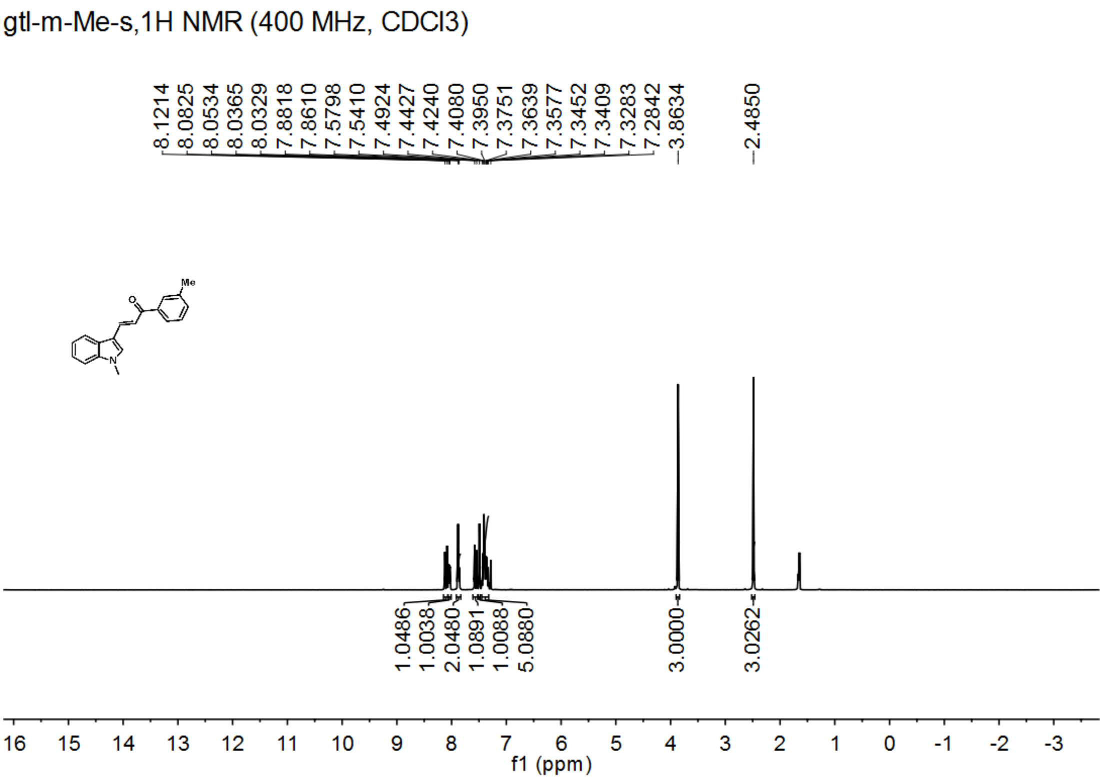

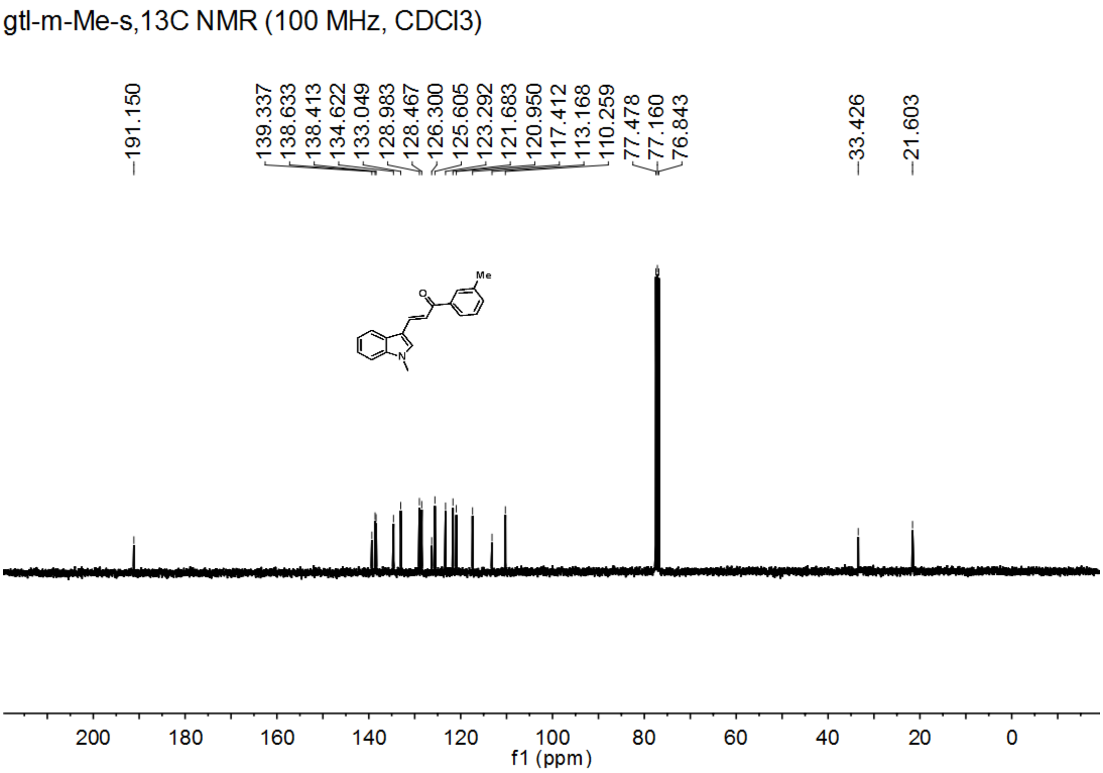

Supplementary Fig. 9. ^{1}H NMR and ^{13}C NMR spectra for **2h**.

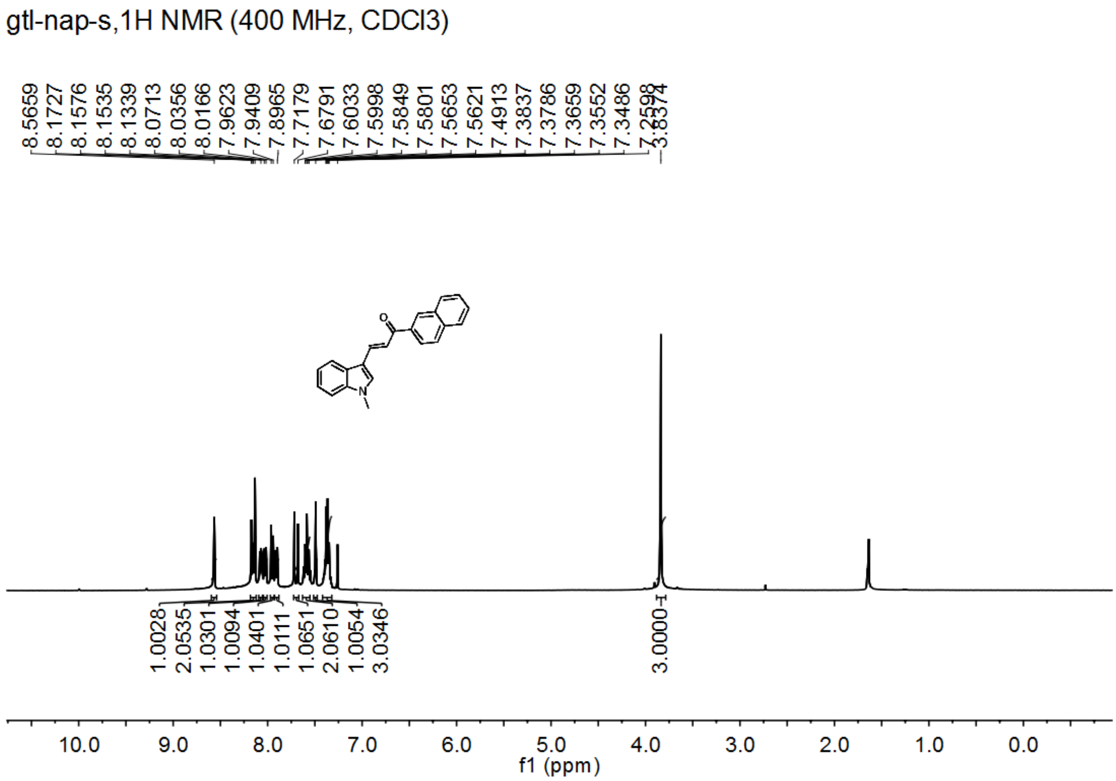

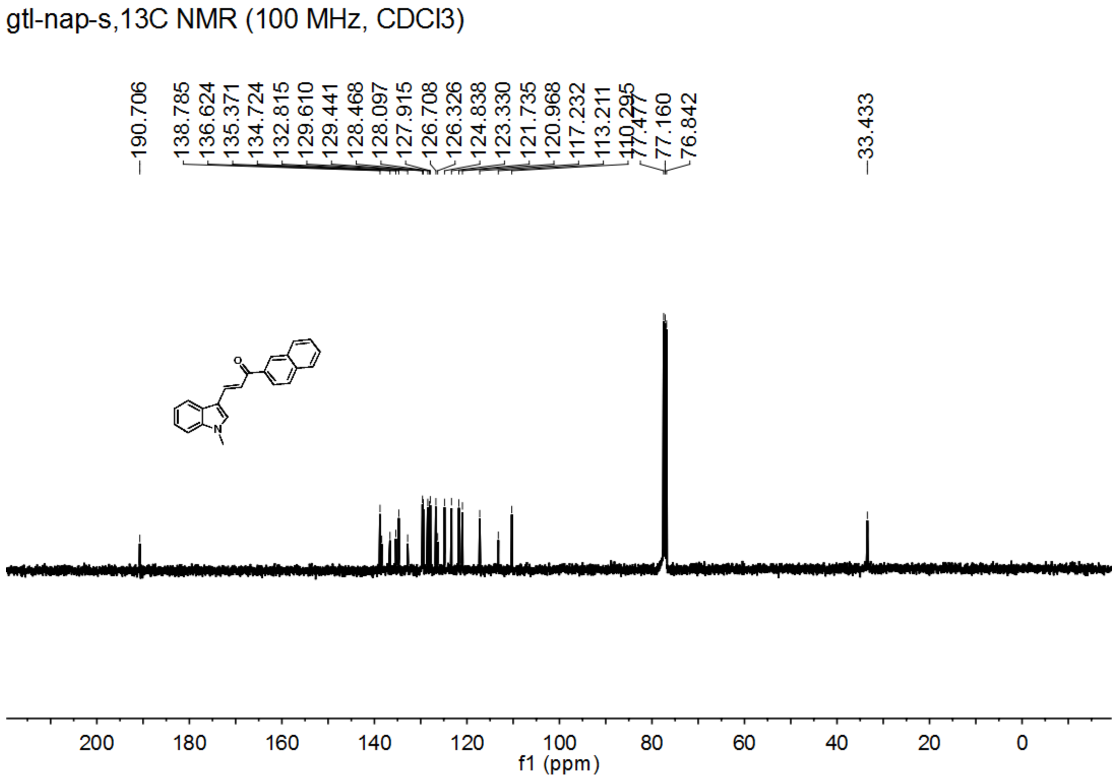

Supplementary Fig. 10. ^{1}H NMR and ^{13}C NMR spectra for **2o**.

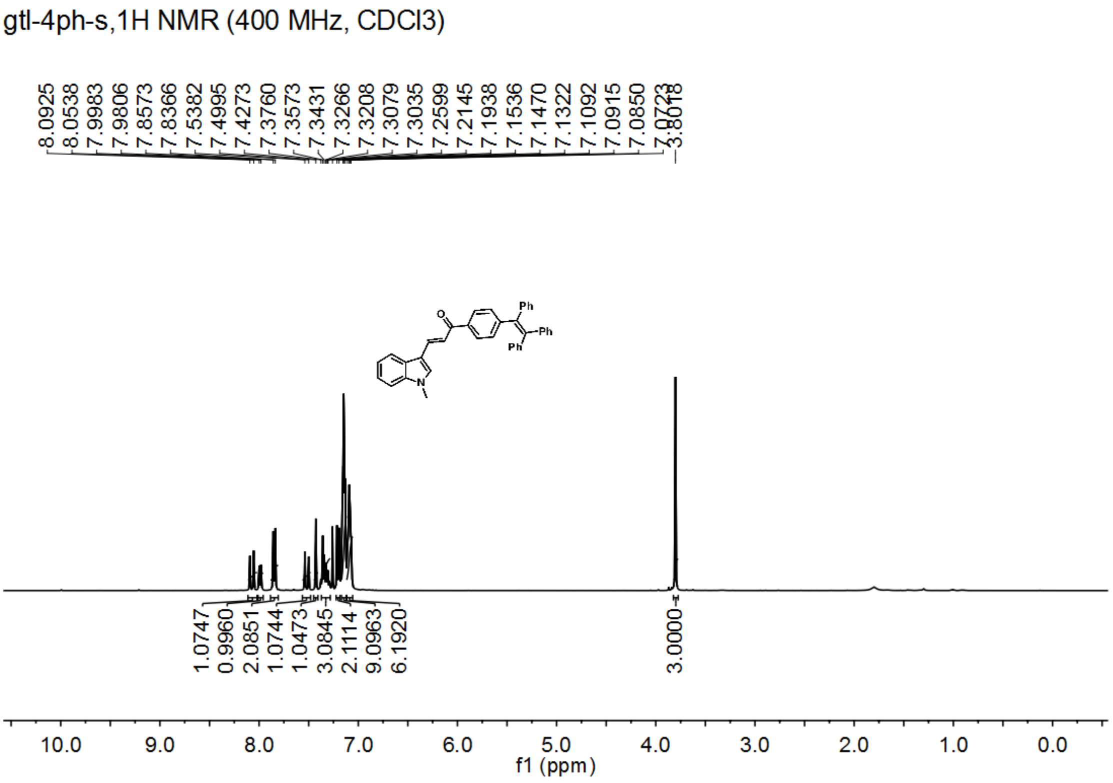

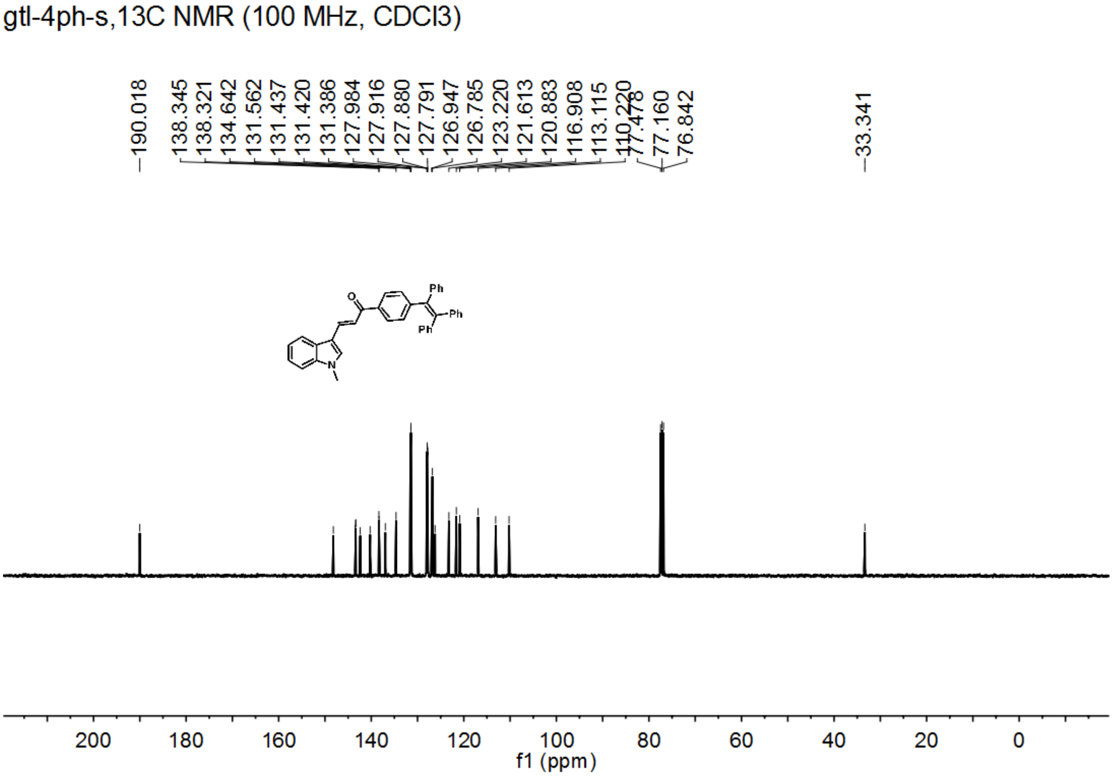

Supplementary Fig. 11. ^{1}H NMR and ^{13}C NMR spectra for **2p**.

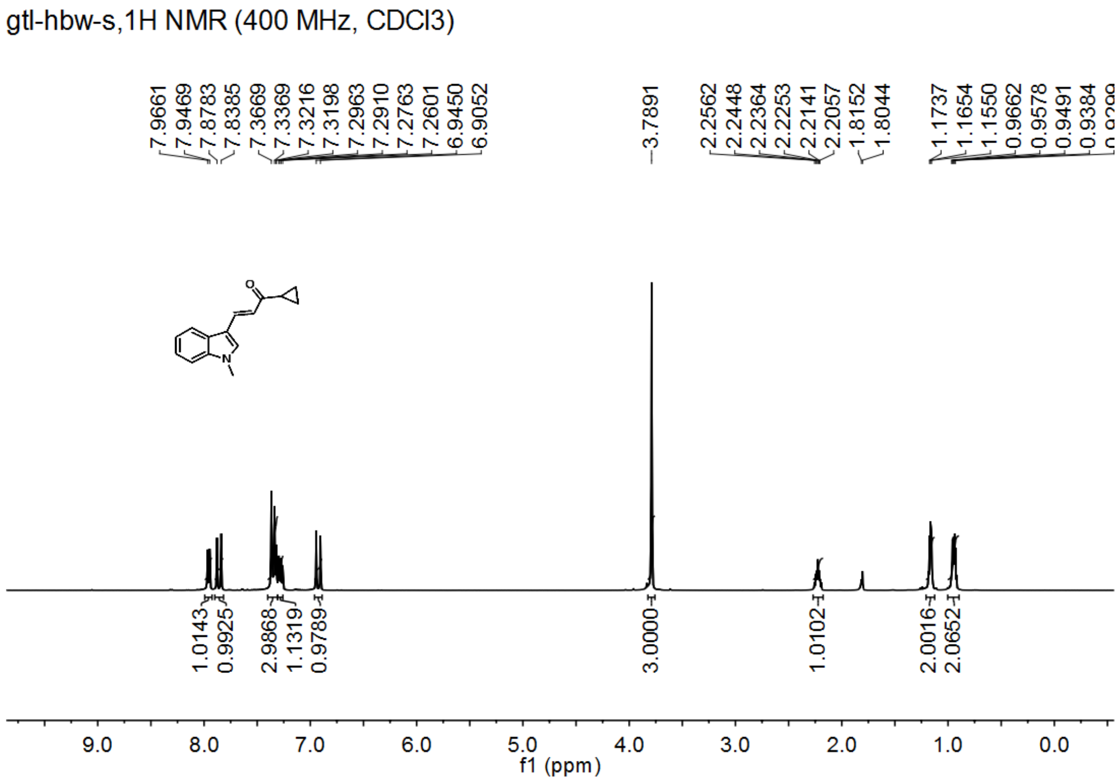

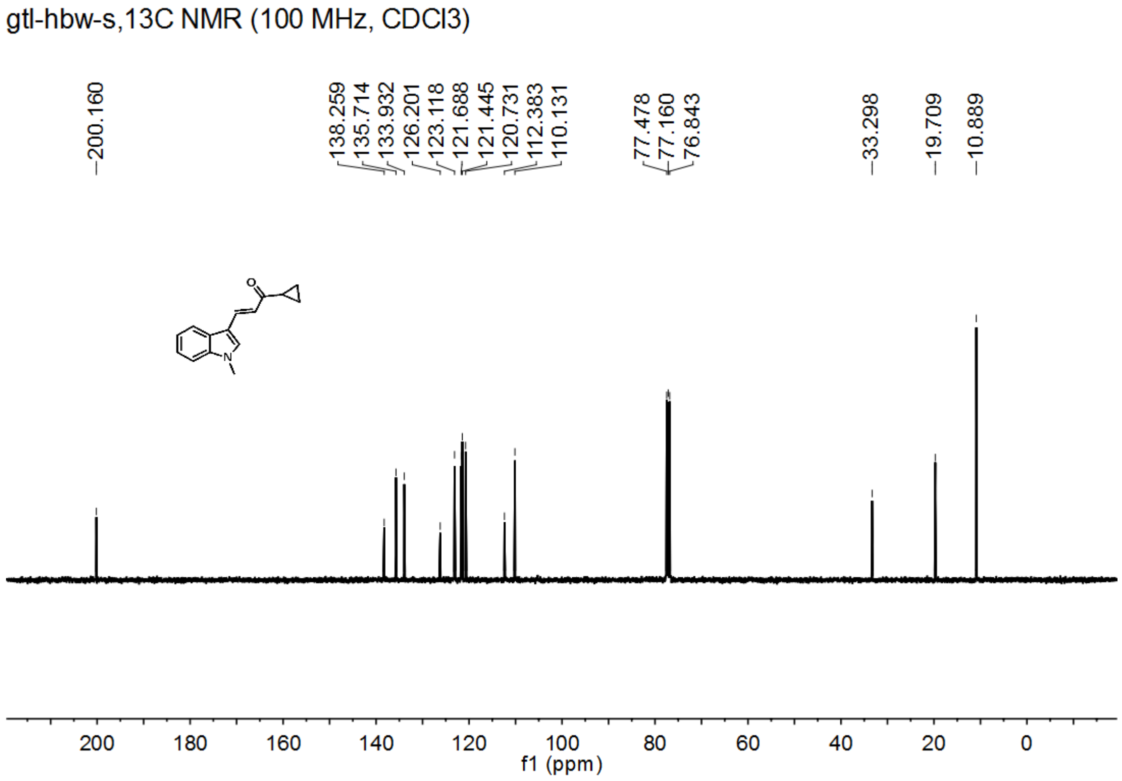

Supplementary Fig. 12. ^{1}H NMR and ^{13}C NMR spectra for **2s**.

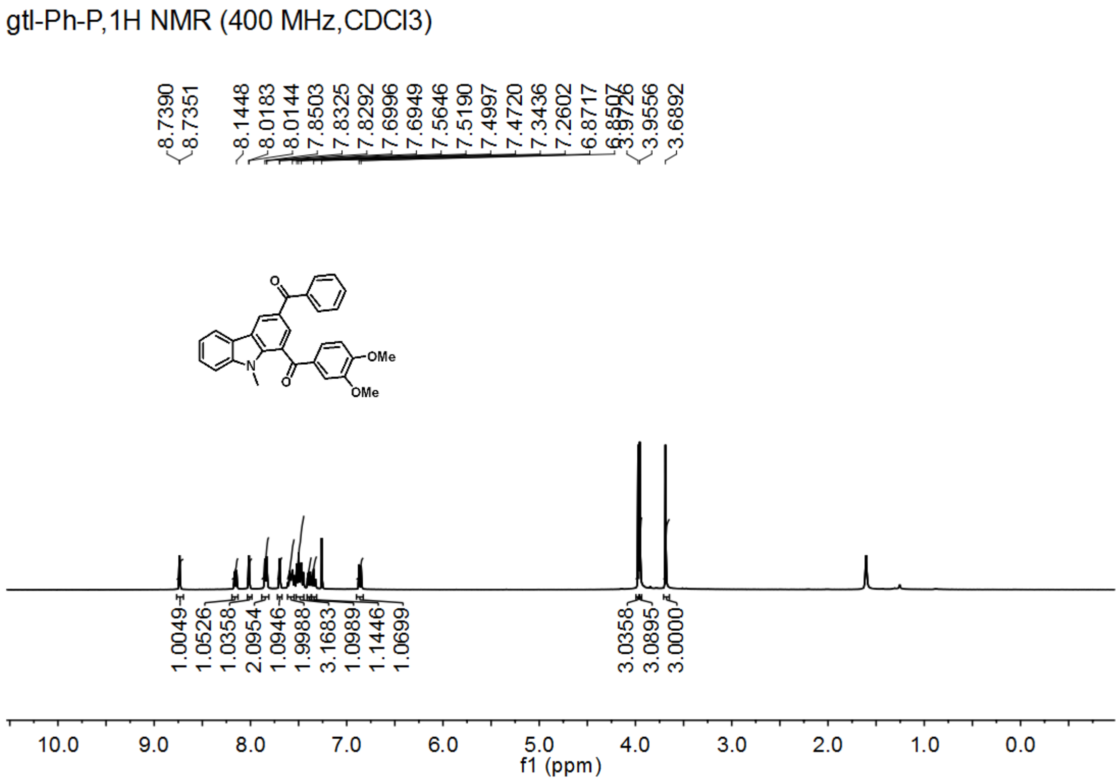

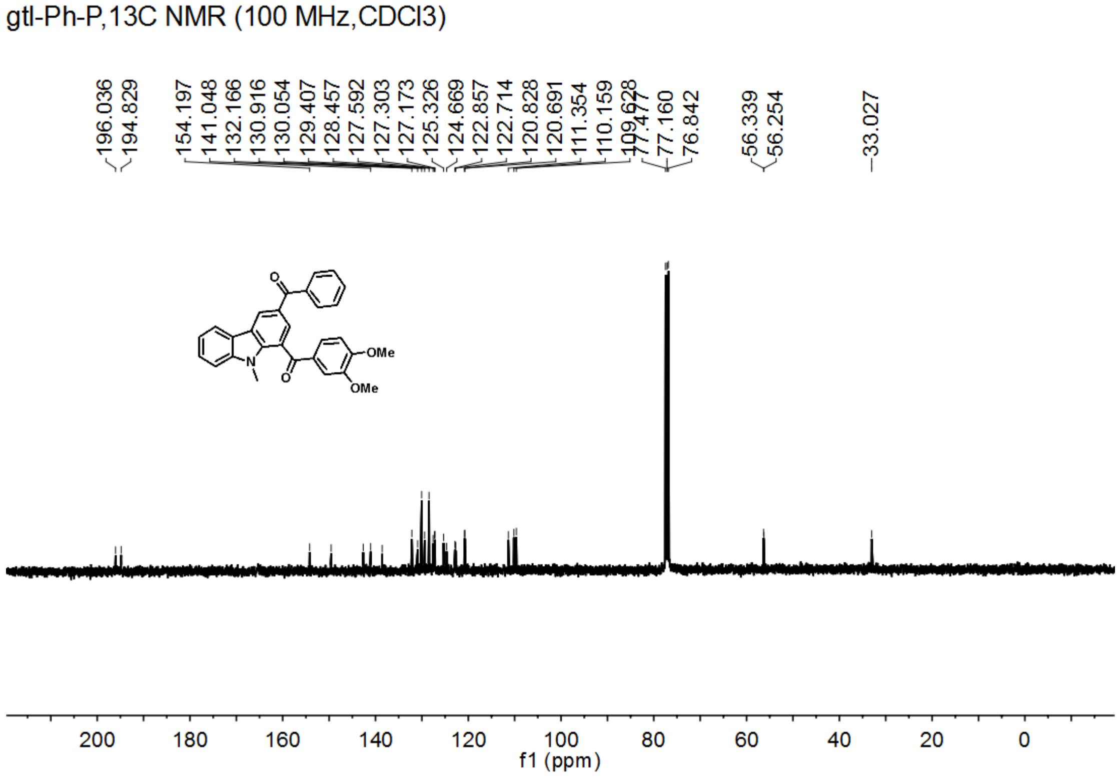

Supplementary Fig. 13. ^{1}H NMR and ^{13}C NMR spectra for **3a**.

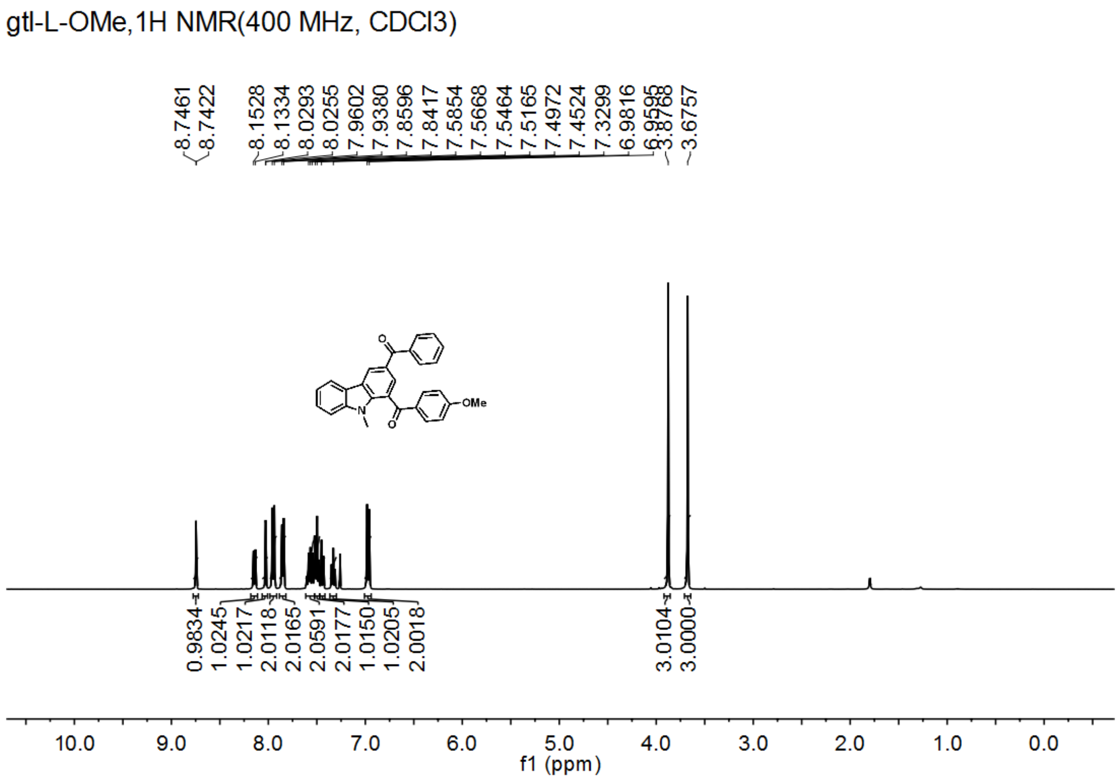

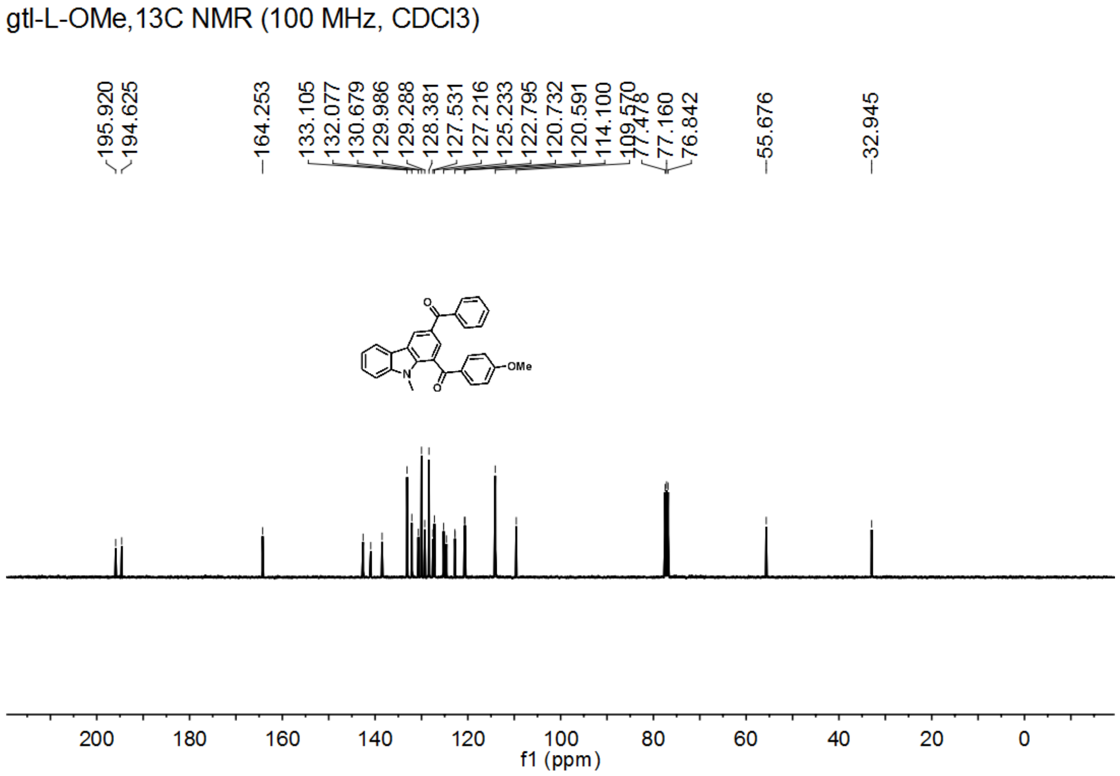

Supplementary Fig. 14. ^{1}H NMR and ^{13}C NMR spectra for **3b**.

gtl-OH,1H NMR (400 MHz, CDCL3)

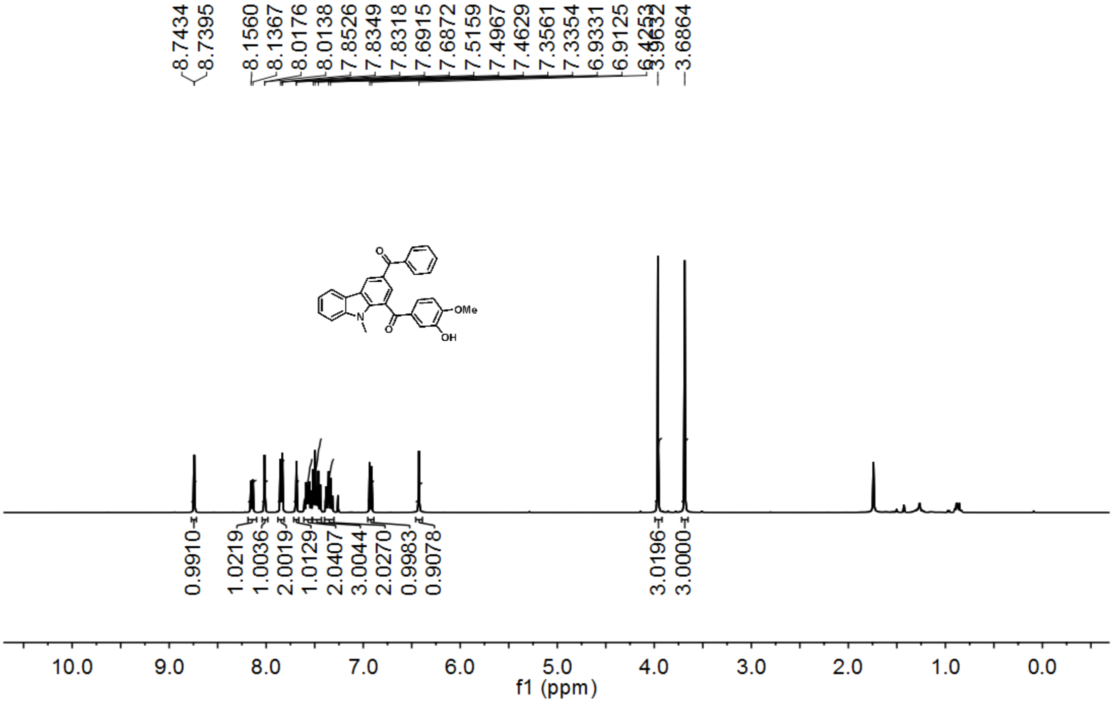

gtl-OH,13C NMR (100 MHz, CDCl3)

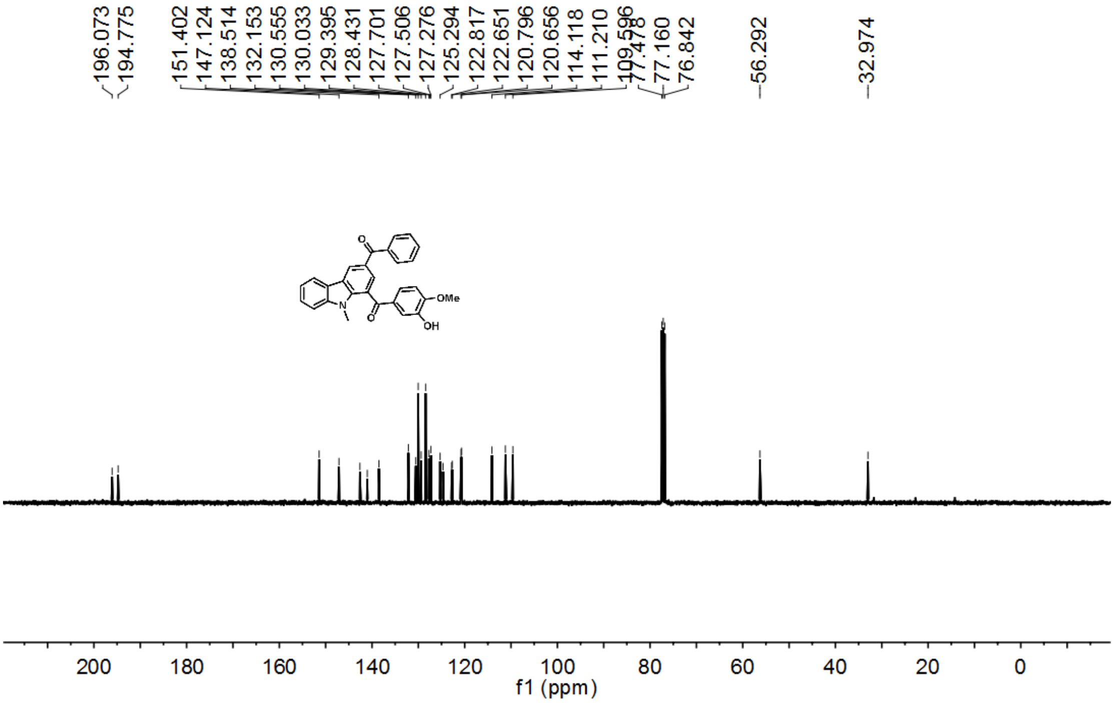

Supplementary Fig. 15. ^{1}H NMR and ^{13}C NMR spectra for **3c**.

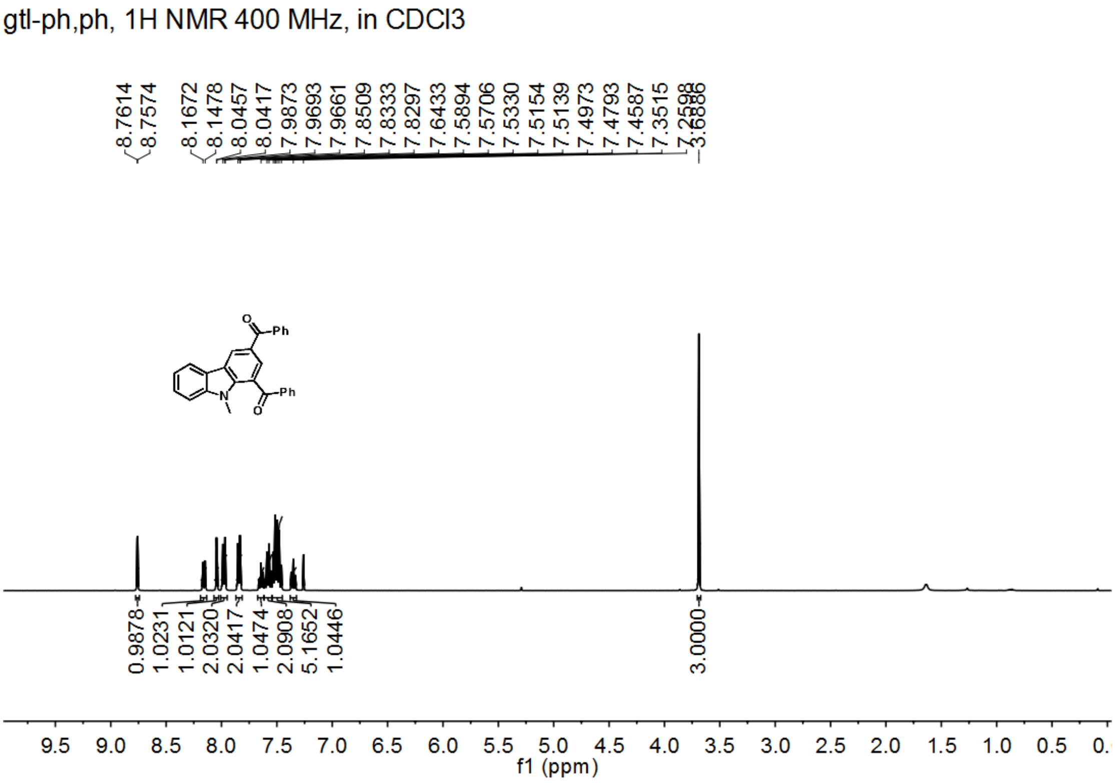

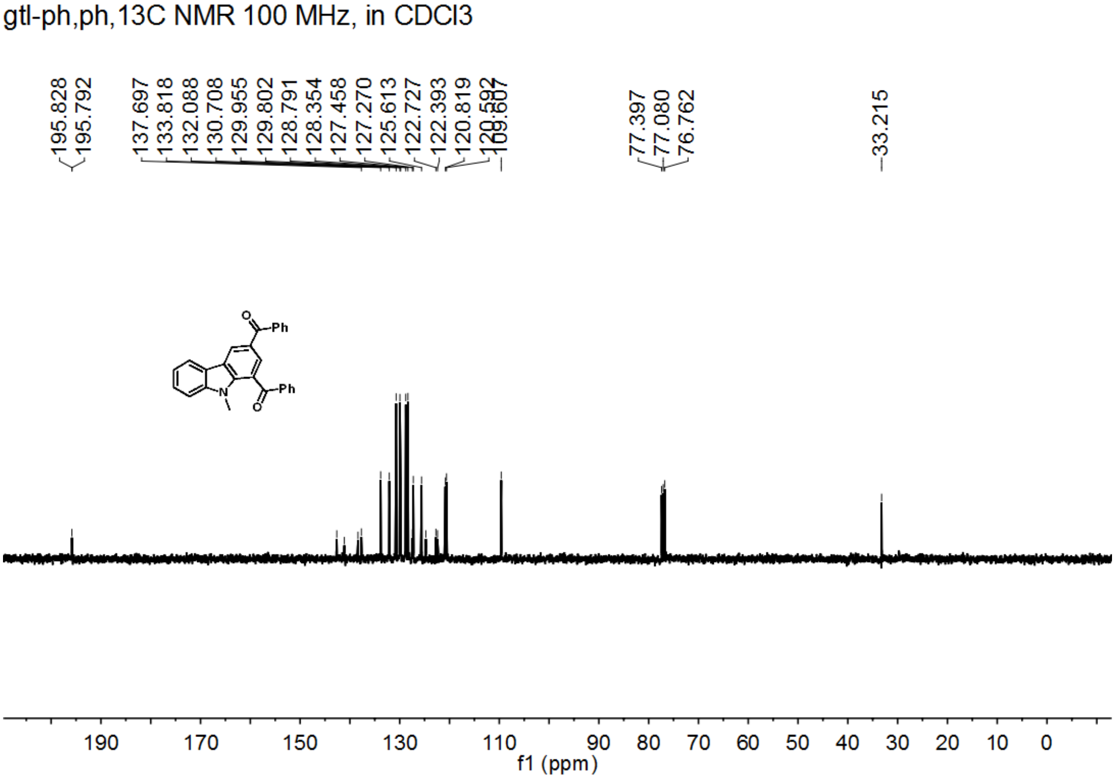

Supplementary Fig. 16. ^{1}H NMR and ^{13}C NMR spectra for **3d**.

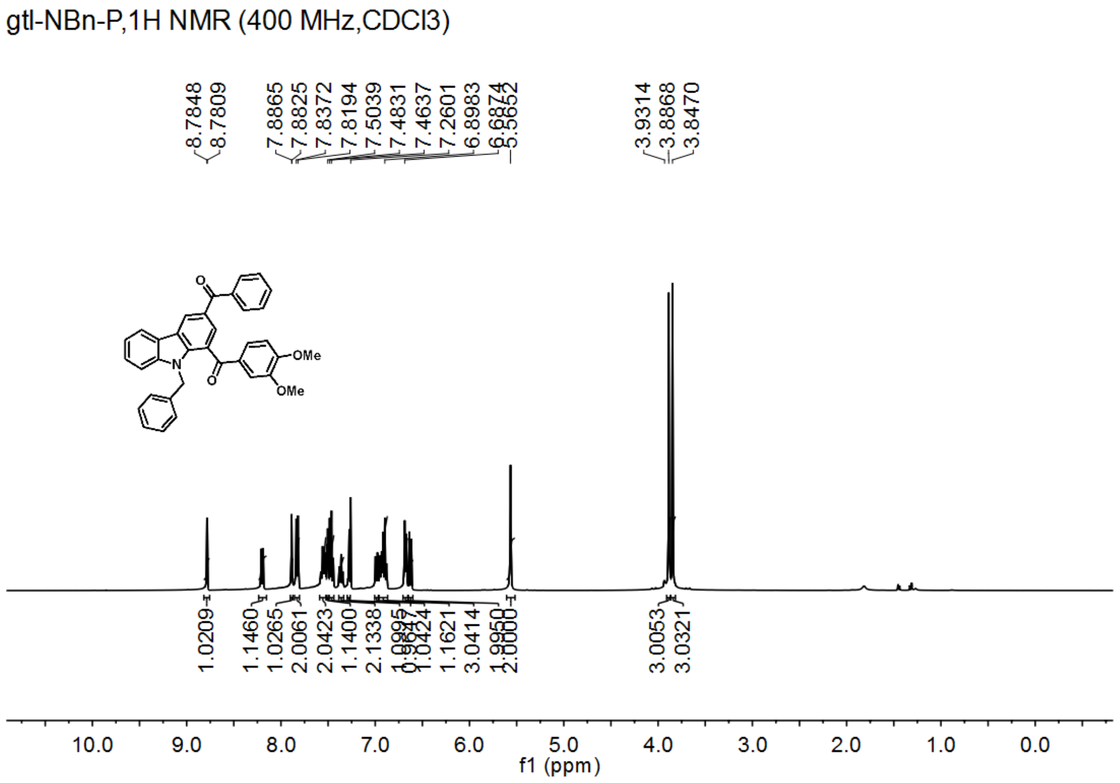

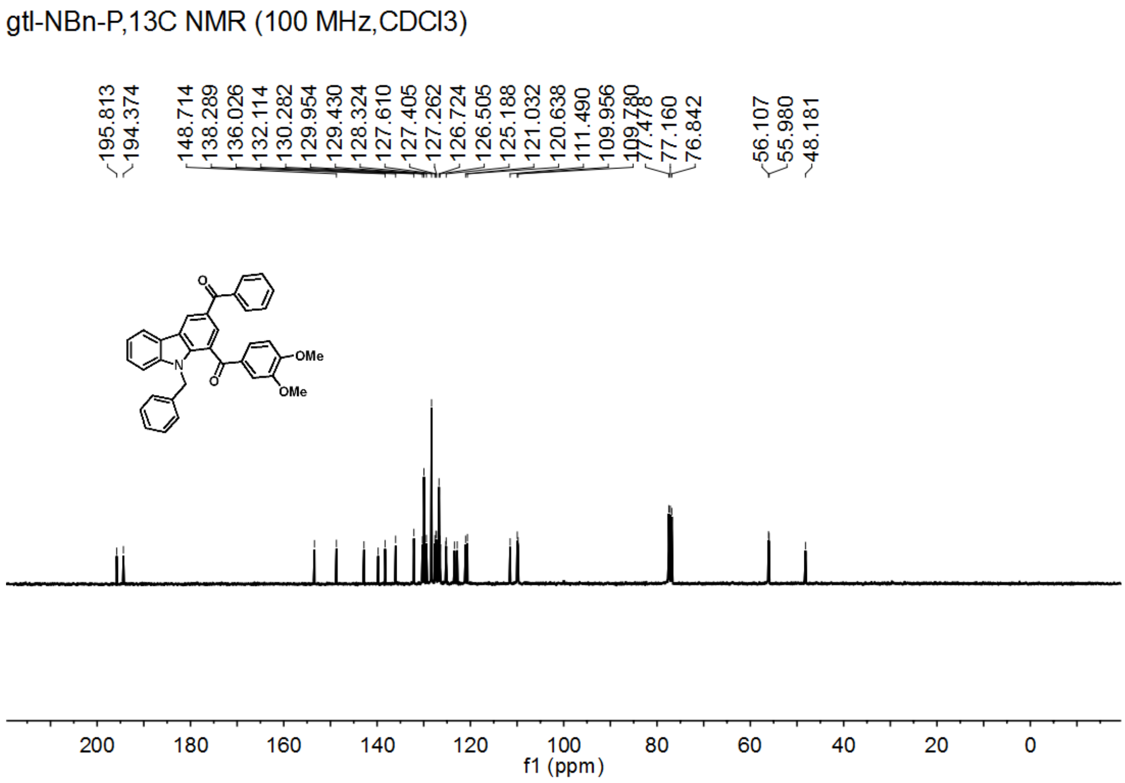

Supplementary Fig. 17. ^{1}H NMR and ^{13}C NMR spectra for **3e**.

gtl-YD-5-CH3,1H NMR (400 MHz,CDCl3)

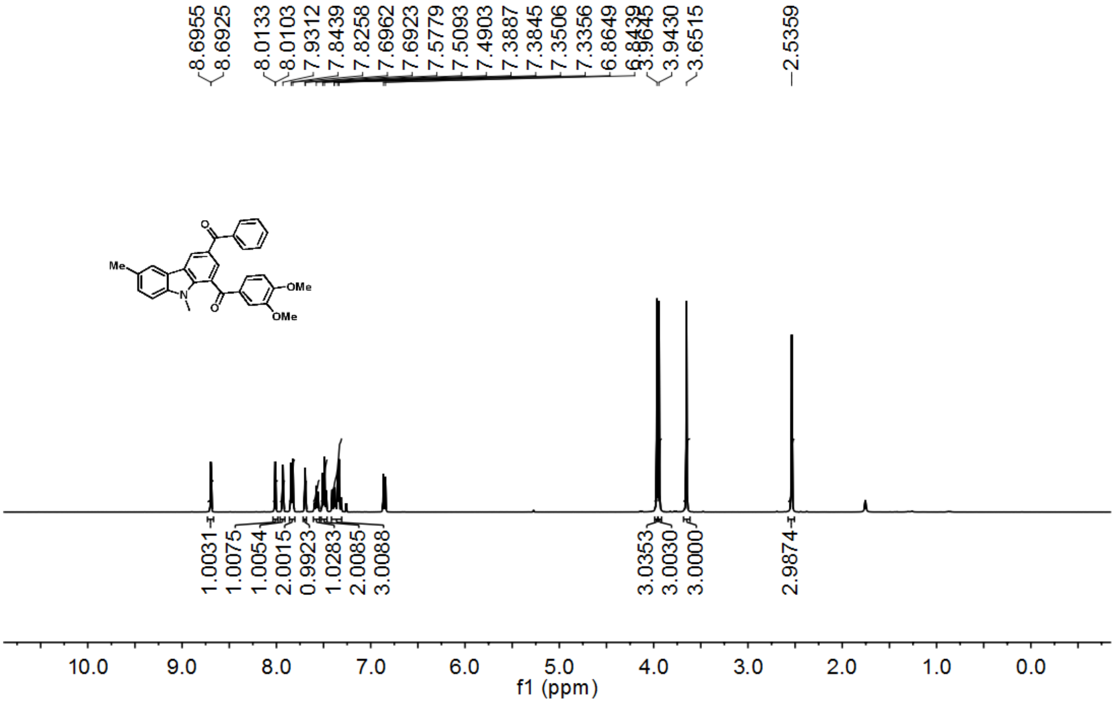

gtl-YD-5-CH3,13C NMR (100 MHz, CDCl3)

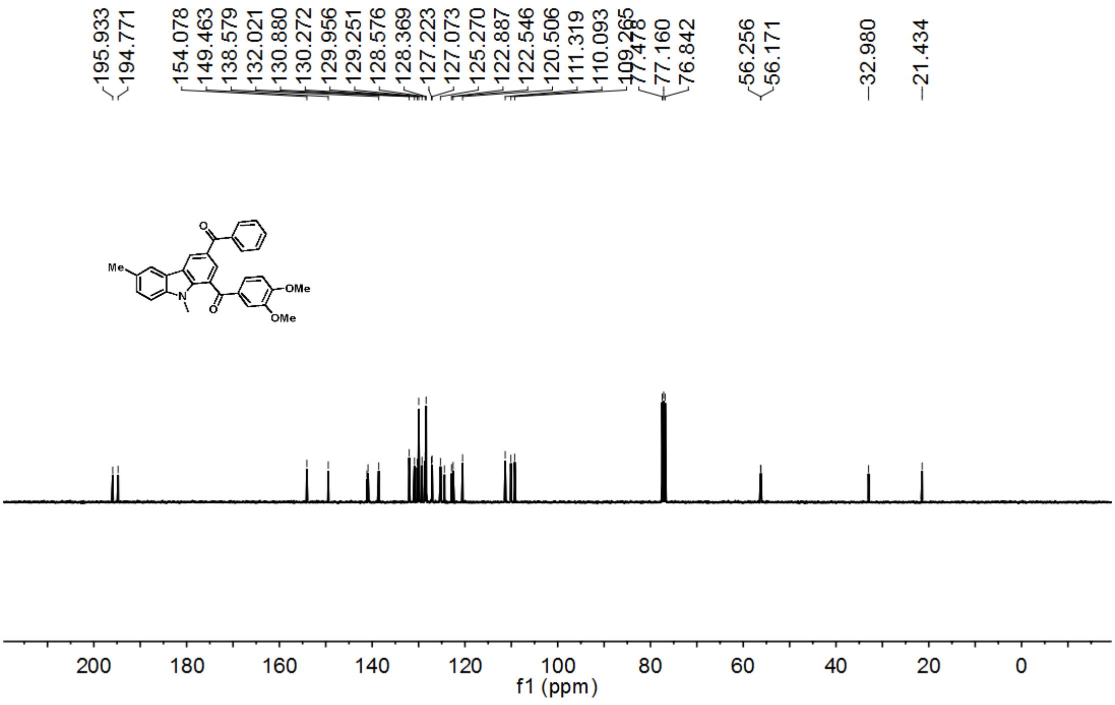

Supplementary Fig. 18. ^{1}H NMR and ^{13}C NMR spectra for **3f**.

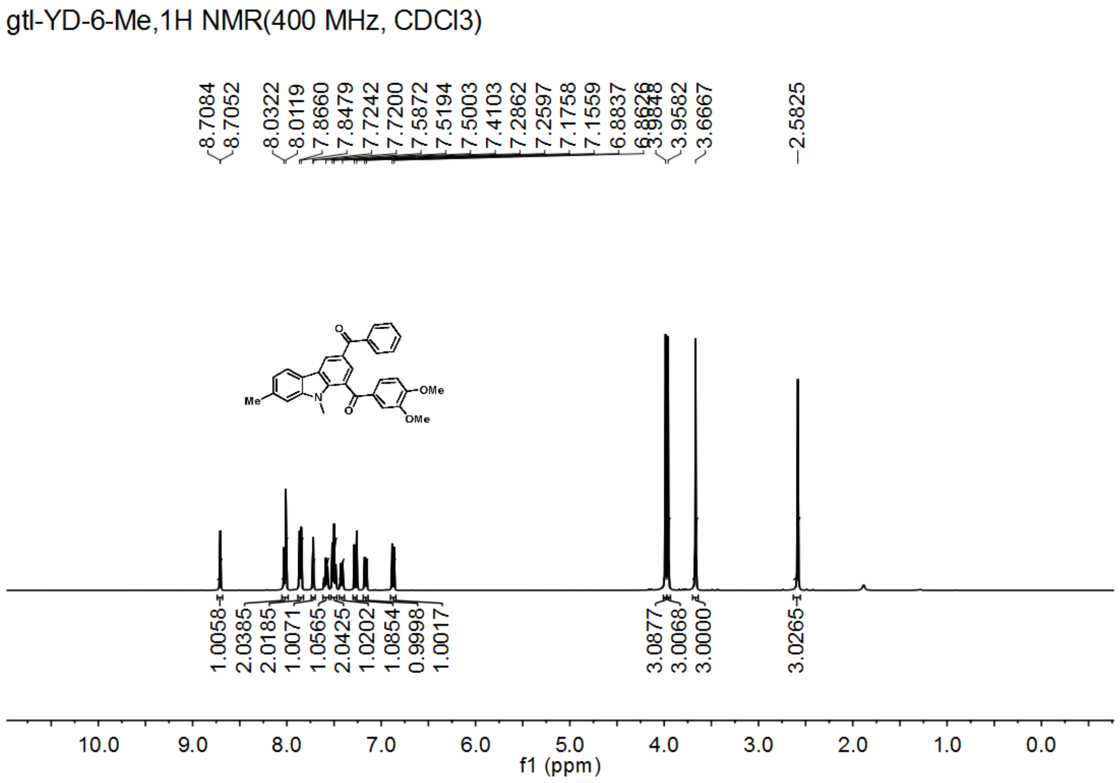

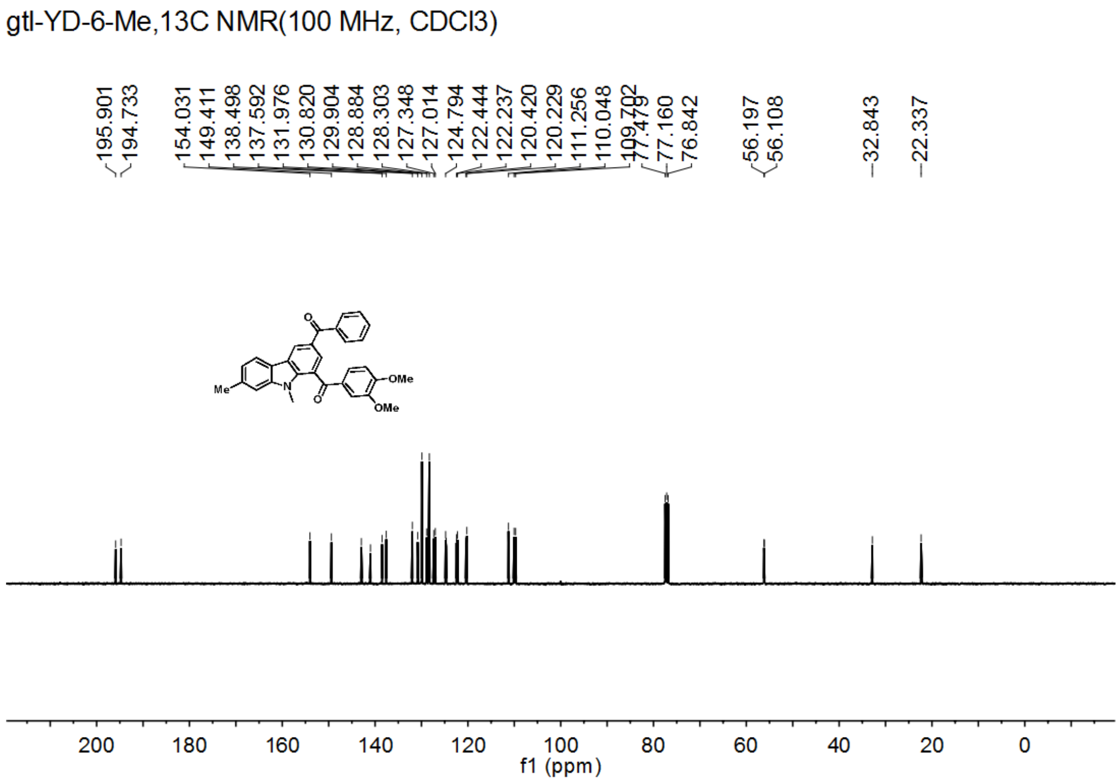

Supplementary Fig. 19. ^{1}H NMR and ^{13}C NMR spectra for **3g**.

gtl-YD-OMe,1H NMR (400 MHz,CDCl3)

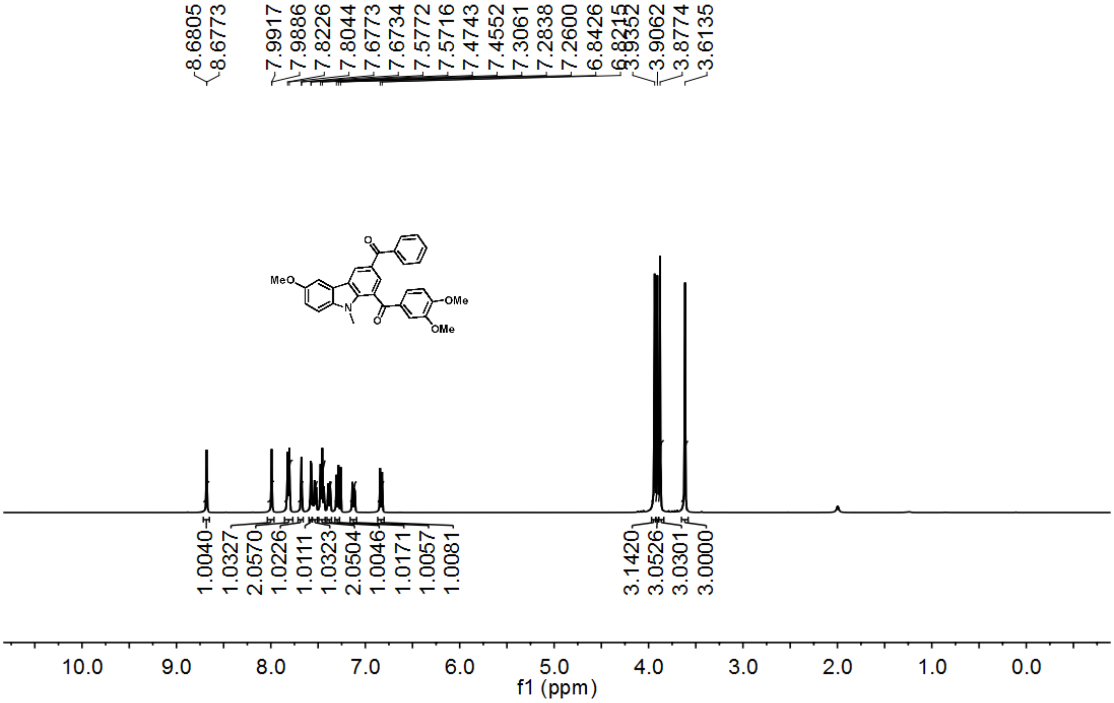

gtl-YD-OMe,13C NMR (100 MHz, CDCl3)

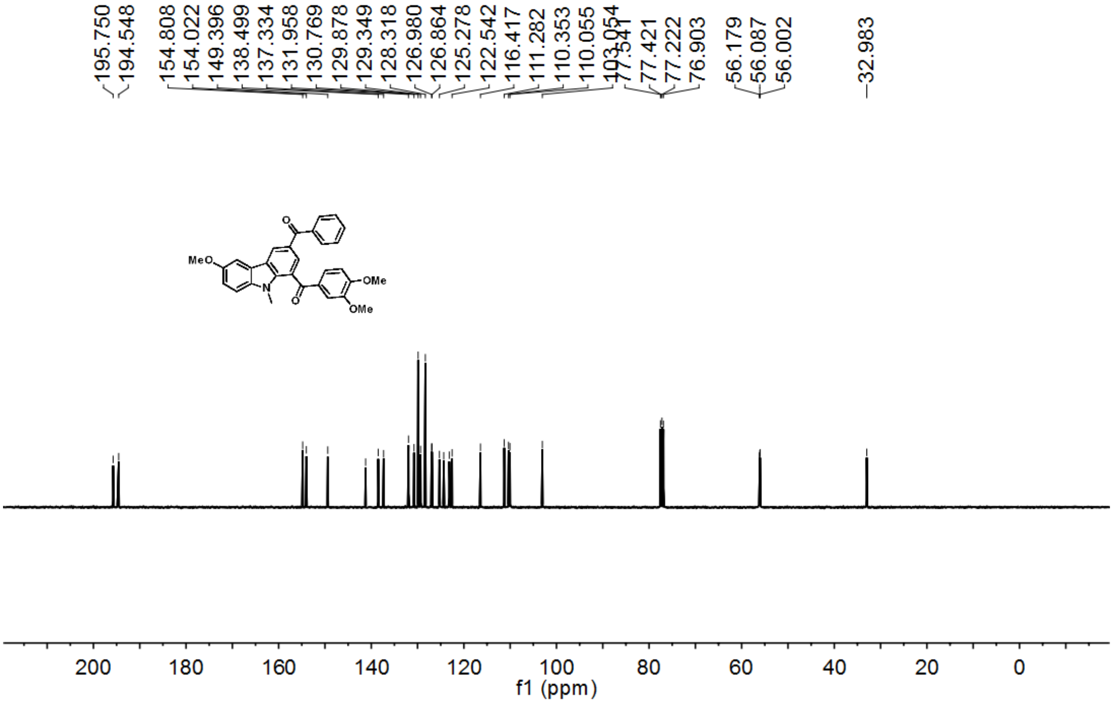

Supplementary Fig. 20. ^{1}H NMR and ^{13}C NMR spectra for **3h**.

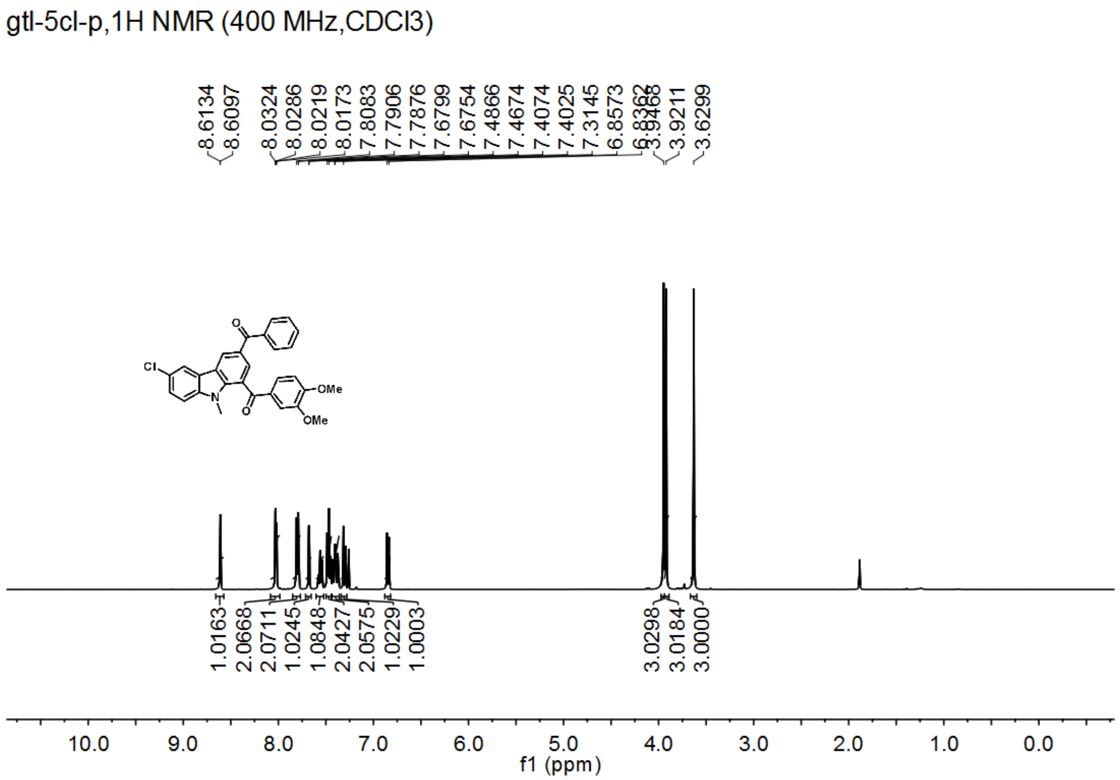

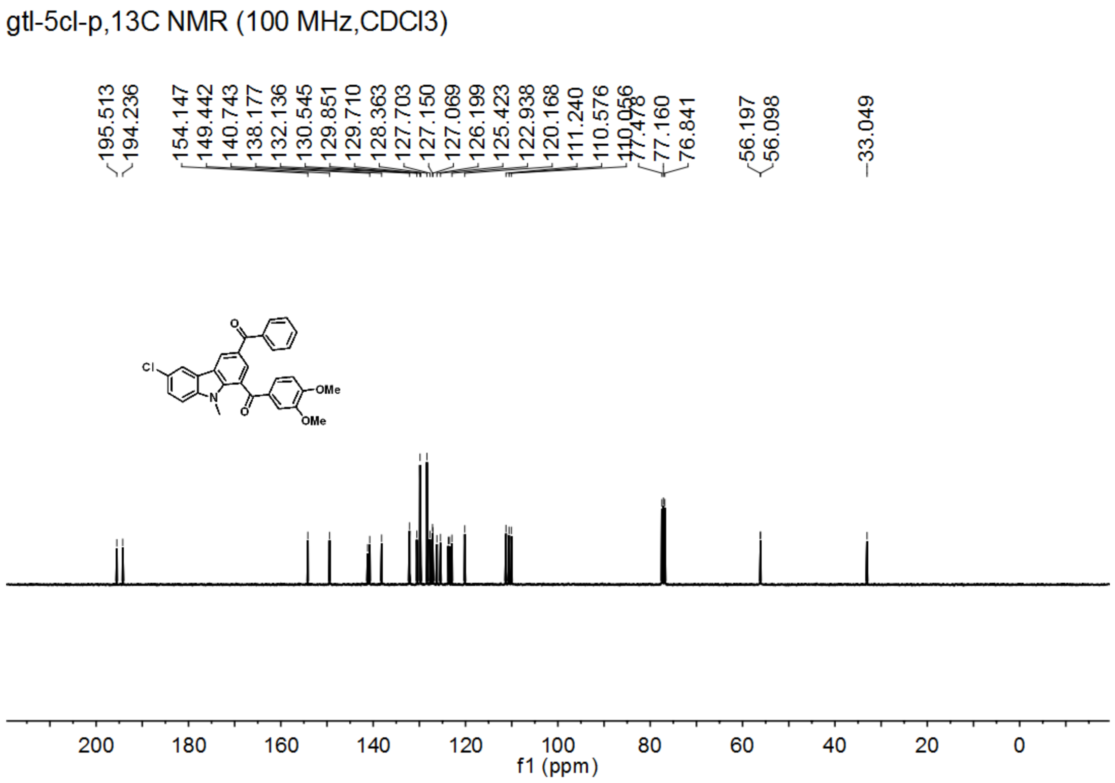

Supplementary Fig. 21. ^{1}H NMR and ^{13}C NMR spectra for **3i**.

gtl-ome-p,1H NMR (400 MHz,CDCl3)

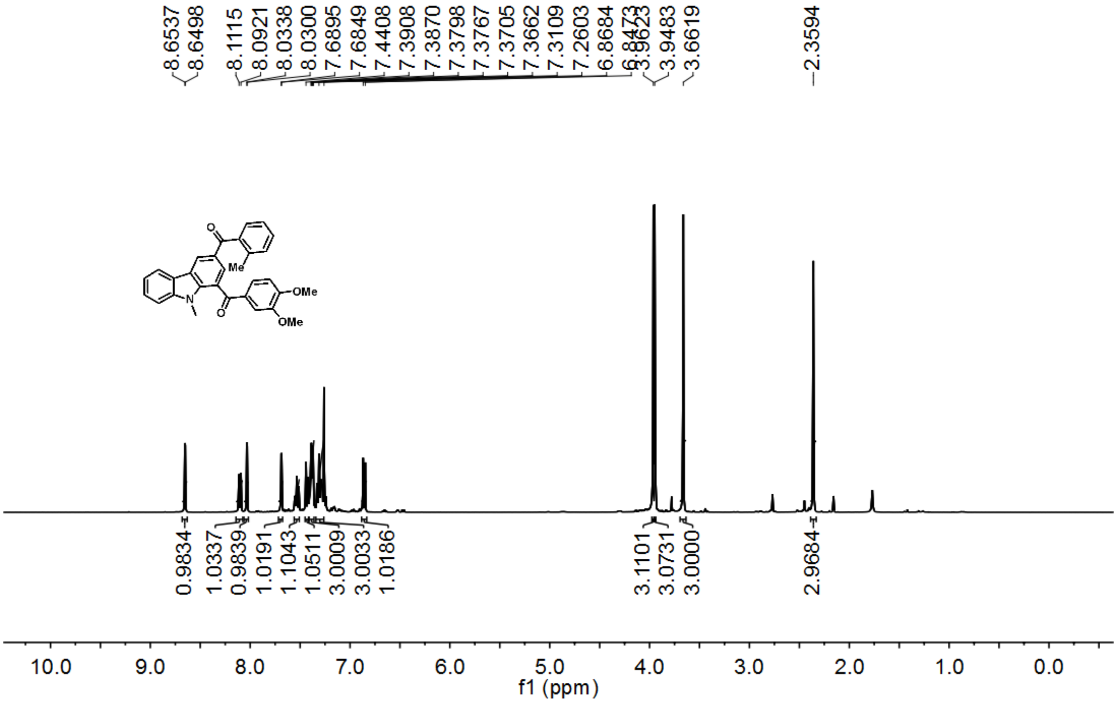

gtl-ome-p,13C NMR (100 MHz, CDCl3)

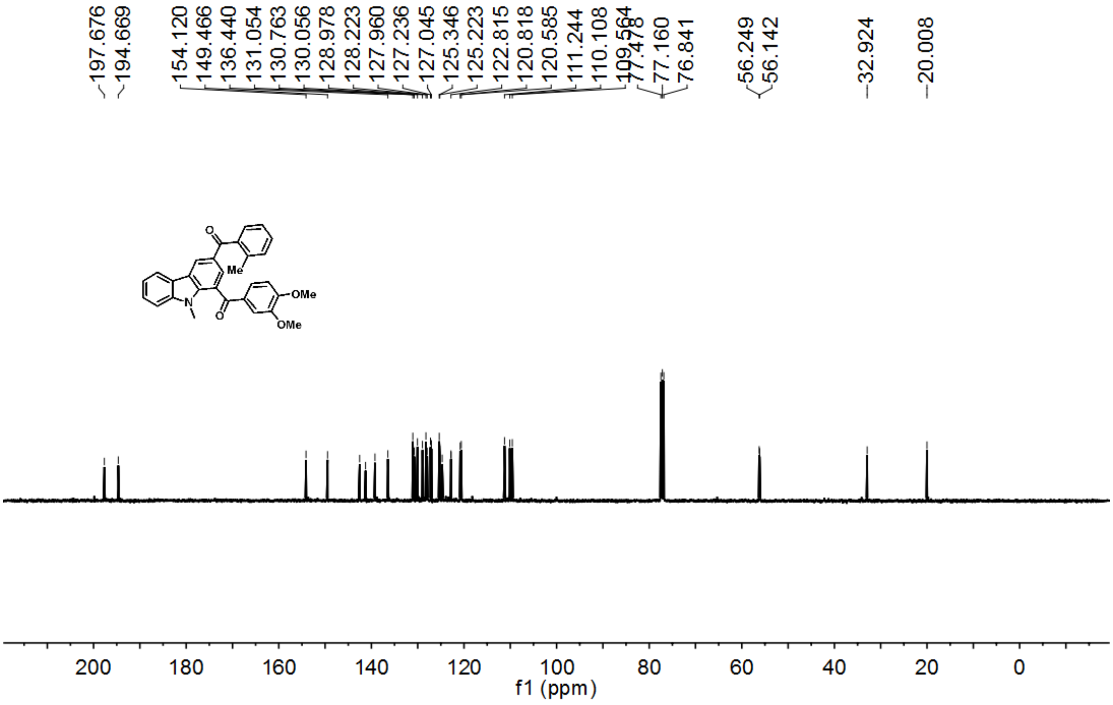

Supplementary Fig. 22. ^{1}H NMR and ^{13}C NMR spectra for **3j**.

gtl-m-Me,13C NMR (100 MHz, CDCl3)

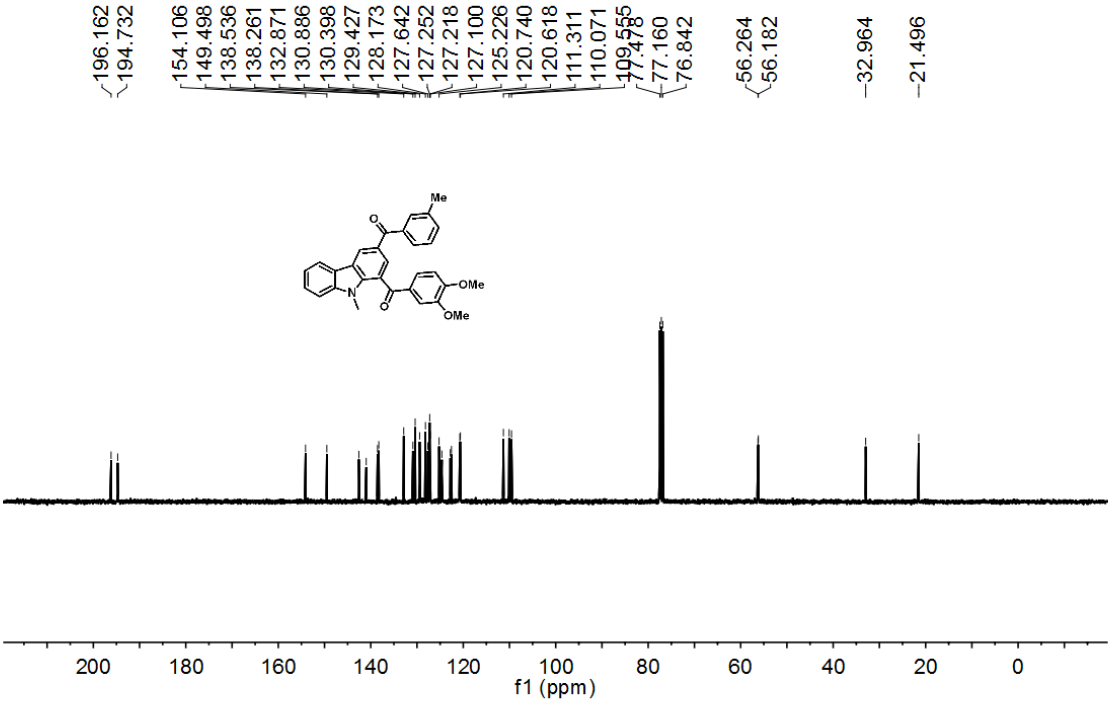

gtl-m-Me,1H NMR (400 MHz, CDCl3)

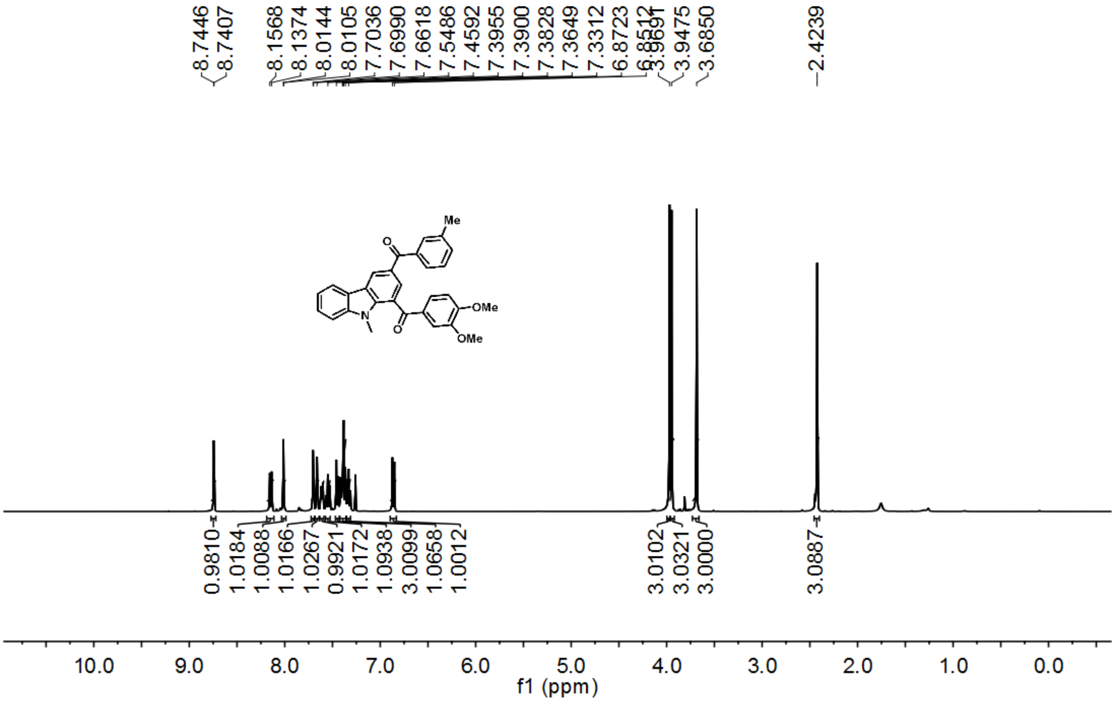

Supplementary Fig. 23. ^{1}H NMR and ^{13}C NMR spectra for **3k**.

gtl-p-me,1H NMR (400 MHz, CDCl3)

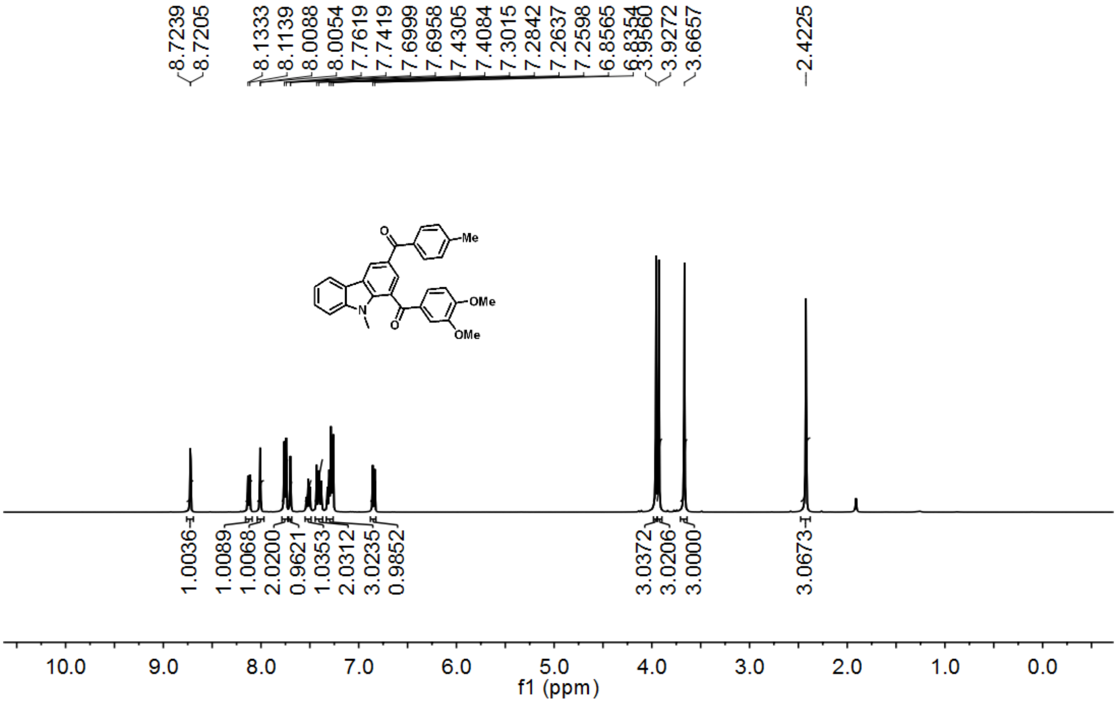

gtl-p-me,13C NMR (100 MHz, CDCl3)

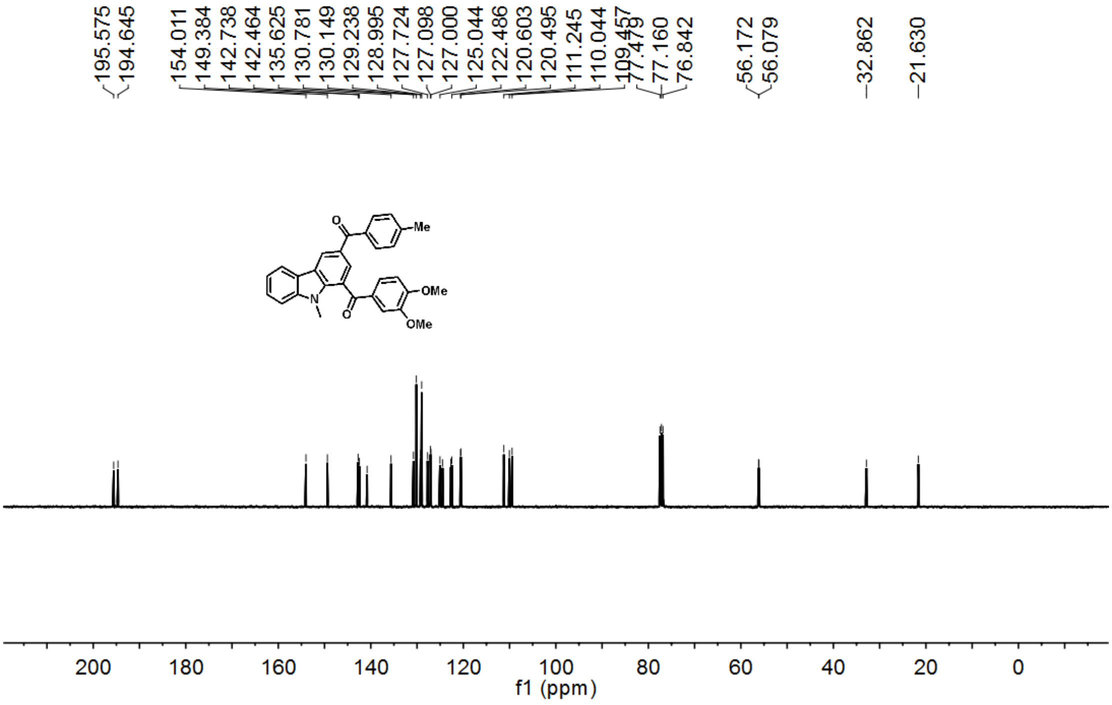

Supplementary Fig. 24. ^{1}H NMR and ^{13}C NMR spectra for **3l**.

gtl-MeO-P,1H NMR (400 MHz,CDCl3)

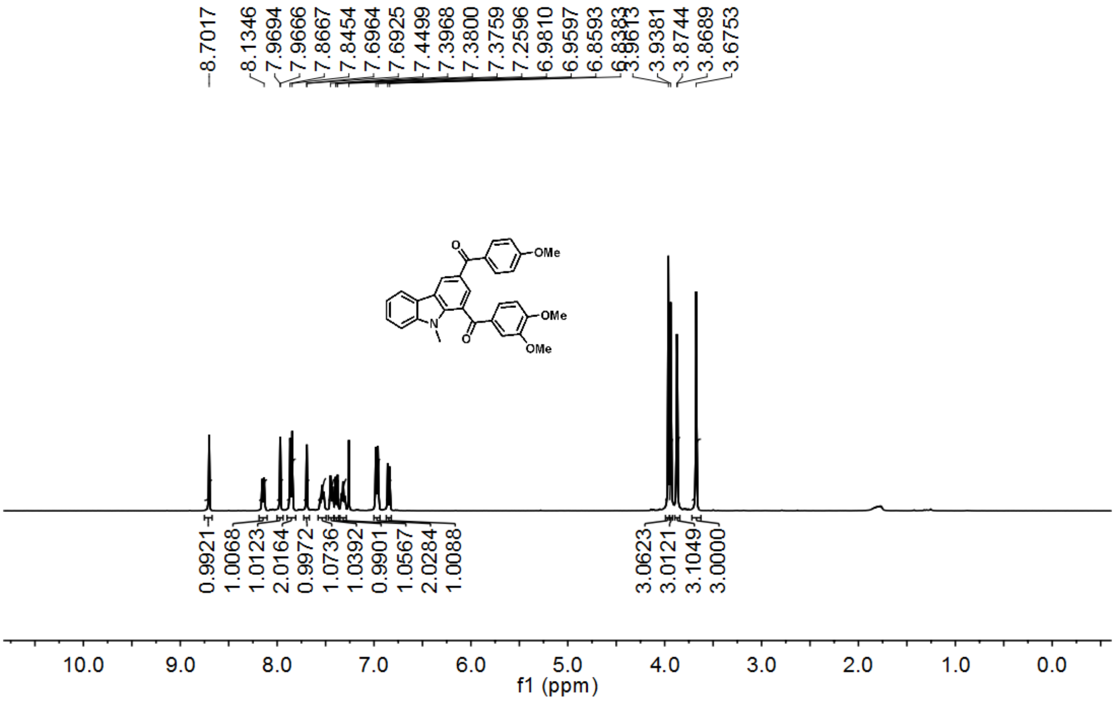

gtl-MeO-P,13C NMR (100 MHz, CDCl3)

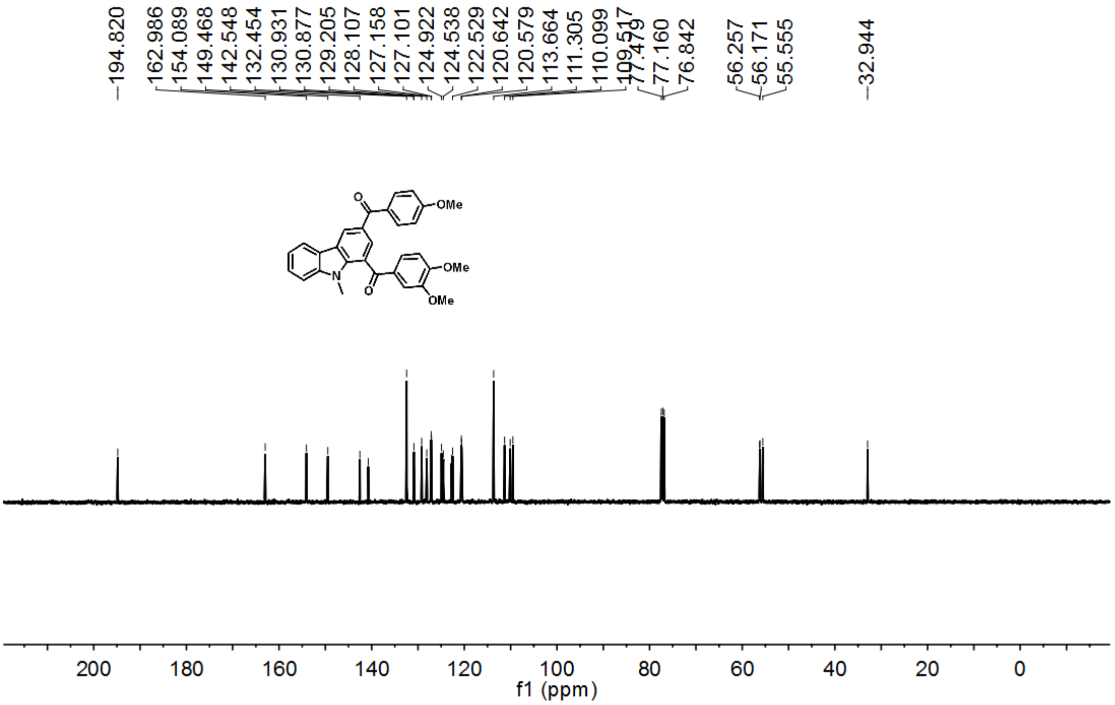

Supplementary Fig. 25. ^{1}H NMR and ^{13}C NMR spectra for **3m**.

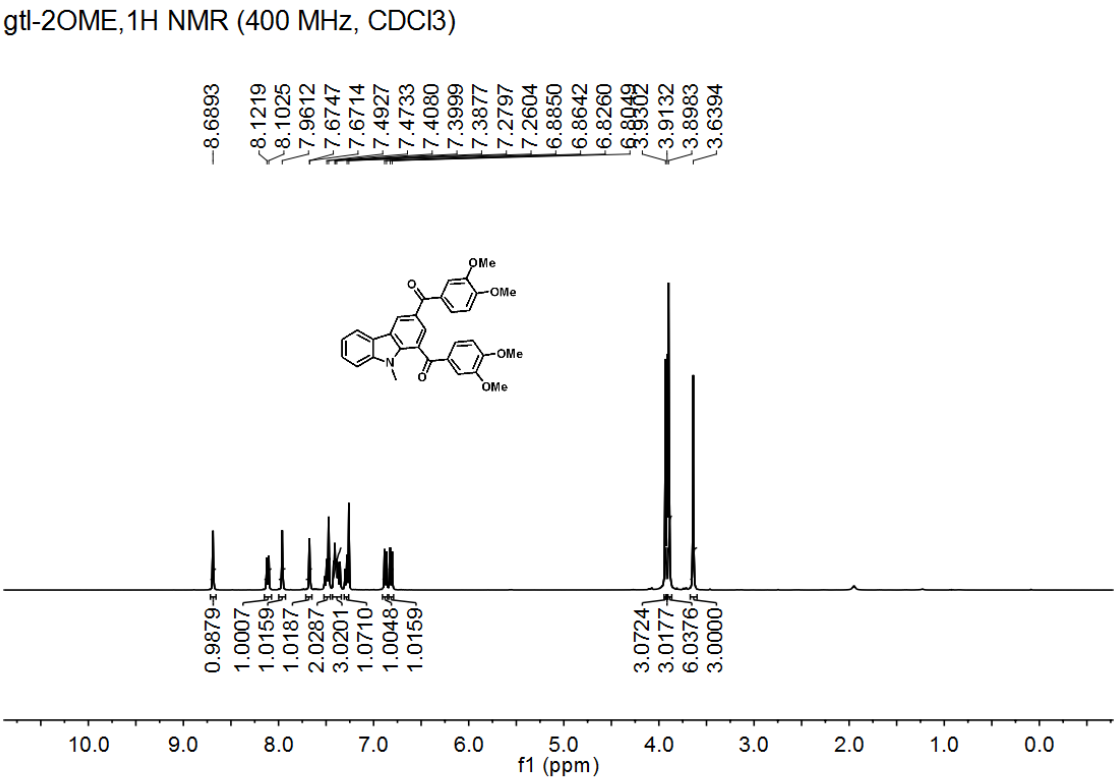

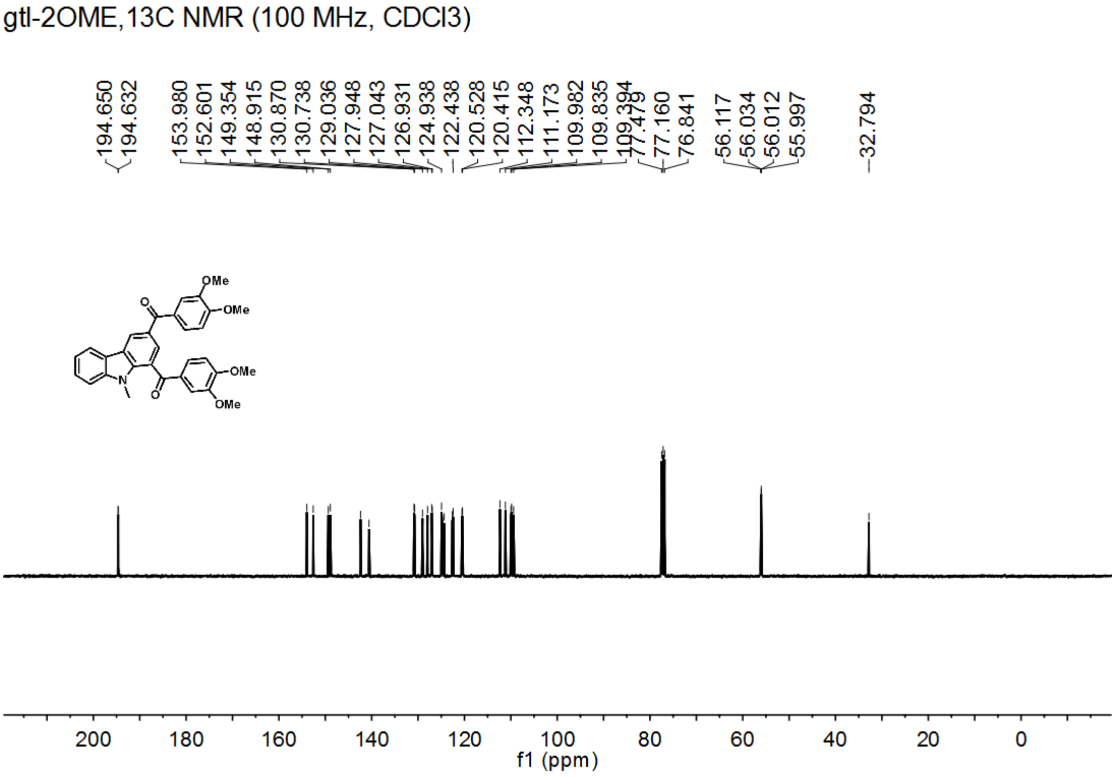

Supplementary Fig. 26. ^{1}H NMR and ^{13}C NMR spectra for **3n**.

gtl-p-f,1H NMR (400 MHz,CDCl3)

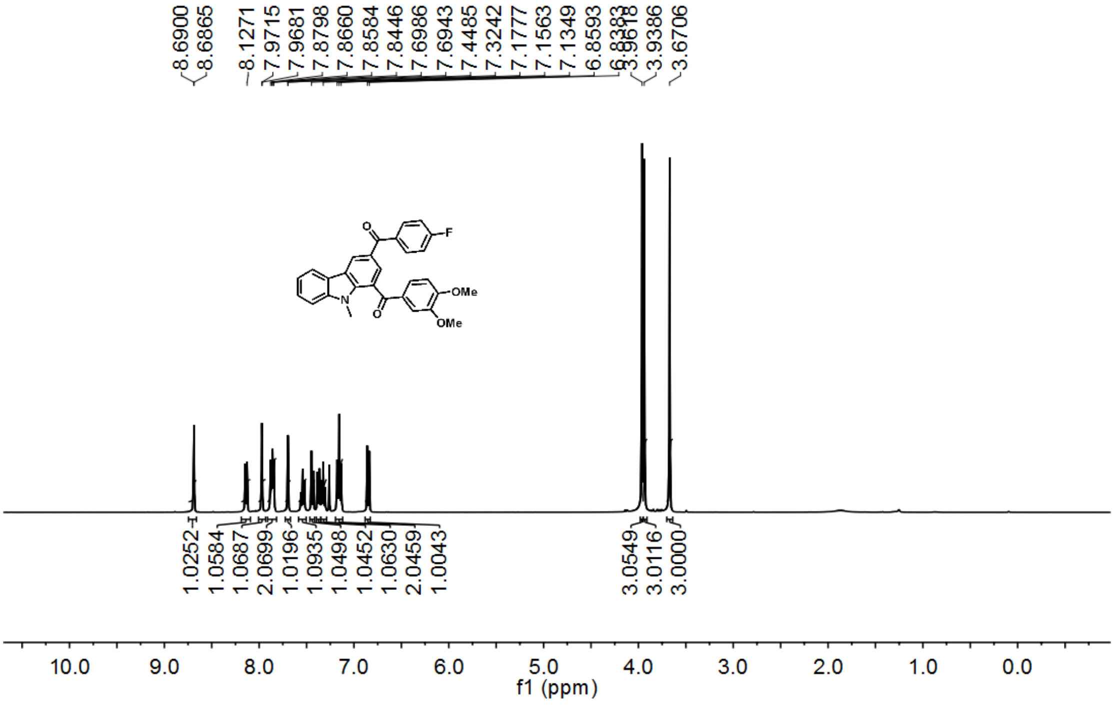

gtl-p-f,13C NMR (100 MHz,CDCl3)

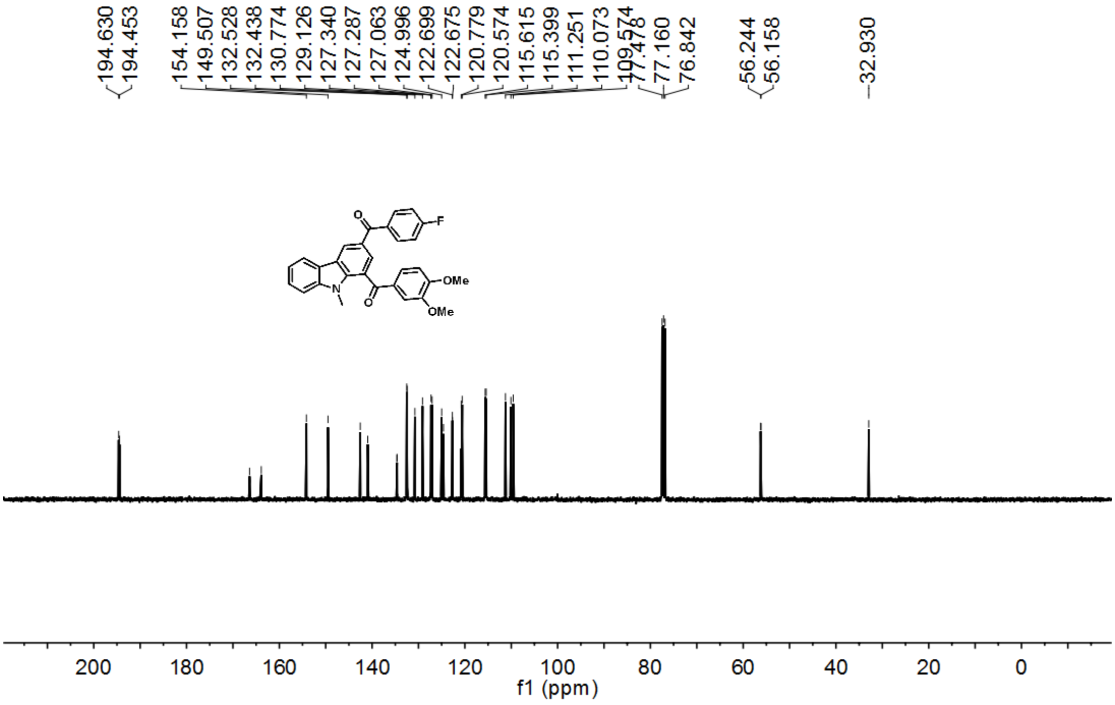

Supplementary Fig. 27. ^{1}H NMR and ^{13}C NMR spectra for **3o**.

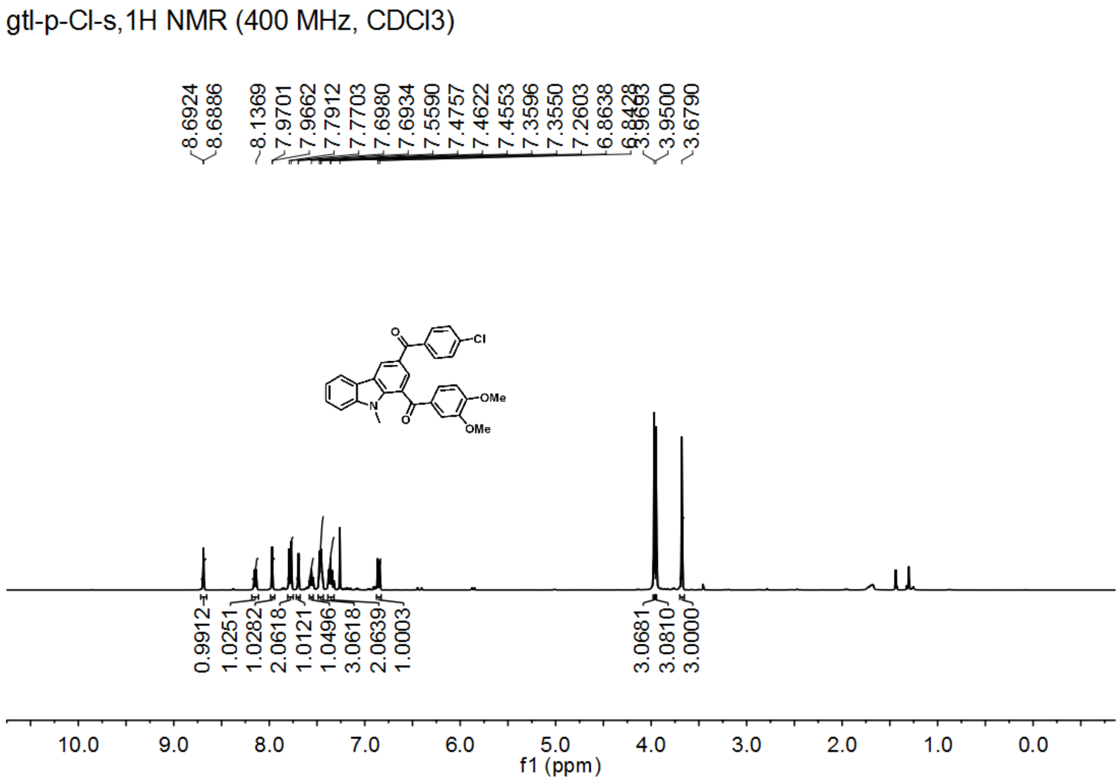

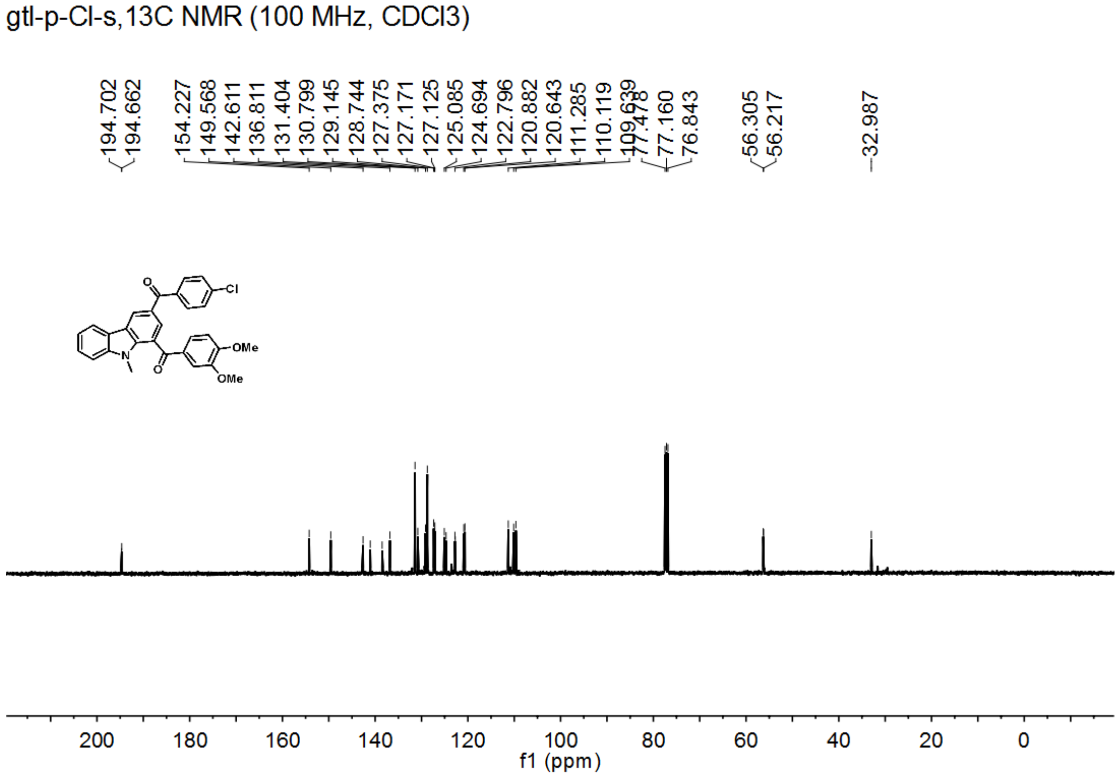

Supplementary Fig. 28. ^{1}H NMR and ^{13}C NMR spectra for **3p**.

GTL-cn,1H NMR (400 MHz, CDCl3)

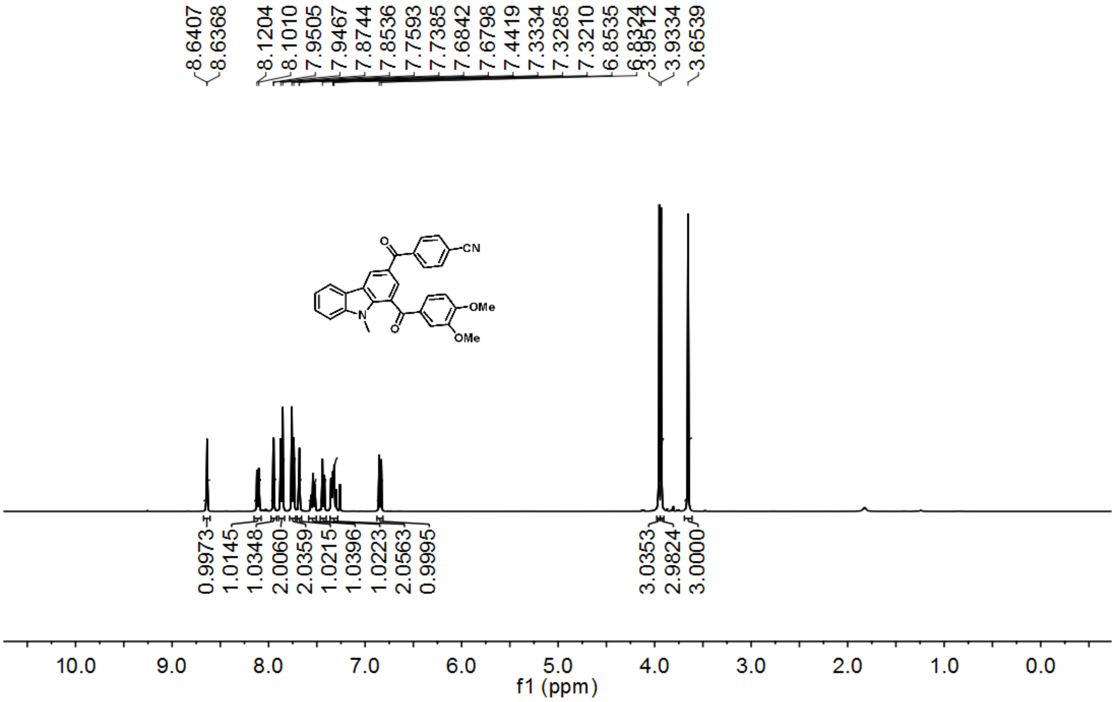

GTL-cn,13C NMR (100 MHz, CDCl3)

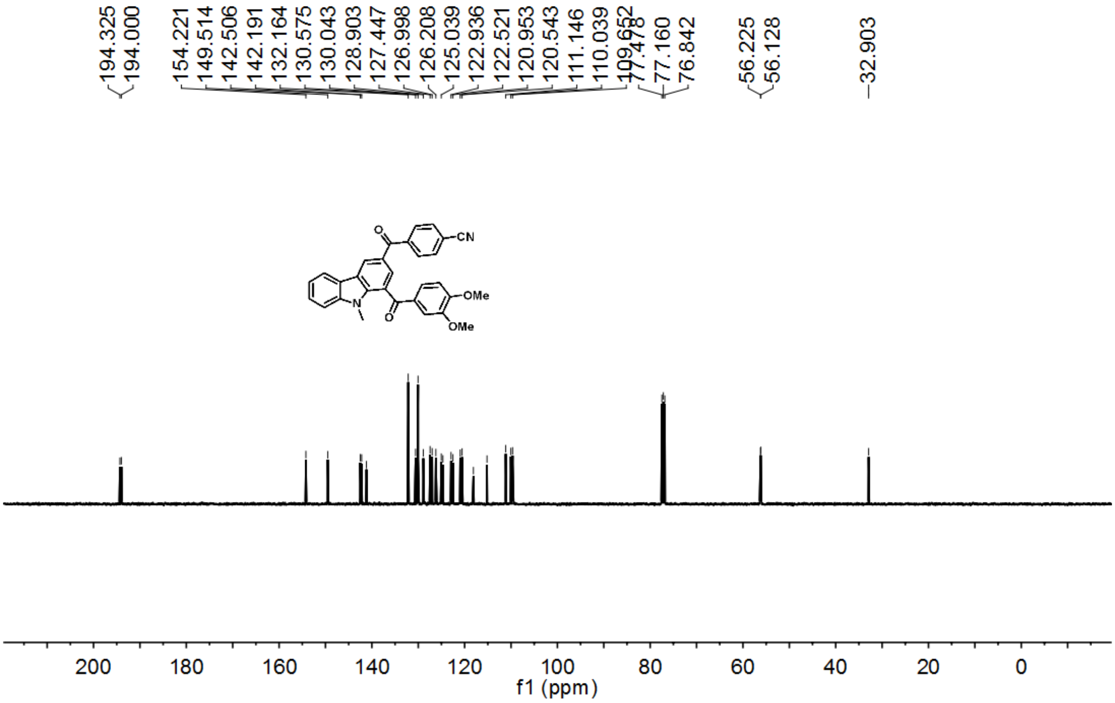

Supplementary Fig. 29. ^{1}H NMR and ^{13}C NMR spectra for **3q**.

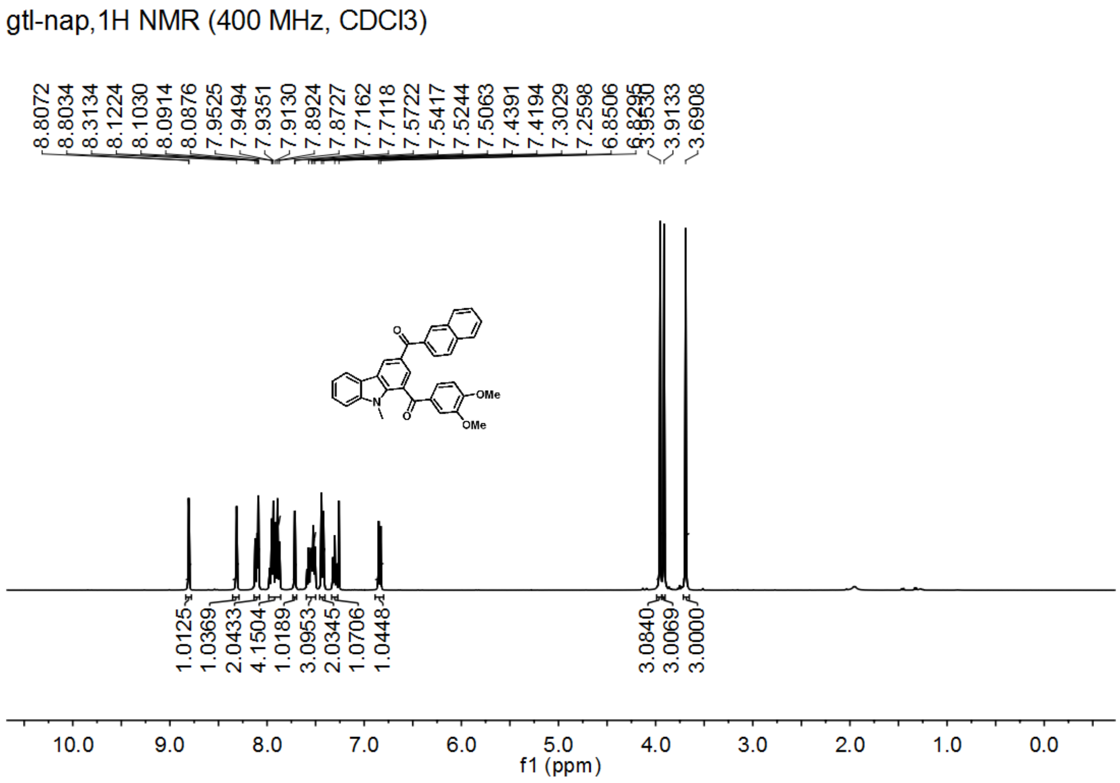

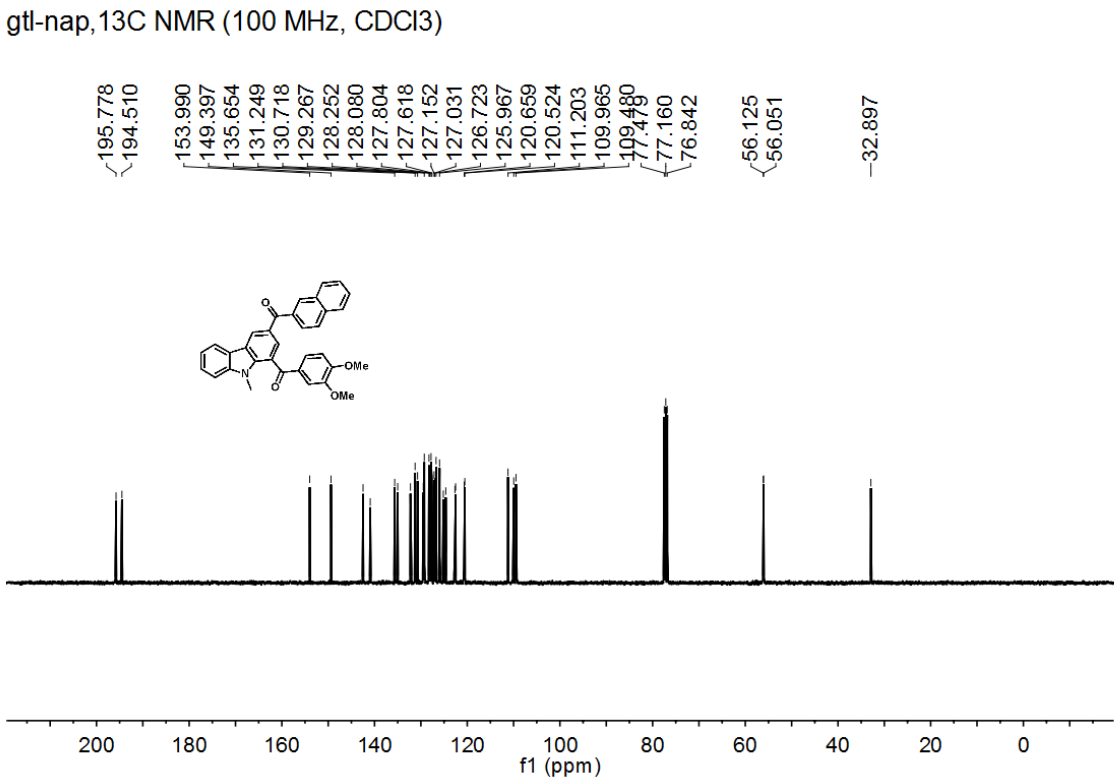

Supplementary Fig. 30. ^{1}H NMR and ^{13}C NMR spectra for **3r**.

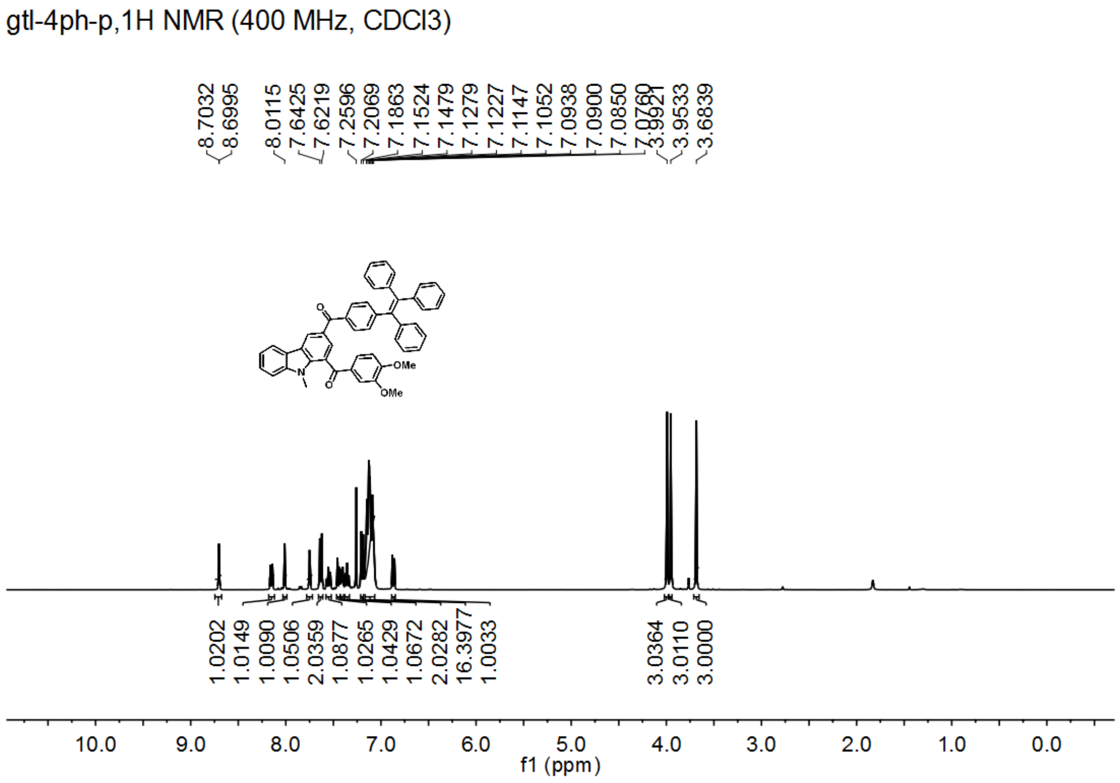

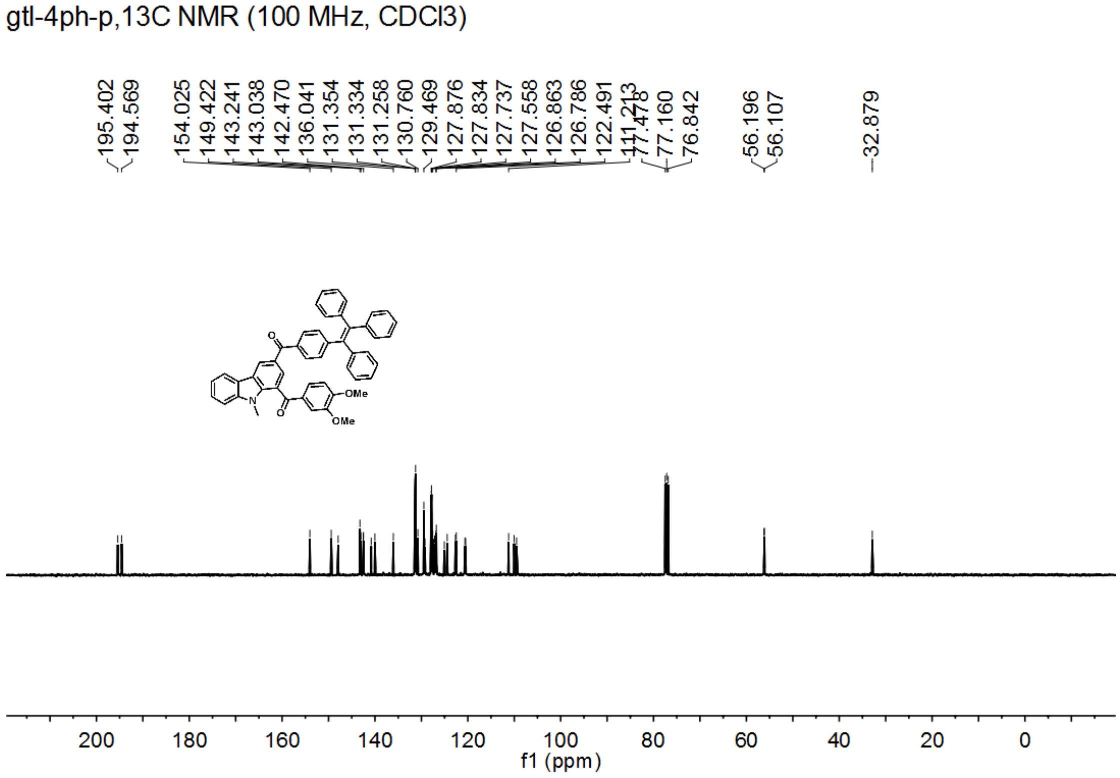

Supplementary Fig. 31. ^{1}H NMR and ^{13}C NMR spectra for **3s**.

GTL-S-P,1H NMR (400 MHz, CDCl3)

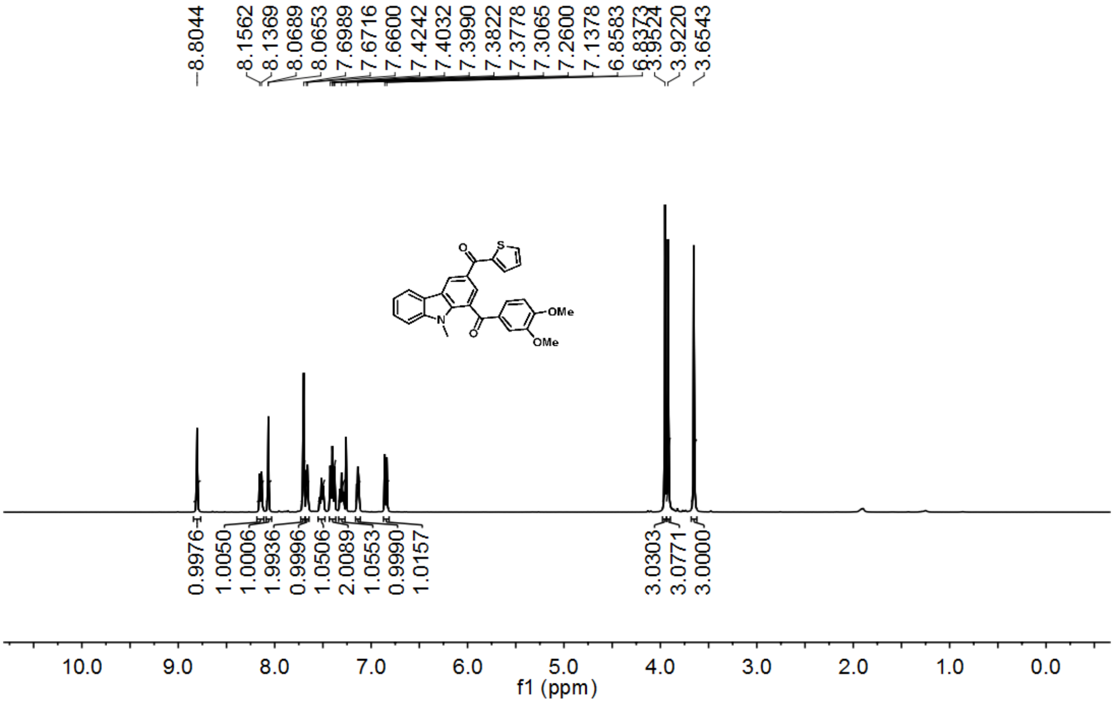

GTL-S-P,13C NMR (100 MHz, CDCl3)

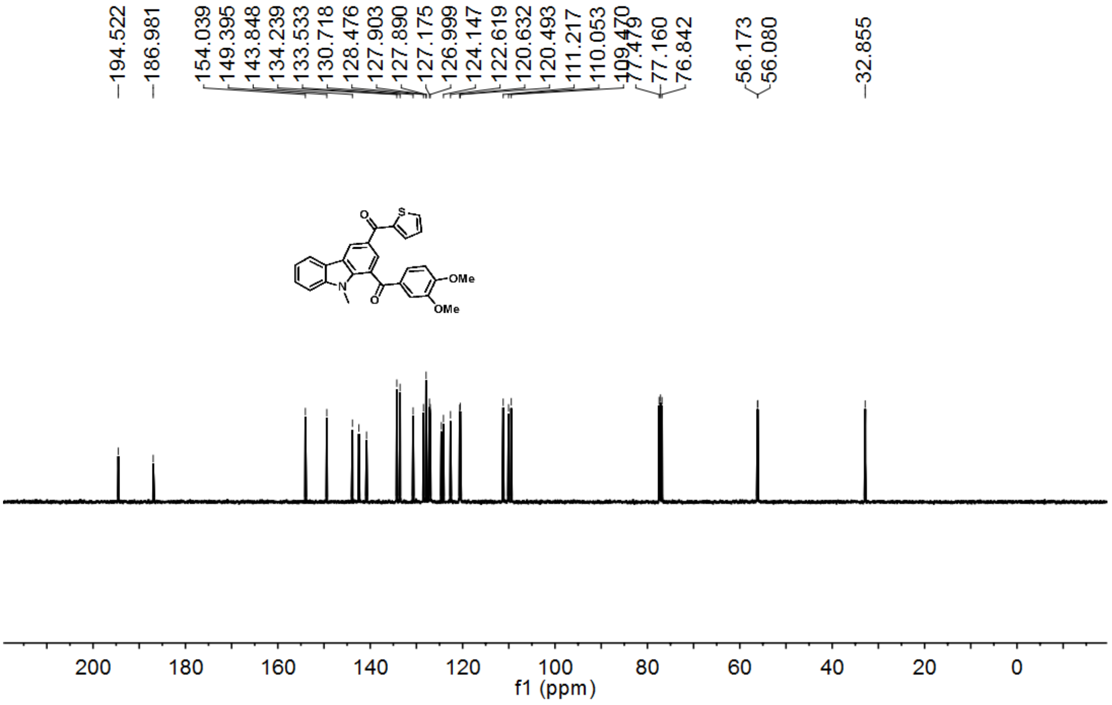

Supplementary Fig. 32. ^{1}H NMR and ^{13}C NMR spectra for **3t**.

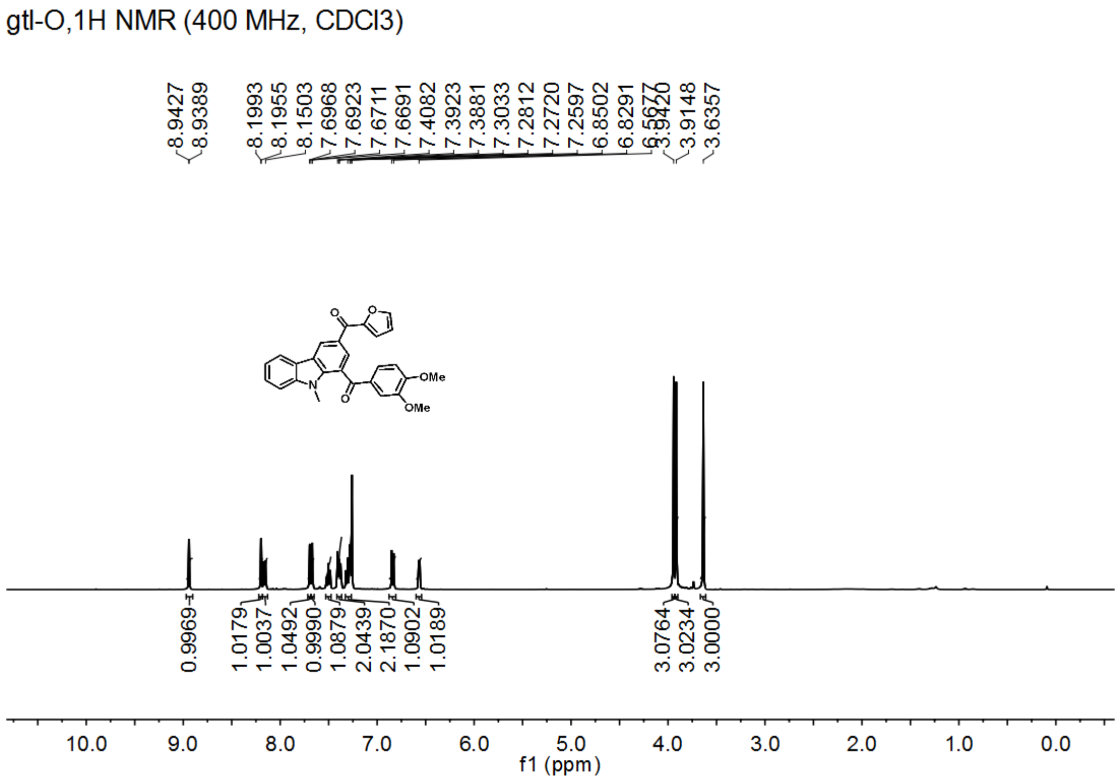

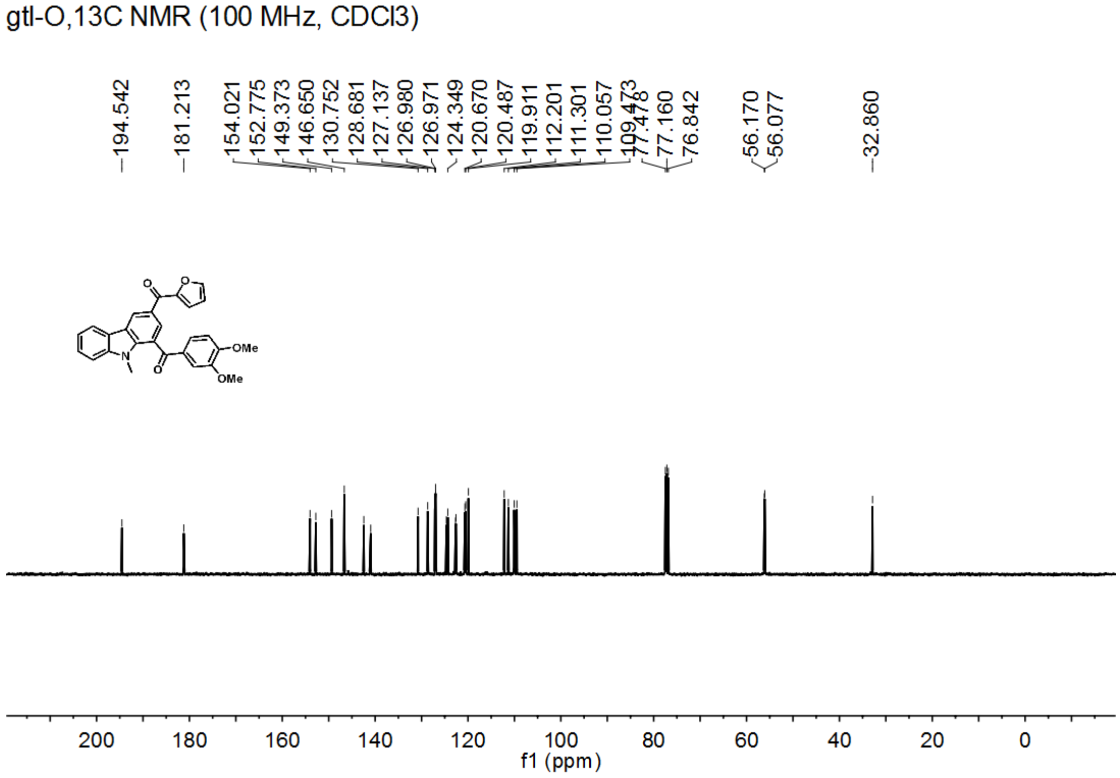

Supplementary Fig. 33. ^{1}H NMR and ^{13}C NMR spectra for **3u**.

gtl-hbw-p,1H NMR (400 MHz, CDCl3)

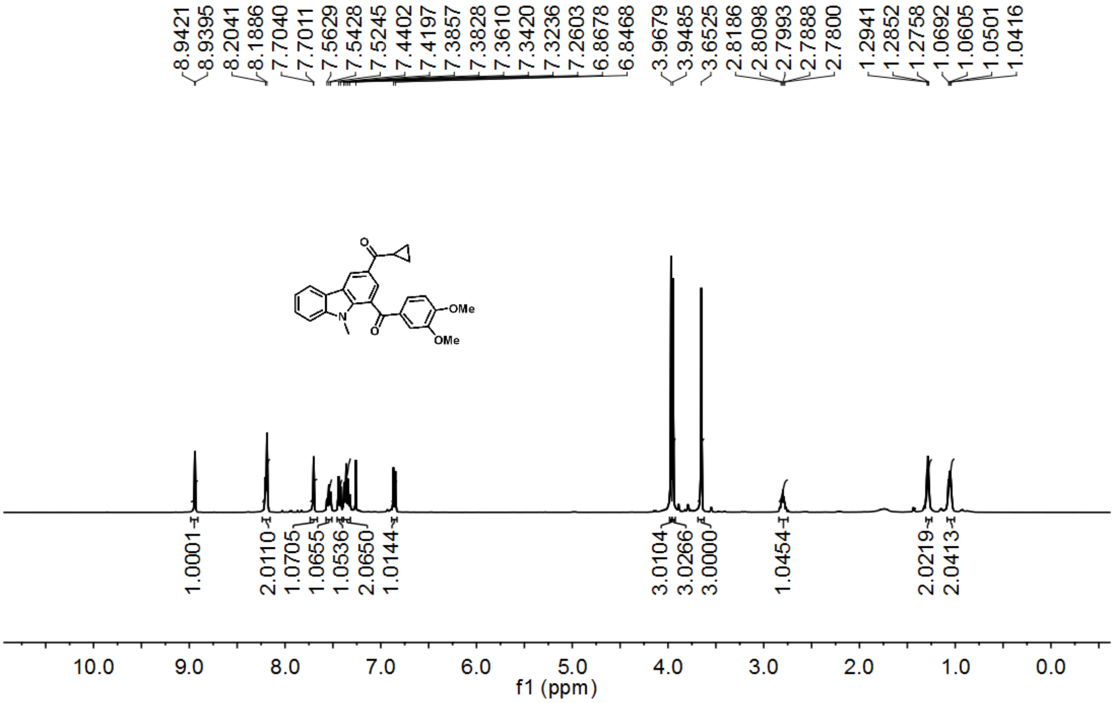

gtl-hbw-p,13C NMR (100 MHz,CDCl3)

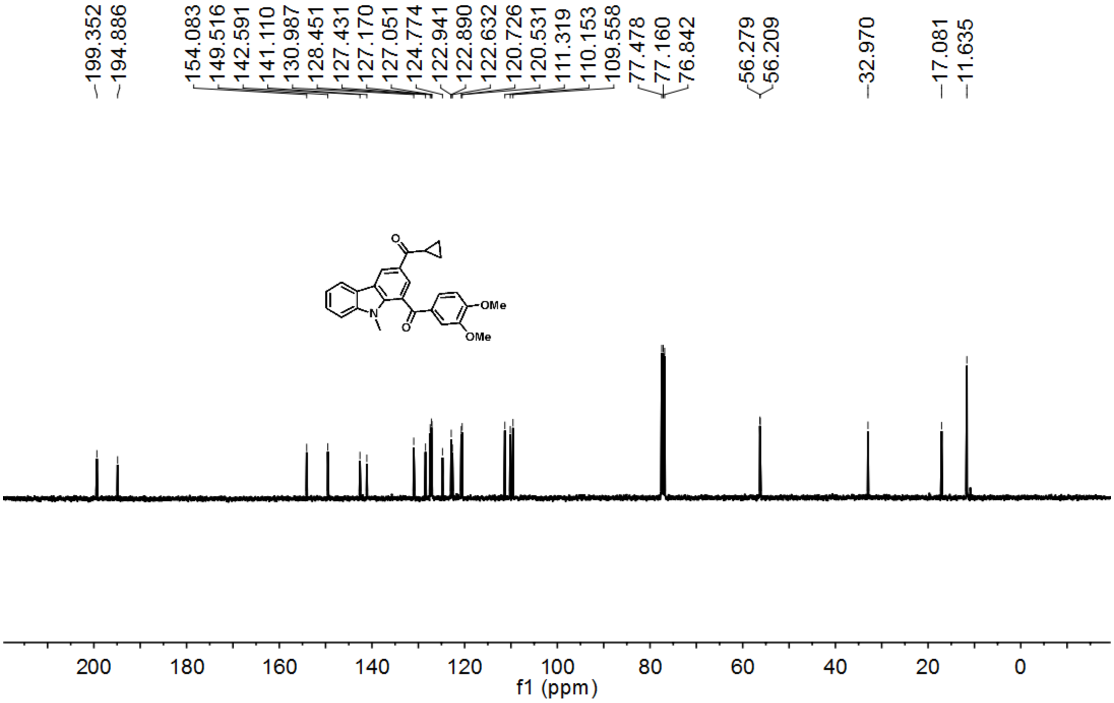

Supplementary Fig. 34. ^{1}H NMR and ^{13}C NMR spectra for **3v**.

gtl-NH,1H NMR(400 MHz,CDCl3)

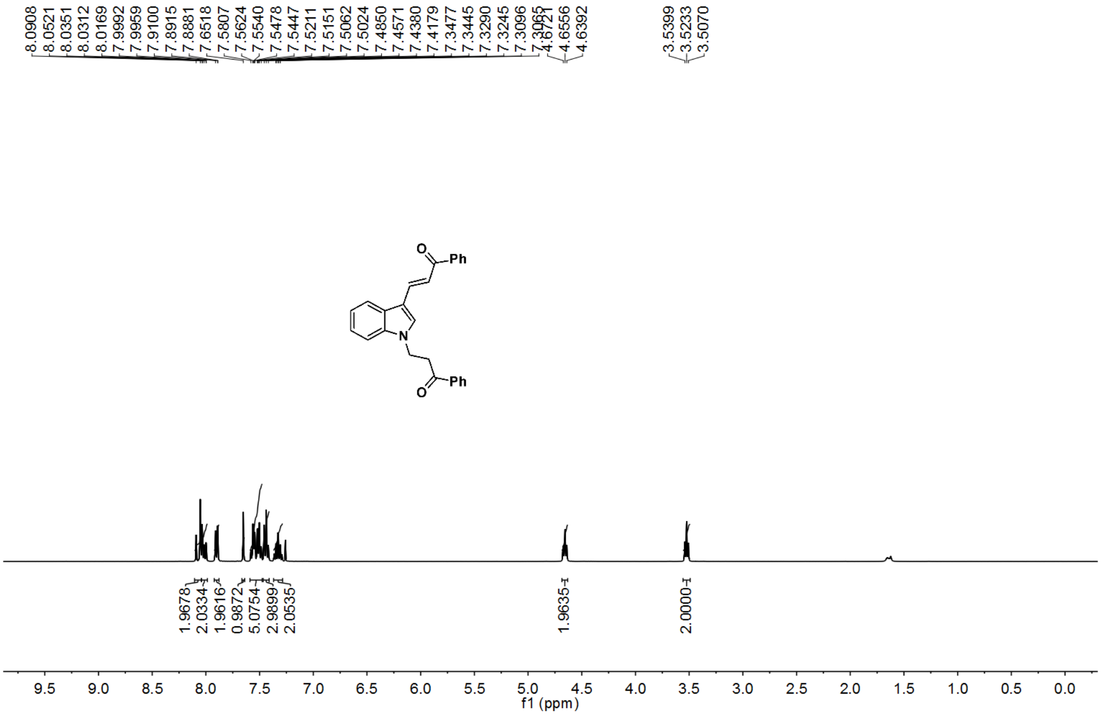

gtl-NH,13C NMR(100 MHz,CDCl3)

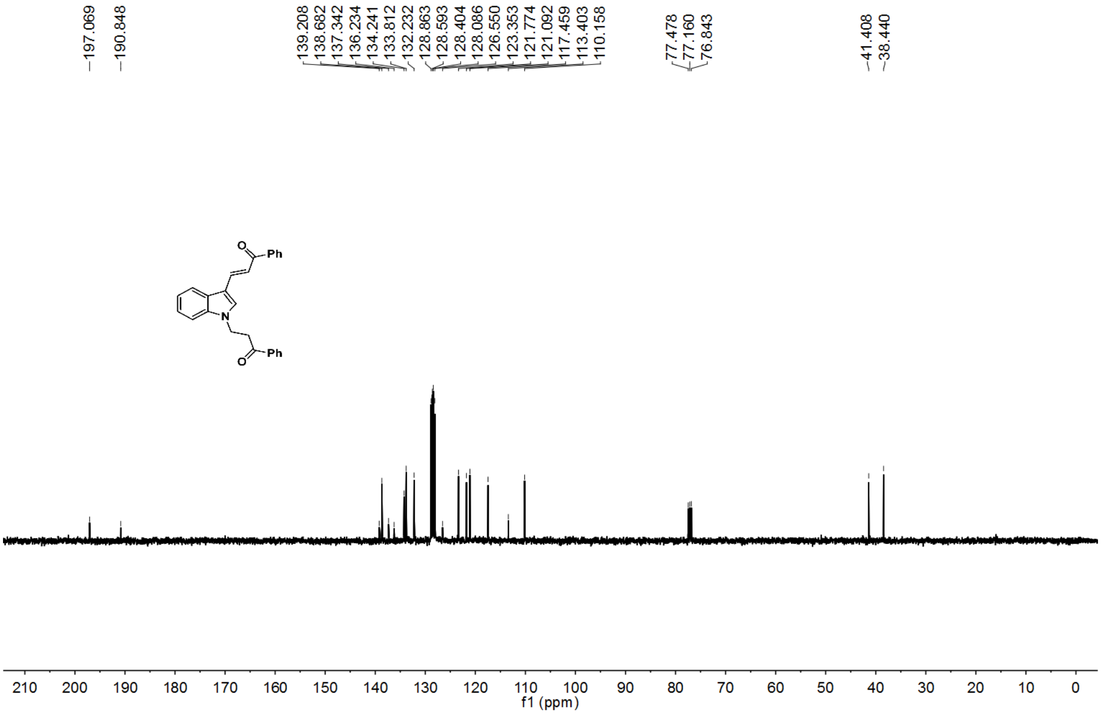

Supplementary Fig. 35. ^{1}H NMR and ^{13}C NMR spectra for (*E*)-3-(1-(3-oxo-3-phenylpropyl)-1*H*-indol-3-yl)-1-phenylprop-2-en-1-one.

2.3 Computational details

Supplementary Table 4. Table of Calculated Energies and Energy Corrections

Stationary point	Single-point energy-(u)B3LYP-D3(BJ)-SMD/ LANL2TZ(*f*)&6-311+G(*d,p*) (a.u.)	Thermal correction to Gibbs free energy at 298.15 K (a.u.)
MeOH	-115.770994	0.028703
**1a**	-1150.881445	0.328938
Cat^{CSS}	-1169.698798	0.413670
TS1^{CSS}	-2320.564046	0.764408
IM1^{CSS}	-2204.811018	0.717167
TS2^{OSS}	-2204.766619	0.710831
IM2^{OSS}	-2204.808333	0.710738
IM2^{triplet}	-2204.831950	0.711736
TS3^{triplet}	-2204.797258	0.710982
IM3^{triplet}	-2204.811936	0.708148
TS4^{triplet}	-2204.774365	0.704289
TS3^{OSS}	-2204.794875	0.712488
Ar^{2}OH	-422.154114	0.105552
IM3^{CSS}	-1782.661915	0.585246
TS4^{CSS}	-1782.618676	0.581960
5a'	-652.256325	0.166149
Cat'^{CSS}	-1130.382676	0.387273
TS4^{triplet}	-2320.571947	0.759949

2.4 The photophysical property of carbazoles

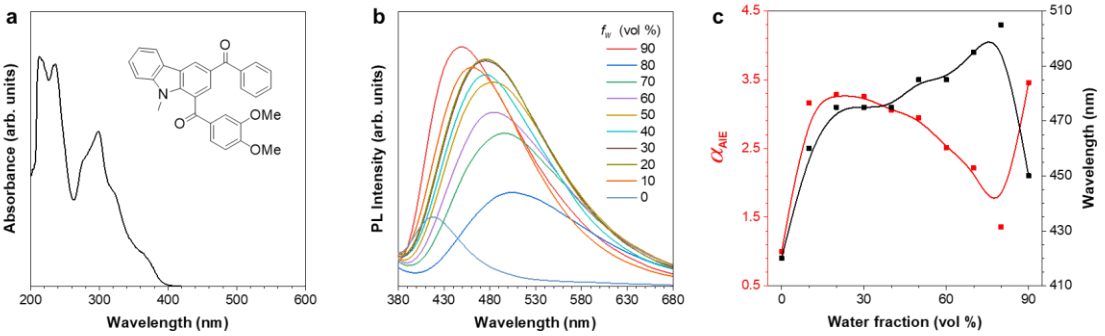

Supplementary Fig. 36 The photophysical property of **3a. a** Absorption spectra of **3a** in pure THF. **b** PL spectra of **3a** in THF/H_2O mixtures with different f_w. λ_{ex}: 299 nm. Concentration: 20 μM. **c** The plots of the α_{AIE} and maximum emission wavelength versus the composition of the aqueous mixture of **3a**, $\alpha_{AIE} = I/I_0$, I_0 = PL intensity in pure THF.

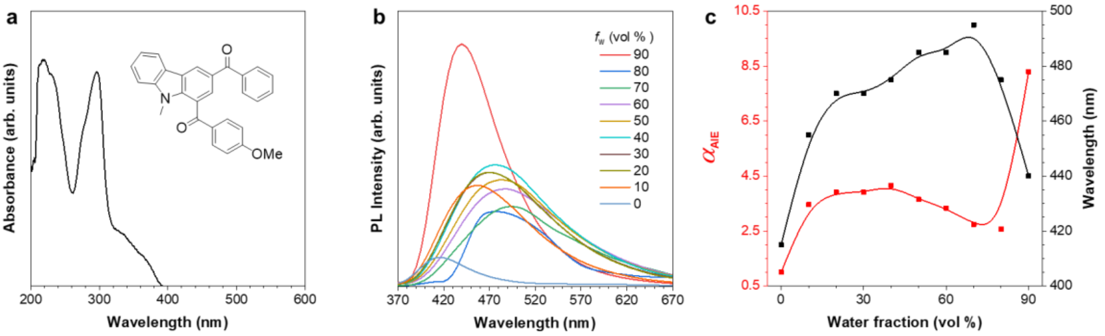

Supplementary Fig. 37 The photophysical property of **3b. a** Absorption spectra of **3b** in pure THF. **b** PL spectra of **3b** in THF/H_2O mixtures with different f_w. λ_{ex}: 296 nm. Concentration: 20 μM. **c** The plots of the α_{AIE} and maximum emission wavelength versus the composition of the aqueous mixture of **3b**, $\alpha_{AIE} = I/I_0$, I_0 = PL intensity in pure THF.

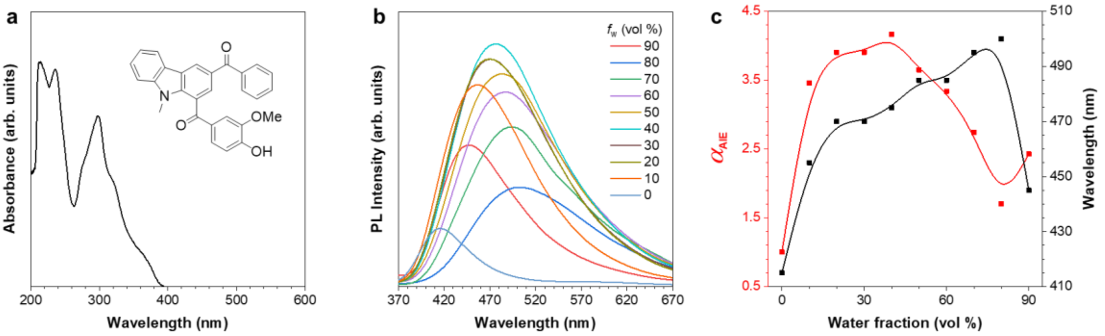

Supplementary Fig. 38 The photophysical property of **3c. a** Absorption spectra of **3c** in pure THF. **b** PL spectra of **3c** in THF/H_2O mixtures with different f_w. λ_{ex}: 298 nm. Concentration: 20 μM. **c** The plots of the α_{AIE} and maximum emission wavelength versus the composition of the aqueous mixture of **3c**, $\alpha_{AIE} = I/I_0$, I_0 = PL intensity in pure THF.

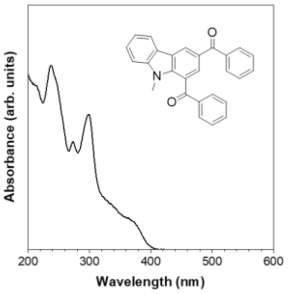

Supplementary Fig. 39 Absorption spectra of **3d** in pure ACN.

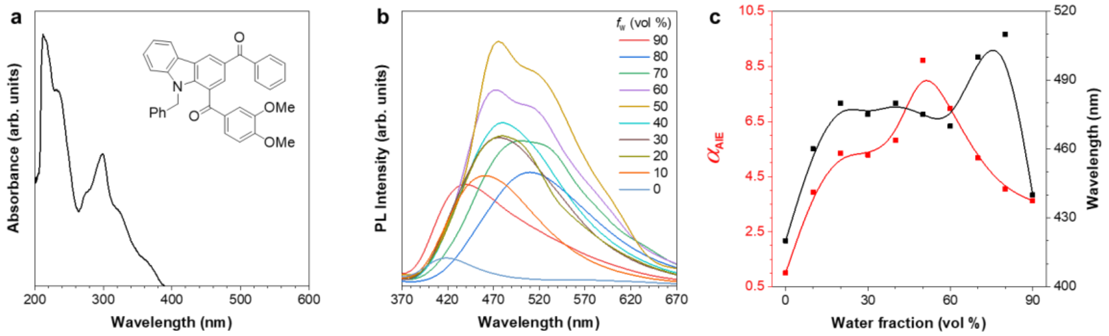

Supplementary Fig. 40 The photophysical property of **3e. a** Absorption spectra of **3e** in pure THF. **b** PL spectra of **3e** in THF/H_2O mixtures with different f_w. λ_{ex}: 298 nm. Concentration: 20 μM. **c** The plots of the α_{AIE} and maximum emission wavelength versus the composition of the aqueous mixture of **3e**, $\alpha_{AIE} = I/I_0$, I_0 = PL intensity in pure THF.

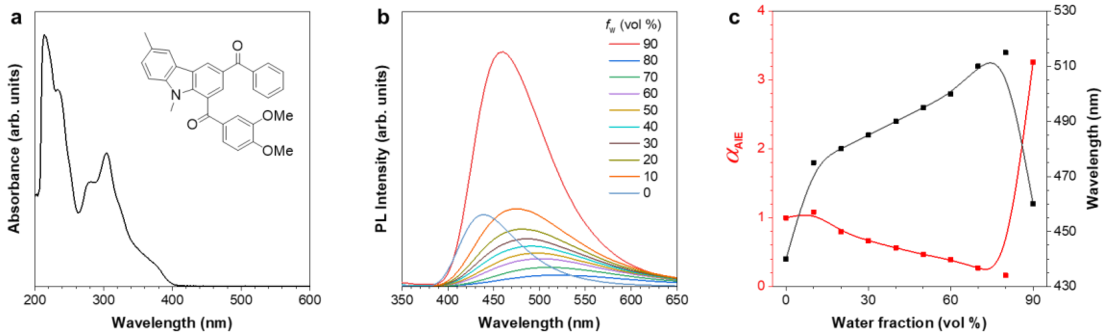

Supplementary Fig. 41 The photophysical property of **3f. a** Absorption spectra of **3f** in pure THF. **b** PL spectra of **3f** in THF/H_2O mixtures with different f_w. λ_{ex}: 304 nm. Concentration: 20 μM. **c** The plots of the α_{AIE} and maximum emission wavelength versus the composition of the aqueous mixture of **3f**, $\alpha_{AIE} = I/I_0$, I_0 = PL intensity in pure THF.

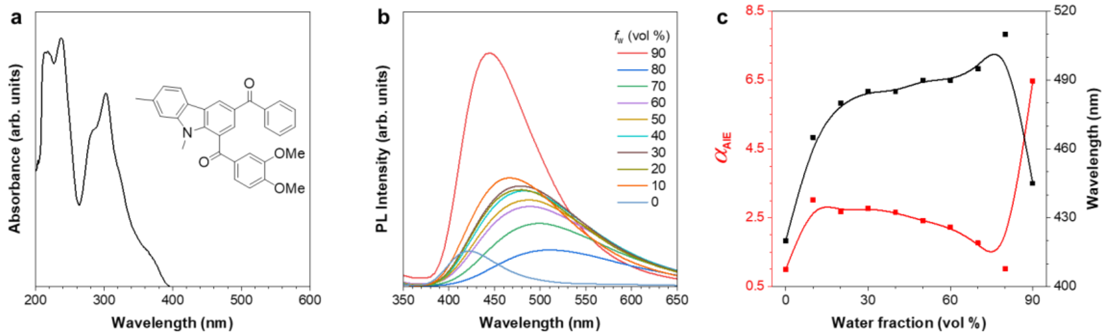

Supplementary Fig. 42 The photophysical property of **3g. a** Absorption spectra of **3g** in pure THF. **b** PL spectra of **3g** in THF/H_2O mixtures with different f_w. λ_{ex}: 303 nm. Concentration: 20 μM. **c** The plots of the α_{AIE} and maximum emission wavelength versus the composition of the aqueous mixture of **3g**, $\alpha_{AIE} = I/I_0$, I_0 = PL intensity in pure THF.

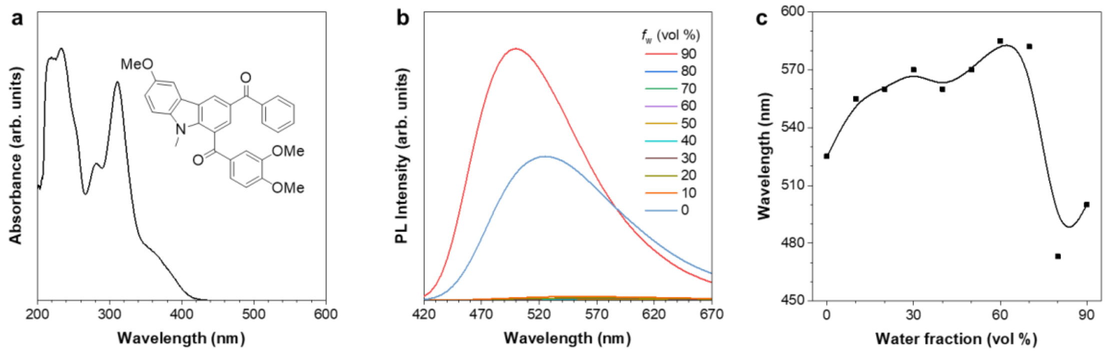

Supplementary Fig. 43 The photophysical property of **3h. a** Absorption spectra of **3h** in pure ACN. **b** PL spectra of **3h** in ACN/H_2O mixtures with different f_w. λ_{ex}: 311 nm. Concentration: 20 μM. **c** The plot of maximum emission wavelength versus the composition of the aqueous mixture of **3h.**

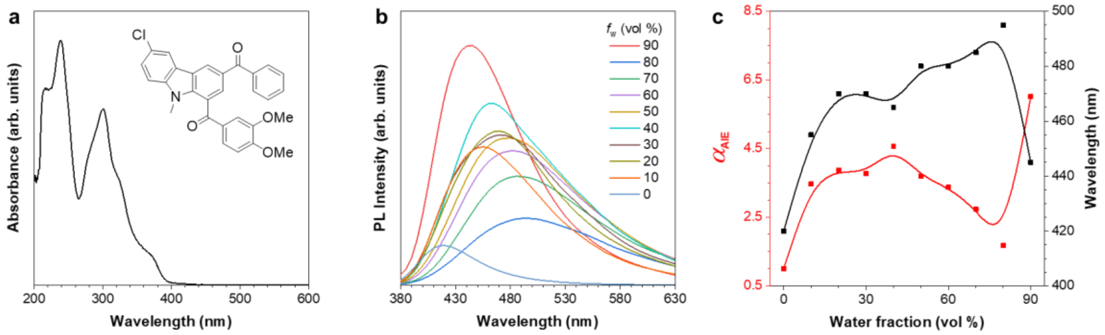

Supplementary Fig. 44 The photophysical property of **3i. a** Absorption spectra of **3i** in pure THF. **b** PL spectra of **3i** in THF/H_2O mixtures with different f_w. λ_{ex}: 300 nm. Concentration: 20 μM. **c** The plots of the α_{AIE} and maximum emission wavelength versus the composition of the aqueous mixture of **3i**, $\alpha_{AIE} = I/I_0$, I_0 = PL intensity in pure THF.

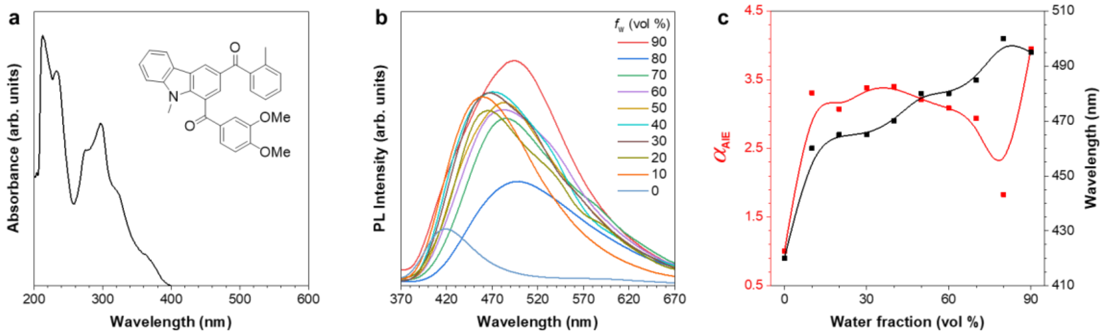

Supplementary Fig.45 The photophysical property of **3j. a** Absorption spectra of **3j** in pure THF. **b** PL spectra of **3j** in THF/H_2O mixtures with different f_w. λ_{ex}: 300 nm. Concentration: 20 μM. **c** The plots of the α_{AIE} and maximum emission wavelength versus the composition of the aqueous mixture of **3j**, $\alpha_{AIE} = I/I_0$, I_0 = PL intensity in pure THF.

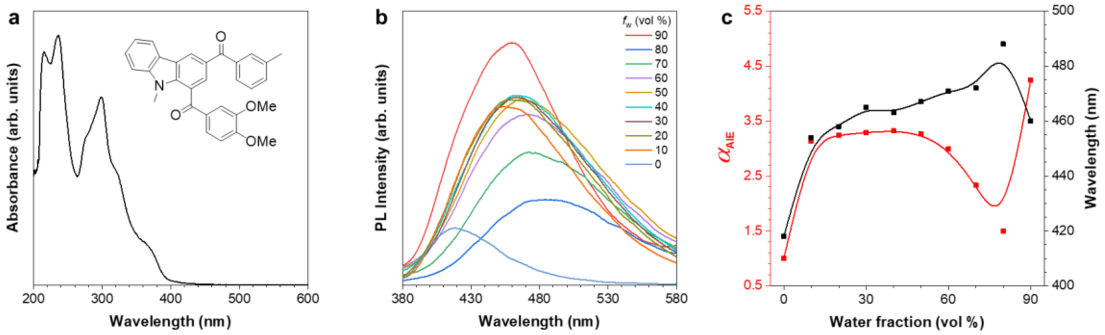

Supplementary Fig. 46 The photophysical property of **3k. a** Absorption spectra of **3k** in pure THF. **b** PL spectra of **3k** in THF/H_2O mixtures with different f_w. λ_{ex}: 299 nm. Concentration: 20 μM. **c** The plots of the α_{AIE} and maximum emission wavelength versus the composition of the aqueous mixture of **3k**, $\alpha_{AIE} = I/I_0$, I_0 = PL intensity in pure THF.

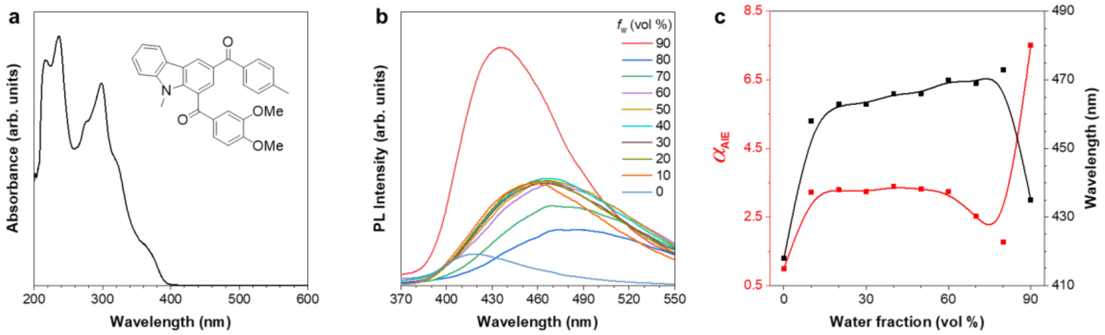

Supplementary Fig. 47 The photophysical property of **3l. a** Absorption spectra of **3l** in pure THF. **b** PL spectra of **3l** in THF/H_2O mixtures with different f_w. λ_{ex}: 298 nm. Concentration: 20 μM. **c** The plots of the α_{AIE} and maximum emission wavelength versus the composition of the aqueous mixture of **3l**, $\alpha_{AIE} = I/I_0$, I_0 = PL intensity in pure THF.

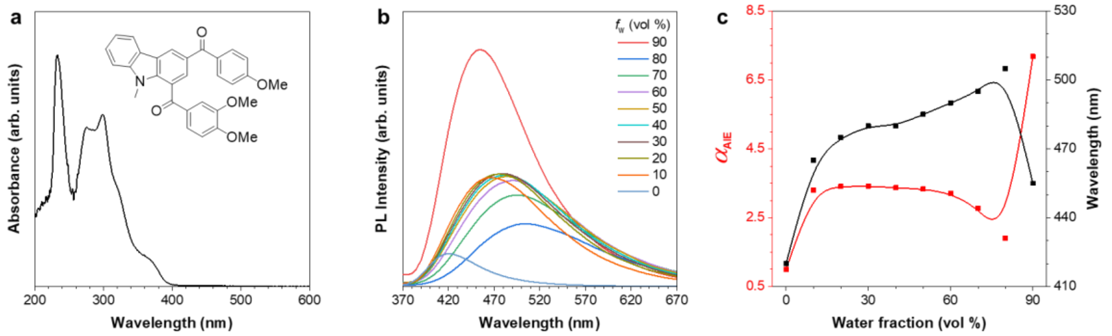

Supplementary Fig. 48 The photophysical property of **3m. a** Absorption spectra of **3m** in pure THF. **b** PL spectra of **3m** in THF/H_2O mixtures with different f_w. λ_{ex}: 299 nm. Concentration: 20 μM. **c** The plots of the α_{AIE} and maximum emission wavelength versus the composition of the aqueous mixture of **3m**, $\alpha_{AIE} = I/I_0$, I_0 = PL intensity in pure THF.

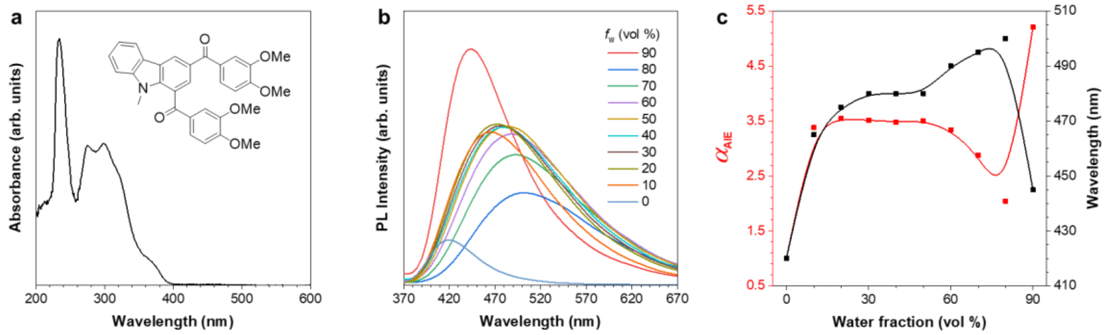

Supplementary Fig. 49 The photophysical property of **3n. a** Absorption spectra of **3n** in pure THF. **b** PL spectra of **3n** in THF/H_2O mixtures with different f_w. λ_{ex}: 300 nm. Concentration: 20 μM. **c** The plots of the α_{AIE} and maximum emission wavelength versus the composition of the aqueous mixture of **3n**, $\alpha_{AIE} = I/I_0$, I_0 = PL intensity in pure THF.

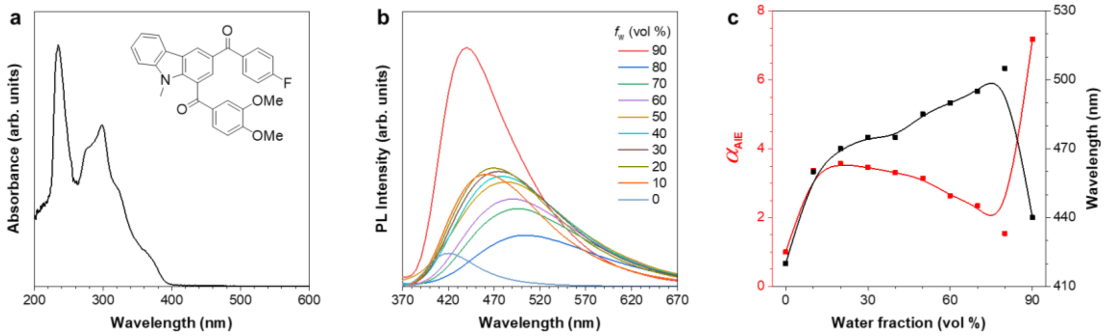

Supplementary Fig. 50 The photophysical property of **3o. a** Absorption spectra of **3o** in pure THF. **b** PL spectra of **3o** in THF/H_2O mixtures with different f_w. λ_{ex}: 297 nm. Concentration: 20 μM. **c** The plots of the α_{AIE} and maximum emission wavelength versus the composition of the aqueous mixture of **3o**, $\alpha_{AIE} = I/I_0$, I_0 = PL intensity in pure THF.

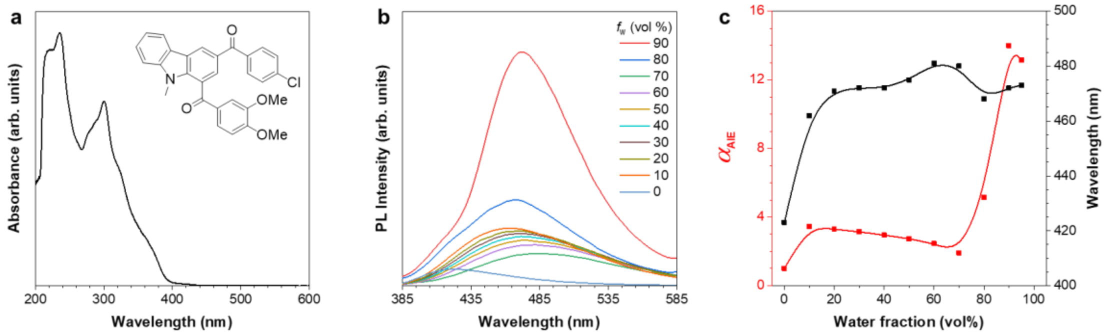

Supplementary Fig. 51 The photophysical property of **3p. a** Absorption spectra of **3p** in pure THF. **b** PL spectra of **3p** in THF/H_2O mixtures with different f_w. λ_{ex}: 300 nm. Concentration: 20 μM. **c** The plots of the α_{AIE} and maximum emission wavelength versus the composition of the aqueous mixture of **3p**, $\alpha_{AIE} = I/I_0$, I_0 = PL intensity in pure THF.

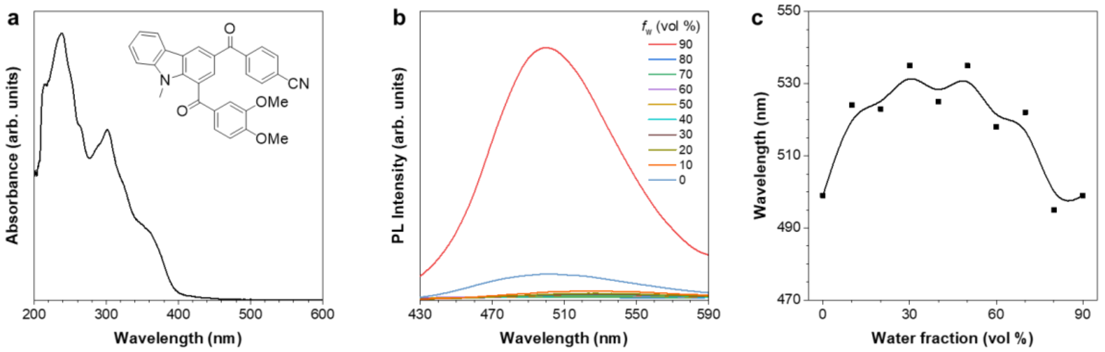

Supplementary Fig. 52 The photophysical property of **3q. a** Absorption spectra of **3q** in pure THF. **b** PL spectra of **3q** in THF/H_2O mixtures with different f_w. λ_{ex}: 301 nm. Concentration: 20 μM. **c** The plot of maximum emission wavelength versus the composition of the aqueous mixture of **3q**.

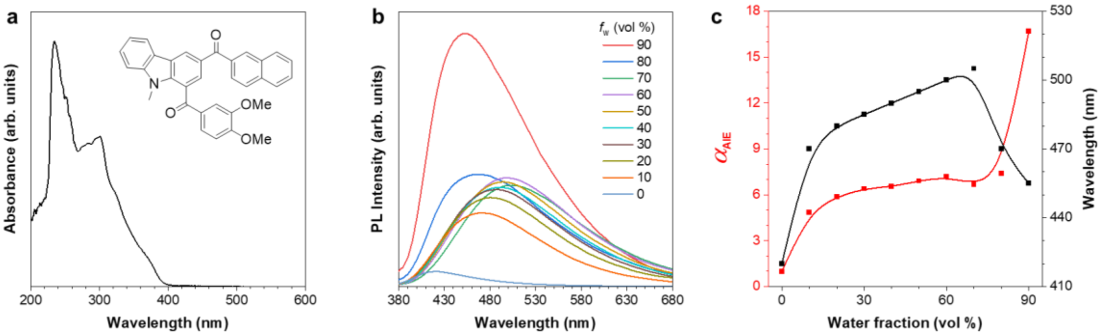

Supplementary Fig. 53 The photophysical property of **3r. a** Absorption spectra of **3r** in pure THF. **b** PL spectra of **3r** in THF/H_2O mixtures with different f_w. λ_{ex}: 302 nm. Concentration: 20 μM. **c** The plots of the α_{AIE} and maximum emission wavelength versus the composition of the aqueous mixture of **3r**, $\alpha_{AIE} = I/I_0$, I_0 = PL intensity in pure THF.

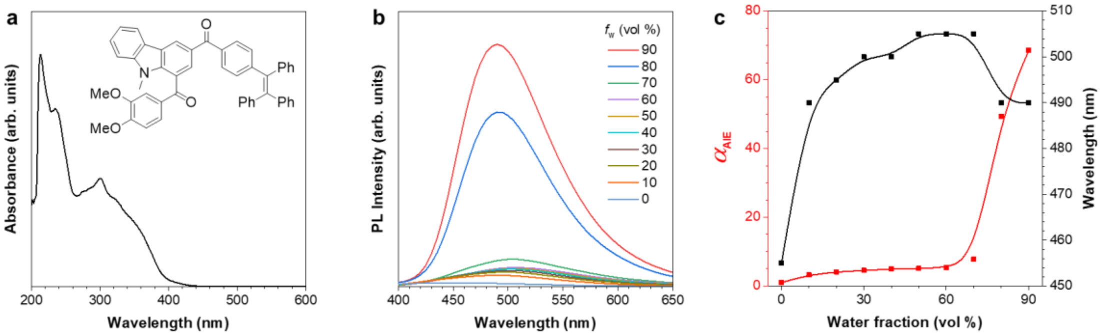

Supplementary Fig. 54 The photophysical property of **3s. a** Absorption spectra of **3s** in pure THF. **b** PL spectra of **3s** in THF/H_2O mixtures with different f_w. λ_{ex}: 302 nm. Concentration: 20 μM. **c** The plots of the α_{AIE} and maximum emission wavelength versus the composition of the aqueous mixture of **3s**, $\alpha_{AIE} = I/I_0$, I_0 = PL intensity in pure THF.

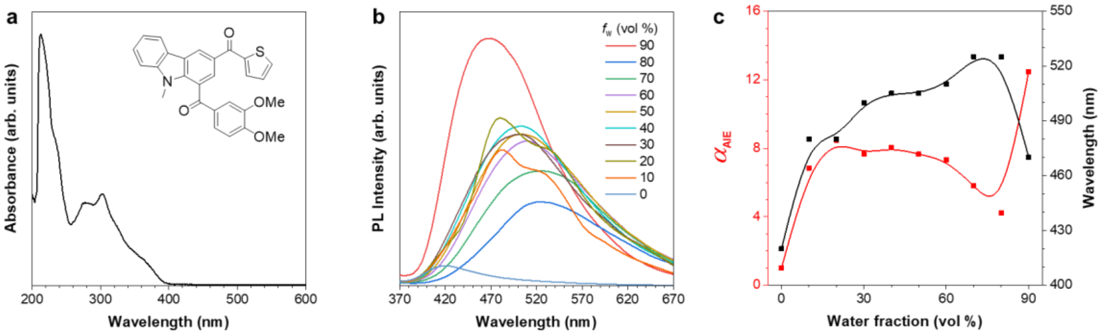

Supplementary Fig. 55 The photophysical property of **3t. a** Absorption spectra of **3t** in pure THF. **b** PL spectra of **3t** in THF/H_2O mixtures with different f_w. λ_{ex}: 302 nm. Concentration: 20 μM. **c** The plots of the α_{AIE} and maximum emission wavelength versus the composition of the aqueous mixture of **3t**, $\alpha_{AIE} = I/I_0$, I_0 = PL intensity in pure THF.

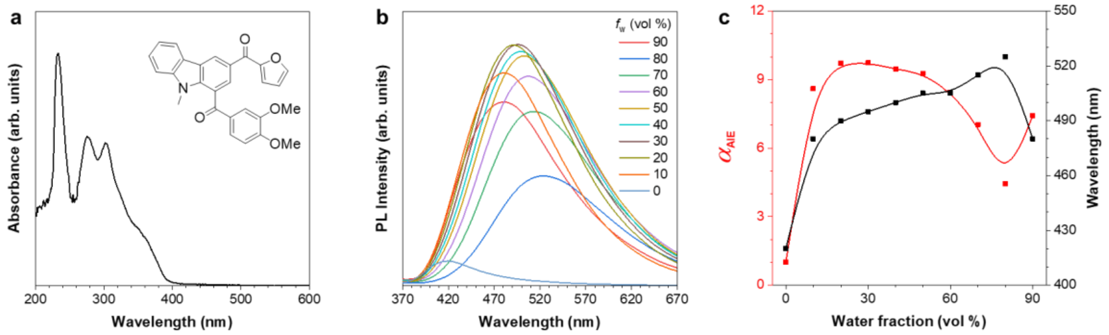

Supplementary Fig. 56 The photophysical property of **3u. a** Absorption spectra of **3u** in pure THF. **b** PL spectra of **3u** in THF/H_2O mixtures with different f_w. λ_{ex}: 302 nm. Concentration: 20 μM. **c** The plots of the α_{AIE} and maximum emission wavelength versus the composition of the aqueous mixture of **3u**, α_{AIE} = I/I_0, I_0 = PL intensity in pure THF.

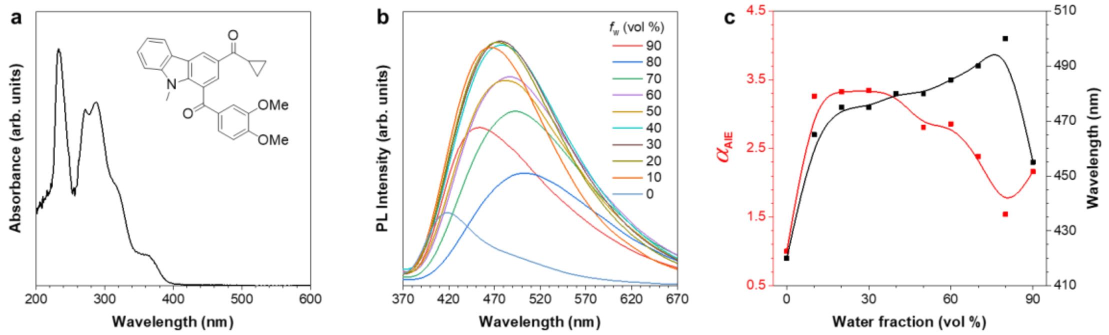

Supplementary Fig. 57 The photophysical property of **3v. a** Absorption spectra of **3v** in pure THF. **b** PL spectra of **3v** in THF/H_2O mixtures with different f_w. λ_{ex}: 300 nm. Concentration: 20 μM. **c** The plots of the α_{AIE} and maximum emission wavelength versus the composition of the aqueous mixture of **3v**, α_{AIE} = I/I_0, I_0 = PL intensity in pure THF.

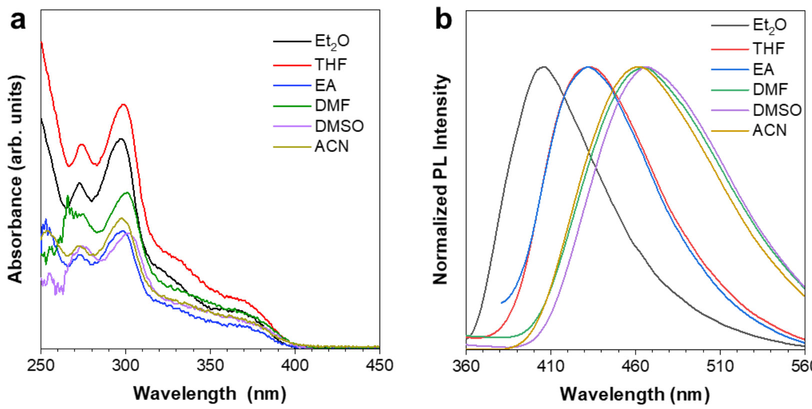

Supplementary Fig. 58 The photophysical property of **3d** in different solvents**.** Absorption **a** and PL **b** spectra of **3d** in solvents with different polarities. Concentration: 20 μM. The absorption maximum of each solution was chosen as its excitation wavelength.

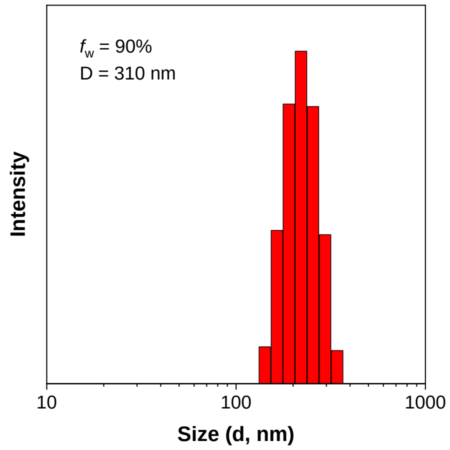

Supplementary Fig. 59 DLS results of **3d** in ACN/H_2O mixtures with f_w = 90% (20 μM).

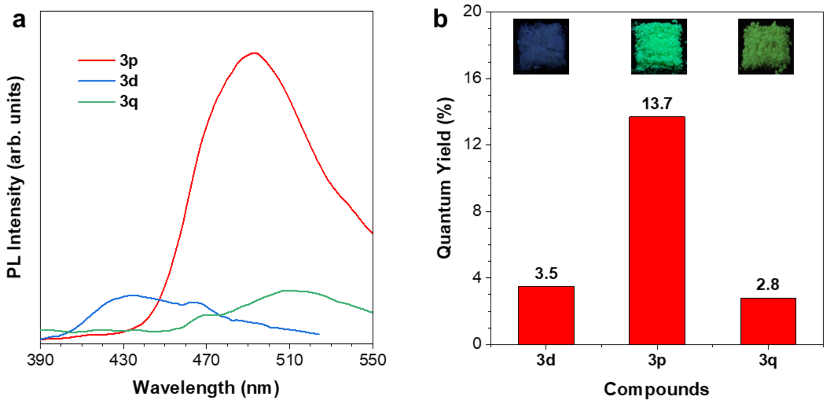

Supplementary Fig. 60 The photophysical property of **3d**, **3p**, and **3q** as solid. PL spectra (**a**) and QY data (**b**) of **3d**, **3p** and **3q**. Inset: photographs of **3d**, **3p**, and **3q** as solid taken under 365 nm UV light. The absorption maximum of each solution was chosen as its excitation wavelength.

Supplementary Table 5. Crystallographic data for compounds **3d**, **3p**, and **3q**.

	**3d**	**3p**	**3q**
empirical formula	$C_{29}H_{22}N_2O_2$	$C_{29}H_{22}ClNO_4$	$C_{30}H_{22}N_2O_4$
M_r	430.48	483.92	474.49
cryst syst	orthorhombic	triclinic	monoclinic
space group	*-P b c a(61)*	$P\bar{1}(2)$	$P2_1/n$
a (Å)	16.6859(6)	10.1786(3)	9.24590(10)
b (Å)	8.1266(3)	10.6245(5)	17.7543(2)
c (Å)	33.2521(11)	12.0390(5)	15.1236(2)
α (°)	90	87.022(4)	90
β (°)	90	71.598(3)	107.148(2)
γ (°)	90	73.459(4)	90
V (Å^3)	4509.0(3)	1183.21(9)	2372.25(5)
Z	8	2	4
ρ_c (g cm^{-3})	1.268	1.358	1.329
F (000)	1808	504.0	992.0
T (K)	293(2)	295.24(10)	295.32(10)
μ (mm^{-1})	0.634	1.733	0.721
data / restraints / parameters	4004/0/301	4734/0/320	4766/0/329
GOF (F^2)	1.117	1.088	1.095
R_1[a], wR_2[b] ($I > 2\sigma(I)$)	0.1268, 0. 1356	0.0725, 0.2119	0.0502 , 0.1504
R_{int}	0.0393	0.0356	0.0492

[a] $R_1 = \Sigma(||F_o| - |F_c||)/\Sigma|F_o|$; [b] $wR_2 = \{\Sigma[w(F_o^2 - F_c^2)^2]/\Sigma[w(F_o^2)^2]\}^{1/2}$

3. Supplementary references

1.Tian, H.-R., Liu, Y. W., Zhang, Z., Liu, S. M., Dang, T. Y., Li, X. H., Sun, X. W., Lu, Y. & Liu, S. X. A Multicentre synergistic polyoxometalate-based metal–organic framework for one-step selective oxidative cleavage of β-O-4 lignin model compounds. *Green Chem.* **22**, 248-255 (2020).

2. Son, S. & Toste, F. D. Non-oxidative vanadium-catalyzed C-O bond cleavage: application to degradation of lignin model compounds. *Angew. Chem. Int. Ed.* **49**, 3791-3794 (2010).

3. Zhang, Y.-L., Qin, Y.-J., Tang, D.-J., Yang, M.-R., Li, B.-Y., Wang, Y.-T., Cai, H.-Y., Wang, B.-Z. & Zhu, H.-L. Synthesis and biological evaluation of 1-methyl-1H-indole-pyrazoline hybrids as potential tubulin polymerization inhibitors. *ChemMedChem* **11**, 1446-1458 (2016).
