# Supplementary material for: Towards bioresource-based aggregation-induced emission luminogens from lignin β-O-4 motifs as renewable resources
Source: Nat Commun. 2023 Sep 28;14:6076. doi: 10.1038/s41467-023-41681-0 (PMC10539282; doi:10.1038/s41467-023-41681-0)
Supplement: Supplementary file 2 — Description of Additional Supplementary Files [file 41467_2023_41681_MOESM2_ESM.pdf]

Description of Additional Supplementary Files

File Name: Supplementary Data 1
Description: Cartesian coordinates
